# Approximate Bayesian inference in semi-mechanistic models

**DOI:** 10.1007/s11222-016-9668-8

**Published:** 2016-06-16

**Authors:** Andrej Aderhold, Dirk Husmeier, Marco Grzegorczyk

**Affiliations:** 1 School of Mathematics and Statistics, Glasgow University, Glasgow, UK; 2 Johann Bernoulli Institute (JBI), Groningen University, Groningen, The Netherlands

**Keywords:** Network Inference, Semi-mechanistic model, Bayesian model selection, Widely applicable information criteria (WAIC, WBIC), Markov jump processes, ANOVA, Systems biology

## Abstract

Inference of interaction networks represented by systems of differential equations is a challenging problem in many scientific disciplines. In the present article, we follow a semi-mechanistic modelling approach based on gradient matching. We investigate the extent to which key factors, including the kinetic model, statistical formulation and numerical methods, impact upon performance at network reconstruction. We emphasize general lessons for computational statisticians when faced with the challenge of model selection, and we assess the accuracy of various alternative paradigms, including recent widely applicable information criteria and different numerical procedures for approximating Bayes factors. We conduct the comparative evaluation with a novel inferential pipeline that systematically disambiguates confounding factors via an ANOVA scheme.

## Introduction

A topical and challenging problem for computational statistics and machine learning is to infer the structure of complex systems of interacting units. This research area has been particularly motivated by the cognate research discipline of computational systems biology, where researchers aim to reconstruct the structure of biopathways or regulatory networks from postgenomic data; see e.g. Smolen et al. (2000), De Jong (2002) and Lawrence et al. (2010). Two principled approaches can be distinguished. The first paradigm aims to apply generic models like sparse Lasso-type regression, Bayesian networks, or hierarchical Bayesian models. A recent overview and comparative evaluation was published by Aderhold et al. (2014). The advantage of this approach is that the computational complexity of inference is comparatively low, and the application of these methods to problems of genuine interest is computationally feasible. The disadvantage is that interactions are modelled at a high level of abstraction, which ignores the detailed nature of the underlying mechanisms. The second paradigm is based on mechanistic models and the detailed mathematical description of the underlying interaction processes, typically in the form of ordinary or stochastic differential equations (DEs). Two pioneering examples of this approach were published by Vyshemirsky and Girolami (2008) and Toni et al. (2009). The advantage of this paradigm is a more detailed and faithful mathematical representation of the interactions in the system. The disadvantage is the substantially higher computational costs of inference, which stem from the fact that each parameter adaptation requires a numerical integration of the differential equations. A novel approach, presented by Oates et al. (2014) and termed ’chemical model averaging’ (CheMA), aims for a compromise that combines the strengths of both paradigms. The underlying principle is that of gradient matching, first proposed by Ramsay et al. (2007). Given the concentration time series of some quantities whose interactions are to be inferred, the temporal derivatives of the concentrations are directly estimated from the data. These derivatives are then matched against those predicted from the DEs. Formally, on the assumption that the mismatch can be treated like observational noise of known distributional form, we can derive the likelihood and thus apply standard statistical inference techniques. The model is effectively a non-linear regression model, whose computational complexity of inference sits between the two paradigms discussed above: it is lower than for proper mechanistic models, since the DEs do not have to be integrated numerically; it is higher than for standard models of the first category, since the model is non-linear in its parameters and an analytic marginalization is intractable. Henceforth, we refer to it as a ’semi-mechanistic’ model.

**Fig. 1:**
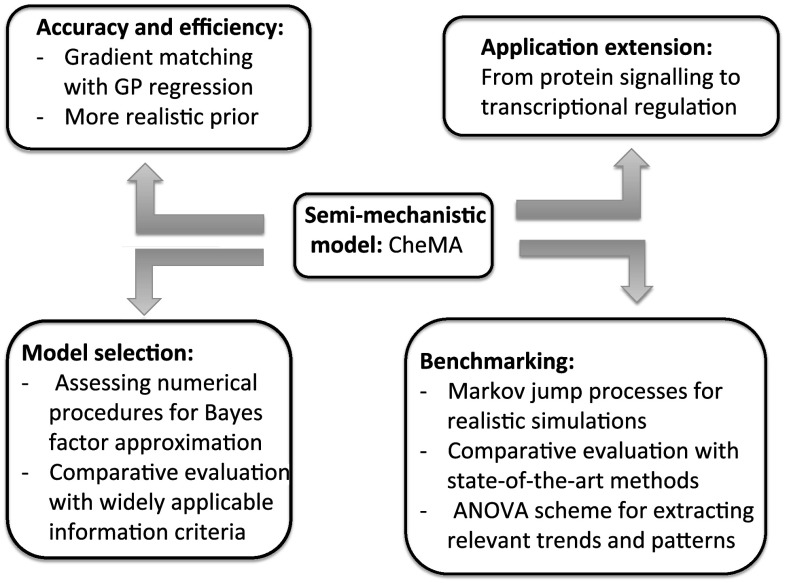
Overview of the presented work and how it extends the CheMA model of Oates et al. (2014)

This article takes the work of Oates et al. (2014), which won the best paper award at the European Conference on Computational Biology (ECCB) in 2014, further in four respects, related to *accuracy and efficiency*, *model selection*, *benchmarking*, and *application expansion*. An overview can be found in Fig. 1.

### Accuracy and efficiency

Robust gradient estimation is absolutely critical for semi-mechanistic modelling. The numerical differentiation proposed in Oates et al. (2014) is known to be susceptible to noise amplification. We here propose the application of Gaussian process (GP) regression and the exploitation of the fact that under fairly general assumptions, GPs are closed under differentiation. Our approach effectively implements a low-pass filter that counteracts the noise amplification of the differentiation step, and we quantify the boost in network reconstruction accuracy that can be achieved in this way. We further critically assess the influence of the parameter prior in the underlying Bayesian hierarchal model. In particular, we compare the g-prior with the ridge regression prior (see e.g. Chapter 3 in Marin and Robert (2007)) in the context of the proposed semi-mechanistic model and demonstrate that the latter significantly improves both accuracy and computational efficiency.

### Model selection

Network reconstruction is effectively based on statistical model selection. The model selection paradigm applied in Oates et al. (2014)—computing the log marginal likelihood (MLL) with Chib’s method—is not uncontroversial. Conceptually, alternatives to the MLL based on predictive performance have been promoted (see e.g. Sect. 7.4 in Gelman et al. (2014a)). Numerically, Chib’s method can give inaccurate results, as discussed e.g. in Murphy (2012), Chapter 24. In this article, we assess four numerical approximation procedures for the MLL in the context of semi-mechanistic models: Chib’s original method (Chib and Jeliazkov 2001), Chib’s method with a numerical stabilization, thermodynamic integration (Friel and Pettitt 2008), and a numerically stabilized version of thermodynamic integration (Friel et al. 2013). We further carry out a comparative evaluation between the MLL and four information criteria (IC) as approximations to the predictive performance paradigm promoted in Gelman et al. (2014a): ’divergence IC’ (DIC), ’widely applicable IC’ (WAIC), ’cross-validation IC’ (CVIC), and ’widely applicable Bayesian IC’ (WBIC).

**Fig. 2:**
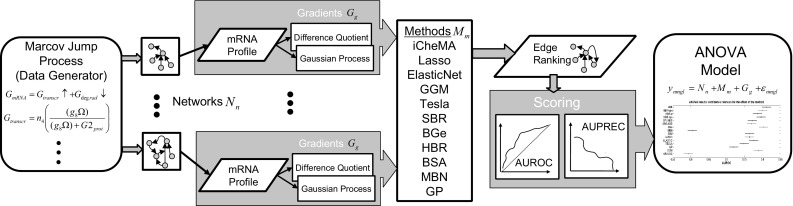
Overview of the ANOVA pipeline. Based on a mathematical formulation in terms of Markov jump processes, realistic mRNA concentration time series are generated from a collection of gene regulatory networks, and the transcription rate (temporal gradient) is computed with two alternative methods: numerical differentiation versus GP regression. The proposed iCheMA model is compared with 10 established state-of-the-art network reconstruction methods, each predicting a ranking of the potential interactions (edges) in the network. From these rankings, the areas under the ROC (AUROC) and precision-recall curve (AUPRC) are computed, and provide a score for the accuracy of network reconstruction. An ANOVA scheme is applied to disentangle the effects of network topology, gradient computation and reconstruction method, for clearer recognition of trends and patterns

### Benchmarking

Assessing methodological innovation calls for an objective performance evaluation. We have carried out a comprehensive comparative evaluation of the proposed semi-mechanistic model with 11 state-of-the-art network inference methods from computational statistics and machine learning, based on a realistic stochastic process model of the underlying molecular processes (Guerriero et al. 2012) and six distinct regulatory networks with different degrees of connectivity. The analysis of such a complex simulation study is hampered by the influence of various confounding factors, which tend to blur naive graphical representations. We therefore apply an ANOVA scheme, which enables us to disentangle the various effects and thereby extract clear trends and patterns in the results. In this way we can show that by integrating prior domain knowledge via a system-specific mathematical representation, the resulting semi-mechanistic model can significantly outperform state-of-the-art generic machine learning and computational statistics methods. We provide an application pipeline (Fig. 2) with which a user can objectively quantify this performance gain.

### Application extension

Finally, we adapt the method from the modelling of protein signalling cascades in Oates et al. (2014) to transcriptional gene regulation and include an explicit model of transcriptional time delays. In Appendix 4 we provide a novel application of the proposed semi-mechanistic model to plant systems biology, where the objective is to infer the structure of the key gene regulatory network controlling circadian regulation in *Arabidopsis thaliana*.

**Fig. 3:**
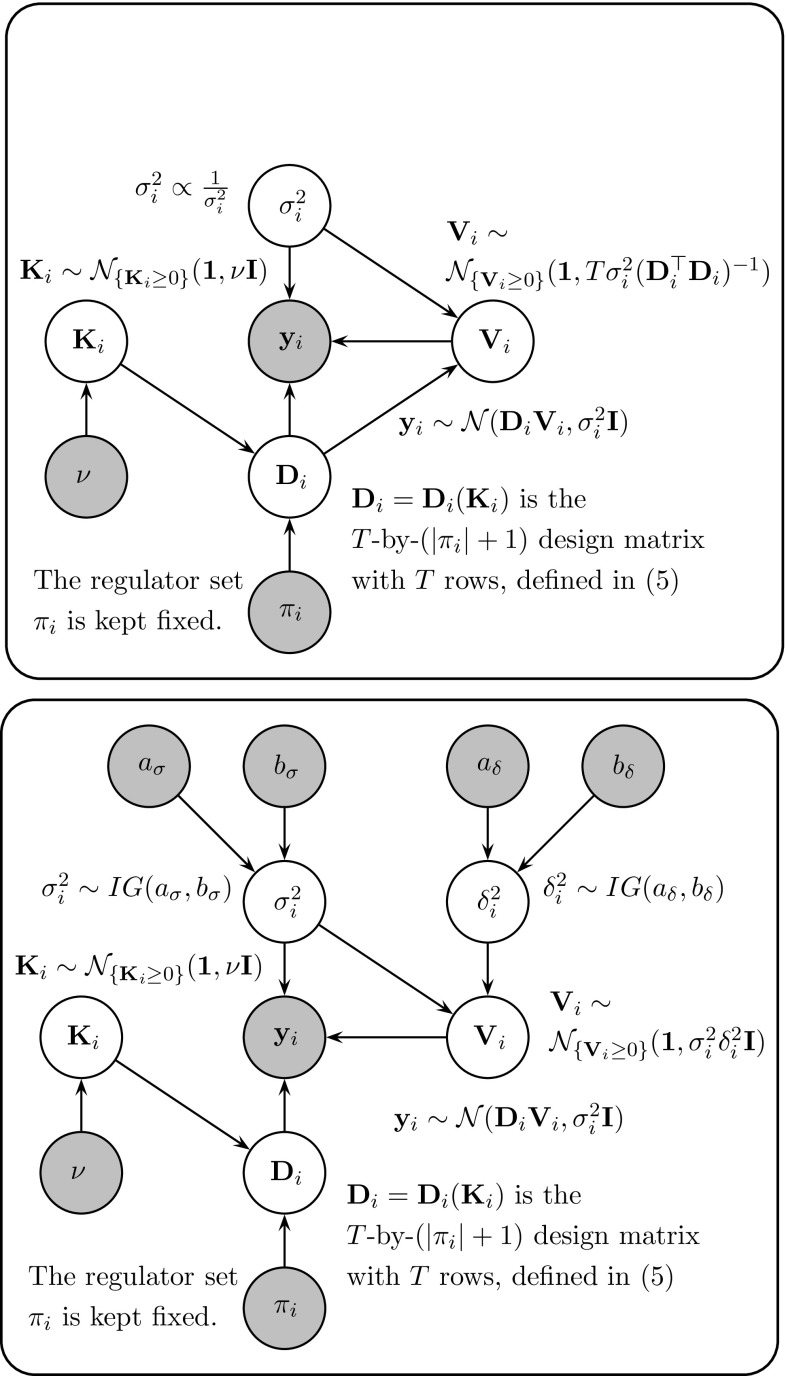
Probabilistic graphical model representation of semi-mechanistic models. The figure shows a probabilistic graphical model representation of the semi-mechanistic models investigated in our study. *Top panel* CheMA, as proposed by Oates et al. (2014). *Bottom panel* The new variant of CheMA (iCheMA), proposed here

## Model

### Interaction model

Inference of networks from data has become a topical theme in various scientific disciplines, particularly in systems biology. Here, rather than merely aiming for a descriptive representation of associations, the objective is a quantitative mathematical description of the processes that lead to the formation of an interaction network (e.g. a ‘biopathway’ or a ‘biochemical reaction network’). A standard approach is to model this network with a system of ordinary differential equations (ODEs):

1 $$\begin{aligned} \frac{d x_i(t)}{dt}|_{t=t^{\star }} = F_i(\pi _i(t^{\star }),\varvec{\theta }) \end{aligned}$$

Here, $i\in \{1,\ldots ,n\}$ denotes one of *n* components of the system, which is called a ‘node’. In systems biology, this is typically a gene or a protein. The variable $x_i(t)$ denotes a measurable concentration of node *i* at time *t*. This can, for instance, be a gene expression or mRNA concentration. The vector $\pi _i(t)$ contains the concentrations of the regulators of node *i*. In a network, the regulators of node *i* are those nodes with a directed edge (or arrow) pointing to node *i*. Finally, the differential equations depend on a parameter vector $\varvec{\theta }$. In systems biology, these parameters are typically reaction rates that determine the kinetics of the underlying reactions. A specific example, taken from Barenco et al. (2006), is given in Sect. 2.3. What makes network inference in the context of such a mechanistic description particularly challenging is the fact that the parameters $\varvec{\theta }$ are typically not measurable, or that only a small fraction of them can be measured. Hence, the elucidation of the interaction network structure requires these parameters to be inferred from concentration time series, which are typically sparse and noisy. To avoid the computational complexity of numerically solving the ODEs, we follow Oates et al. (2014) and use gradient matching. The idea, first proposed by Ramsay et al. (2007), is to estimate the time derivatives $\frac{d x_i}{dt}$ directly from the data, then treat the problem as nonlinear regression. On the assumption that the estimated derivatives can be treated like noisy data distributed around the predicted derivatives, and this distribution is iid normal, we obtain for the likelihood:

2 $$\begin{aligned} { p(D|\varvec{\theta }) = \prod _{i=1}^n \prod _{j=1}^T \mathscr {N}(y_i(t_j)|F_i(\pi _i(t_j),\varvec{\theta }), \sigma ^2_i) } \end{aligned}$$

where $y_i(t_j)= \frac{d x_i(t)}{dt}|_{t=t_j}$, and $\mathscr {N}(.|\mu ,\sigma ^2)$ is a normal distribution with mean $\mu$ and variance $\sigma ^2$. Oates et al. (2014) obtained the temporal derivatives $y_i(t_j)$ ($j=1,\ldots ,T$) by differencing the time series $x_i(t_1),\ldots ,x_i(t_T)$, based on the Euler equation. However, differencing is known to lead to noise amplification (see e.g. Chatfield (1989)). In the present work, we apply a GP to smooth interpolation and exploit the fact that GPs are closed under differentiation, i.e. provided the kernel is differentiable, the derivative of a GP is also a GP, and its covariance matrix can be derived (Solak et al. 2002; Holsclaw et al. 2013).^1^ We provide more details in the following section.

### Rate (or gradient) estimation

The fundamental concept of the interaction model is the matching of gradients between the regulator variables on the right-hand side and the rate of mRNA concentration change $\frac{dx_i(t)}{dt}$ on the left-hand side of Eq. (1). Since direct measurements of these rates are typically missing, we derive a rate estimate from the available concentration measurements at the time points $t^{\star }\in \{t_1,\ldots ,t_T\}$. A common procedure, which is also used by Oates et al. (2014), is to calculate the slope of the concentration change at each time point $t^{\star }$ with the finite difference quotients

3 $$\begin{aligned} \frac{dx_i(t)}{dt}\Bigg |_{t=t^{\star }} \approx \frac{x_i(t^{\star }+\delta _t) - x_i(t^{\star }-\delta _t)}{2 \delta _t} \end{aligned}$$

This numerical procedure can yield good approximations to the true rates of concentration change if the data $x_i$ is relatively precise, i.e. the signal-to-noise ratio is high. If the data is noisy, however, the rates from the difference quotient are susceptible to distortions as a consequence of noise amplification, as mentioned above. We here propose the application of GP regression to counteract this noise amplification. A GP defines a prior distribution over functions *g*(.) that transform input data points, defined here as time points $\mathbf{t}= (t_1, \ldots , t_T)$, into output data points, defined here as the concentration vector $\mathbf{x}_i= (x_i(t_1), \ldots , x_i(t_T))$ for species *i* such that $x_i(t^*) = g(t^*)$. The joint prior distribution over the functions $p(g(t_1), \ldots , g(t_T))$ is Gaussian distributed and commonly has a zero mean and a covariance matrix $\mathbf{G}$ with independent and identically distributed (iid) additive noise $\sigma ^2_n$:

4 $$\begin{aligned} p(\mathbf{g} | \mathbf{t}) = \mathscr {N}(\mathbf{0}, \mathbf{G}+ \sigma ^2_n \mathbf{I}) \end{aligned}$$

where **I** is the identity matrix. The key idea of the GP is that the elements $p, q \in \{1, \ldots , T\}$ of the covariance matrix $\mathbf{G}$ are calculated from a kernel function with $G_{pq} = \kappa (t_p, t_q)$, which is typically chosen in such a way that for similar points $t_p$ and $t_q$, the corresponding values $x_i(t_p)$ and $x_i(t_q)$ are stronger correlated than for dissimilar arguments. Widely used kernel functions that are also applied in this paper are the radial basis function (RBF), the periodic function (PER), or the Matérn class function (MAT); see Chapter  4 in Rasmussen and Williams (2006) for the explicit mathematical expressions. By taking the first derivative of the kernel function $\kappa '(t_p, t_q)$ we obtain a prior distribution $p(\mathbf{g})$ over functions that define the first temporal derivative, i.e. the concentration gradients, for each of the time points in $\mathbf{t}$. Provided the kernel function is differentiable, this is again a valid GP. The simplest approach is to compute the expectation over these functions and thus obtain a mean estimate of the analytical solution for the gradients at each time point. For the explicit mathematical expression, see e.g. Eq. (1) in Holsclaw et al. (2013). This acts as a proxy for the missing rates $y_i(t)$ on the left-hand side of Eq. (1).^2^ In Appendix 2 we describe the details of the GP application and the software we used.

### Model and prior distributions

Equation (1) typically takes the form (Barenco et al. 2006)

5 $$\begin{aligned} \frac{d x_i(t)}{dt}|_{t=t^{\star }} \; = \; c_i - v_{0,i} x_i(t^{\star }) + f_i( \pi _i(t^{\star }),\varvec{\theta }) \end{aligned}$$

Setting $c_i=0$ and employing Michaelis–Menten kinetics yields the CheMA approach (Oates et al. 2014):^3^

6 $$\begin{aligned} \frac{d x_i(t)}{dt}|_{t=t^{\star }}= & {} -v_{0,i} x_i(t^{\star }) \nonumber \\&+\sum _{u\in \pi _i} v_{u,i} \frac{I_{u,i} x_u(t^{\star })+(1-I_{u,i}) k_{u,i}}{x_u(t^{\star })+k_{u,i}} \end{aligned}$$

where the sum is over all species *u* that are in the set of regulators $\pi _i$ of species *i*, and the indicator functions $I_{u,i}$ indicate whether species *u* is an activator ($I_{u,i}=1$) or inhibitor ($I_{u,i}=0$). The first term, $-v_{0,i} x_i(t^{\star })$, takes the degradation of $x_i$ into account, while $v_{u,i}$ and $k_{u,i}$ are the *maximum reaction rate* and *Michaelis–Menten* parameters for the regulatory effect of species $u\in \pi _i$ on species *i*, respectively. Equation (6) represents the typical form of transcriptional regulation without complex formation; see e.g. the supplementary material in Pokhilko et al. (2010) and Pokhilko et al. (2012). We discuss the limitations caused by complex formation in Sect. 6.5. Without loss of generality, we now assume that $\pi _i$ is given by $\pi _i= \{x_1,\ldots ,x_s\}$. Eq. (6) can then be written in vector notation:

7 $$\begin{aligned} \frac{d x_i(t)}{dt}|_{t=t^{\star }} = {\mathbf{D}}_{i,t^{\star }}^{\top } {\mathbf{V}}_i \end{aligned}$$

where ${\mathbf{V}}_i=(v_{0,i},v_{1,i}\ldots ,v_{s,i})^{\top }$ is the vector of the maximum reaction rate parameters, and the vector ${\mathbf{D}}_{i,t^{\star }}$ depends on the measured concentrations $x_u(t^{\star })$ and the Michaelis–Menten parameters $k_{u,i}$ ($u\in \pi _i$) via Eq. (6):

8 $$\begin{aligned} {\mathbf{D}}_{i,t^{\star }}^{\top }= & {} \left( -x_i(t^{\star }),\frac{I_{1,i} x_1(t^{\star })+(1-I_{1,i}) k_{1,i}}{x_1(t^{\star })+k_{1,i}},\right. \nonumber \\&\left. \ldots ,\frac{I_{s,i} x_{s}(t^{\star })+(1-I_{s,i}) k_{s,i}}{x_s(t^{\star })+k_{s,i}} \right) \end{aligned}$$

We combine the Michaelis–Menten parameters $k_{u,i}$ ($u\in \pi _i$) in a vector ${\mathbf{K}}_i$, and we arrange the *T* row vectors ${\mathbf{D}}_{i,t^{\star }}^{\top }$ ($t^{\star }\in \{t_1,\ldots ,t_T\}$) in a *T*-by-$(|\pi _i|+1)$ design matrix ${\mathbf{D}}_i={\mathbf{D}}_i({\mathbf{K}}_i)$. The likelihood is then:

9 $$\begin{aligned} p({\mathbf{y}}_i|{\mathbf{K}}_i,{\mathbf{V}}_i,\sigma _i^2)= (2\pi \sigma _i^2)^{-\frac{T}{2}} e^{-\frac{1}{2\sigma ^2_{i}} ({\mathbf{y}}_i - {\mathbf{D}}_i {\mathbf{V}}_i)^{\top } ({\mathbf{y}}_i - {\mathbf{D}}_i {\mathbf{V}}_i)} \end{aligned}$$

where ${\mathbf{y}}_i:=(y_i(t_1),\ldots ,y_i(t_T))^{\top }$ is the vector of the rates or gradients for species *i*. To ensure non-negative Michaelis–Menten parameters, truncated Normal prior distributions are used:

10 $$\begin{aligned} {\mathbf{K}}_i \sim \mathscr {N}_{\left\{ {\mathbf{K}}_i\ge 0 \right\} }({\mathbf{1}},\nu {\mathbf{I}}) \end{aligned}$$

where ${\mathbf{1}}$ is a vector of ones, ${\mathbf{I}}$ is the identity matrix, $\nu >0$ is a hyperparameter, and the subscript, $\left\{ {\mathbf{K}}_i\ge 0 \right\}$, indicates the truncation condition, i.e. that each element of ${\mathbf{K}}_i$ has to be non-negative. In the original CheMA model (Oates et al. 2014) a truncated g-prior is imposed on the maximum reaction rate vectors ${\mathbf{V}}_i$:

11 $$\begin{aligned} {\mathbf{V}}_i|\sigma ^2_i,{\mathbf{K}}_i \sim \mathscr {N}_{\left\{ {\mathbf{V}}_i\ge 0\right\} }\left( {\mathbf{1}}, T \sigma _i^2 ({\mathbf{D}}_i^\mathsf{T} {\mathbf{D}}_i)^{-1} \right) \end{aligned}$$

where ${\mathbf{D}}_i={\mathbf{D}}_i({\mathbf{K}}_i)$, and a Jeffrey prior is used for the noise variance: $p(\sigma _i^2)\propto \sigma _i^{-2}$. In Sect. 6.2 we demonstrate an intrinsic shortcoming of the g-prior, and we show that the model can be significantly improved by employing a truncated ridge regression prior instead:

12 $$\begin{aligned} {\mathbf{V}}_i|\sigma ^2_i,\delta ^2_i \sim \mathscr {N}_{\left\{ {\mathbf{V}}_i\ge 0\right\} }\left( {\mathbf{1}}, \delta ^2_i \sigma _i^2 {\mathbf{I}} \right) \end{aligned}$$

where $\delta ^2_i$ is a new hyperparameter which regulates the prior strength. For $\sigma _i^2$ and $\delta ^2_i$ we use inverse Gamma priors, $\sigma _i^{2}\sim IG(a_{\sigma },b_{\sigma })$ and $\delta ^2_i \sim IG(a_{\delta },b_{\delta })$. Graphical model representations for both models CheMA and iCheMA are provided in Fig. 3. For inference of the iCheMA model the MCMC sampling scheme in Oates et al. (2014) has to be modified. The new full conditional distributions can be derived from the equations in Sect. 3.2 of Marin and Robert (2007). The details are given in Sect. 3.1, and pseudo-code of the MCMC algorithm is provided in Table 1. Table 2 shows that the replacement of Eq. (11) by Eq. (12) yields a substantial reduction of the computational costs of the MCMC scheme. Figure 6 in Sect. 6.2 shows that this replacement can also lead to a significantly improved network reconstruction accuracy.

**Table 1:**
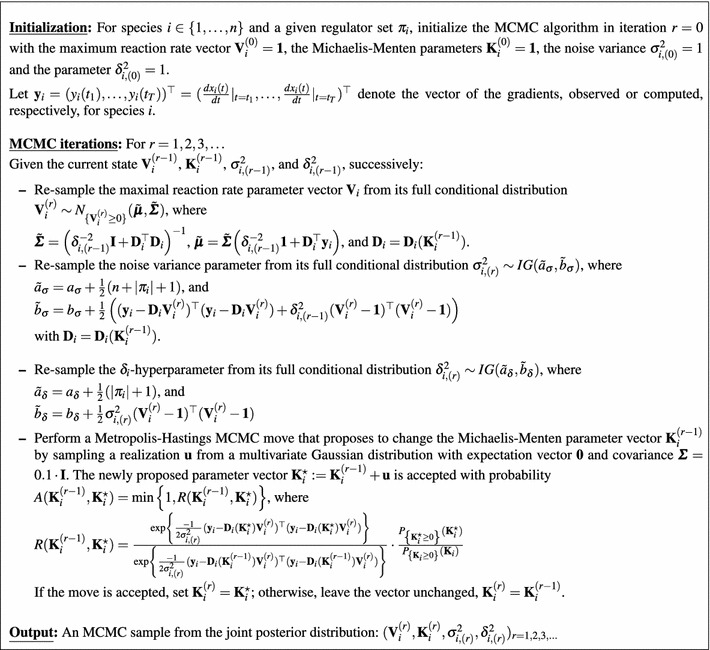
Pseudo Code: MCMC sampling scheme for the iCheMA model

**Table 2:** Computational costs for CheMA and iCheMA

Dimensions	1	2	3	4
CheMA (s)	5.81	13.39	47.57	585.43
iCheMA (s)	7.61	7.93	9.62	13.39

The runtimes are given in seconds for 1000 MCMC iterations (the effective sample sizes for the two methods were not significantly different). The increase of the computational costs for CheMA when increasing the dimension of ${\mathbf{V}}_i$ is discussed in Appendix 1. Both methods were implemented in Matlab, and the MCMC simulations were run on an Intel(R) Core(TM) E6850 with 3GHz

## Inference

### Posterior inference

We refer to the proposed new variant of the CheMA model, which employs an analytical rather than a numerical gradient and replaces the truncated g-prior in Eq. (11) by the truncated ridge regression prior in Eq. (12), as the improved CheMA (iCheMA) model. For iCheMA, as outlined in Sect. 2.3, the Metropolis-within-Gibbs Markov Chain Monte Carlo (MCMC) sampling scheme proposed by Oates et al. (2014) has to be modified. In the new variant (iCheMA) we replace the truncated g-prior on ${\mathbf{V }}_i$ by the truncated ridge regression prior, we use a conjugate inverse Gamma prior rather than a Jeffrey’s prior for the noise variance $\sigma _i^2$, and we introduce a new hyperparameter $\delta _i^2$. We thus have to revise the sampling steps of the original MCMC inference algorithm. We also replace the *approximate* collapsed Gibbs sampling step for the noise variance $\sigma _i^2$ from Oates et al. (2014) by an *exact* uncollapsed Gibbs sampling step. For computing the posterior distribution of the noise variance $\sigma _i^2$,

13 $$\begin{aligned} p\left( \sigma _i^2|{\mathbf{K}}_i,{\mathbf{y}}_i\right) \propto p\left( {\mathbf{y}}_i|\sigma _i^2,{\mathbf{K}}_i\right) p\left( \sigma _i^2\right) \end{aligned}$$

Oates et al. (2014) approximate the marginalization integral

14 $$\begin{aligned} p\left( {\mathbf{y}}_i|\sigma _i^2,{\mathbf{K}}_i\right) = \int p\left( {\mathbf{y}}_i|{\mathbf{V}}_i,\sigma _i^2,{\mathbf{K}}_i\right) p\left( {\mathbf{V}}_i|\sigma _i^2,{\mathbf{K}}_i\right) d{\mathbf{V}}_i \end{aligned}$$

with the closed form solution from Marin and Robert (2007), Chapter 3. This is exact if there are no restrictions on the integration bounds. However, given the underlying positivity constraint for ${\mathbf{V}}_i$, symbolically $\{{\mathbf{V}}_i\ge 0\}$, the integral is no longer analytically tractable and the expressions for Eqs. (13, 14) used in Oates et al. (2014) become an approximation.^4^ We therefore switch to an uncollapsed Gibbs sampling step, where $\sigma _i^2$ is sampled from the full conditional distribution $p(\sigma _i^2|{\mathbf{K}}_i,{\mathbf{V}}_i,{\mathbf{y}}_i)$ and the marginalization integral from Eq. (14) becomes obsolete.^5^

For species *i* and a given regulator set $\pi _i$ we have to sample the maximum reaction rate vector ${\mathbf{V }}_i$, the Michaelis–Menten parameter vector ${\mathbf{K}}_i$, the noise variance $\sigma _{i}^{2}$, and the new hyperparameter $\delta _{i}^{2}$ from the posterior distribution:

15 $$\begin{aligned}&p\big ({\mathbf{V }}_i,{\mathbf{K}}_i,\sigma _{i}^{2},\delta _{i}^{2}|{\mathbf{y}}_i\big ) \propto \nonumber \\&\quad p\big ({\mathbf{y}}_i|{\mathbf{K}}_i,{\mathbf{V}}_i,\sigma _i^2\big ) p\big ({\mathbf{V}}_i|\sigma _i^2,\delta _i^2\big ) p\big (\delta _i^2\big ) p\big ({\mathbf{K}}_i\big ) p\big (\sigma _i^2\big ) \end{aligned}$$

where ${\mathbf{y}}_i:=(y_i(t_1),\ldots ,y_i(t_T))^{\top }$ is the vector of rates or gradients. For the full conditional distribution of ${\mathbf{V}}_i$ we get:

16 $$\begin{aligned} p\big ({\mathbf{V }}_i|{\mathbf{K}}_i,\sigma _{i}^{2},\delta _{i}^{2},{\mathbf{y}}_i\big ) \propto p\big ({\mathbf{y}}_i|{\mathbf{K}}_i,{\mathbf{V}}_i,\sigma _i^2\big ) p\big ({\mathbf{V}}_i|\delta _i^2,\sigma _i^2\big ) \end{aligned}$$

Since ${\mathbf{K}}_i$, $\sigma _i^2$, and $\delta _i^2$ are fixed in Eq. (16) and the (truncated) Gaussian prior on ${\mathbf{V}}_i$ from Eq. (12) is conjugate to the likelihood in Eq. (9), we obtain:

17 $$\begin{aligned} {\mathbf{V }}_i|{\mathbf{K}}_i,\sigma _{i}^{2},\delta _{i}^{2},{\mathbf{y}}_i \sim N_{\{{\mathbf{V }}_i\ge 0\}}\big (\tilde{\varvec{\mu }},\tilde{\varvec{\varSigma }}\big ) \end{aligned}$$

where $\tilde{\varvec{\varSigma }} = \delta _{i}^{2} ({\mathbf{I}} + \delta _{i}^{2} {\mathbf{D}}_i^{\top } {\mathbf{D}}_i)^{-1}$, $\tilde{\varvec{\mu }} = \tilde{\varvec{\varSigma }} (\delta _{i}^{-2} {\mathbf{1}} + {\mathbf{D}}_i^{\top } {\mathbf{y}}_i )$, and ${\mathbf{D}}_i = {\mathbf{D}}_i({\mathbf{K}}_i)$ is the design matrix, built from the rows given in Eq. (8). For the full conditional distribution of $\delta _i^2$ we have:

18 $$\begin{aligned} p\big (\delta _{i}^{2}|{\mathbf{V }}_i,{\mathbf{K}}_i,\sigma _{i}^{2},{\mathbf{y}}_i\big ) \propto p\big ({\mathbf{V}}_i|\sigma _i^2,\delta _i^2\big ) p\big (\delta _i^2\big ) \end{aligned}$$

As ${\mathbf{V}}_i$ and $\sigma _i^2$ are fixed in Eq. (18) and the inverse Gamma prior on $\delta _i^2$ is conjugate for $p({\mathbf{V}}_i|\sigma _i^2,\delta _i^2)$, defined in Eq. (12), we obtain:

19 $$\begin{aligned} \delta _{i}^{2}|{\mathbf{V }}_i,{\mathbf{K}}_i,\sigma _{i}^{2},{\mathbf{y}}_i \sim IG\big (\tilde{a}_{\delta },\tilde{b}_{\delta }\big ) \end{aligned}$$

with $\tilde{b}_{\delta } = b_{\delta } + \frac{1}{2} \sigma _{i}^{2} ({\mathbf{V }}_i - {\mathbf{1}})^{\top } ({\mathbf{V }}_i - {\mathbf{1}})$, and $\tilde{a}_{\delta } = a_{\delta } + \frac{1}{2} ((|\pi _i|+1)$. For the full conditional distribution of $\sigma _i^2$ we have:

20 $$\begin{aligned} p\big (\sigma _{i}^{2}|{\mathbf{K}}_i{\mathbf{V }}_i,\delta _{i}^{2},{\mathbf{y}}_i\big ) \propto p\big ({\mathbf{y}}_i|{\mathbf{K}}_i,{\mathbf{V}}_i,\sigma _i^2\big ) p\big ({\mathbf{V}}_i|\sigma _i^2,\delta _i^2\big ) p\big (\sigma _i^2\big ) \end{aligned}$$

As ${\mathbf{K}}_i$, ${\mathbf{V}}_i$, and $\delta _i^2$ are fixed in Eq. (20) and the Gaussian–Inverse–Gamma prior on $({\mathbf{V }}_i,\sigma _i^2)$ is conjugate for the likelihood in Eq. (9), we get:

21 $$\begin{aligned} \sigma _{i}^{2}\sim IG\big (\tilde{a}_{\sigma },\tilde{b}_{\sigma }\big ) \end{aligned}$$

where $\tilde{b}_{\sigma } = b_{\sigma } + \frac{1}{2} [ ( {\mathbf{y}}_i - {\mathbf{D}}_i{\mathbf{V }}_i)^{\top } ({\mathbf{y}}_i - {\mathbf{D}}_i{\mathbf{V }}_i) + \delta _{i}^{2} ({\mathbf{V }}_i - {\mathbf{1}})^{\top } ({\mathbf{V }}_i - {\mathbf{1}})]$, and $\tilde{a}_{\sigma } = a_{\sigma } + \frac{1}{2} (T + |\pi _i|+1)$. For the mathematical details see, e.g., Chapter 3 of Marin and Robert (2007).

The full conditional distribution of ${\mathbf{K}}_i$ cannot be computed in closed-form so that the Michaelis–Menten parameters have to be sampled by Metropolis–Hastings (MH) MCMC steps. Given the current vector ${\mathbf{K}}_i$, a realization ${\mathbf{u}}$ from a multivariate Gaussian distribution with expectation vector ${\mathbf{0}}$ and covariance matrix ${\varvec{\varSigma }}=0.1 \cdot {\mathbf{I}}$ is sampled, and we propose the new parameter vector ${\mathbf{K}}_i^{\star }={\mathbf{K}}_i+{\mathbf{u}}$ subject to a reflection of negative values into the positive domain. The MH acceptance probability for the new vector ${\mathbf{K}}_i^{\star }$ is then $A({\mathbf{K}}_i,{\mathbf{K}}_i^{\star })=\min \left\{ 1,R({\mathbf{K}}_i,{\mathbf{K}}_i^{\star })\right\}$, with

22 $$\begin{aligned} R\big ({\mathbf{K}}_i,{\mathbf{K}}_i^{\star }\big )= & {} \frac{\exp \left\{ \frac{-1}{2\sigma ^2_{i}} \big ({\mathbf{y}}_i - {\mathbf{D}}_i\big ({\mathbf{K}}_i^{\star }\big ) {\mathbf{V}}_i\big )^{\top } \big ({\mathbf{y}}_i - {\mathbf{D}}_i\big ({\mathbf{K}}_i^{\star }\big ) {\mathbf{V}}_i\big ) \right\} }{\exp \left\{ \frac{-1}{2\sigma ^2_{i}} ({\mathbf{y}}_i - {\mathbf{D}}_i({\mathbf{K}}_i) {\mathbf{V}}_i)^{\top } ({\mathbf{y}}_i - {\mathbf{D}}_i({\mathbf{K}}_i) {\mathbf{V}}_i) \right\} }\nonumber \\&\cdot PR \cdot HR \end{aligned}$$

where the Hastings-Ratio (HR) is equal to one, and the prior probability ratio (PR) depends on the model variant.^6^ For the original CheMA model (Oates et al. 2014) we obtain from Eq. (11):

23 $$\begin{aligned} { PR}_{ CheMA} = \frac{P_{\left\{ {\mathbf{V}}_i\ge 0\right\} }\big ({\mathbf{V }}_i|\sigma _i^2,{\mathbf{K}}_i^{\star }\big )}{P_{\left\{ {\mathbf{V}}_i\ge 0\right\} }\big ({\mathbf{V }}_i|\sigma _i^2,{\mathbf{K}}_i\big )} \frac{P_{\left\{ {\mathbf{K}}^{\star }_i\ge 0\right\} }\big ({\mathbf{K}}_i^{\star }\big )}{P_{\left\{ {\mathbf{K}}_i\ge 0\right\} }\big ({\mathbf{K}}_i\big )} \end{aligned}$$

For the proposed new variant (iCheMA) we get from Eq. (12):

24 $$\begin{aligned} { PR}_{ iCheMA} = \frac{P_{\left\{ {\mathbf{K}}_i^{\star }\ge 0\right\} }({\mathbf{K}}_i^{\star })}{P_{\left\{ {\mathbf{K}}_i\ge 0\right\} }({\mathbf{K}}_i)} \end{aligned}$$

If the move is accepted, we replace ${\mathbf{K}}_i$ by ${\mathbf{K}}_i^{\star }$, or otherwise we leave ${\mathbf{K }}_i$ unchanged. Pseudo code of the MCMC sampling scheme for the new model variant (iCheMA) is provided in Table 1. Table 2 shows that the replacement of Eq. (23) by Eq. (24) yields a substantial reduction of the computational costs of the MCMC inference.

### Model selection

The ultimate objective of inference is model selection, i.e. to infer the *n* regulator sets $\pi _i$ ($i=1,\ldots ,n$) of the interaction processes described by Eq. (6). We compare and critically assess five alternative paradigms: the divergence information criterion (**DIC**), proposed by Spiegelhalter et al. (2002), the ’widely applicable information criterion’ (**WAIC**), proposed by Watanabe (2010), the ’widely applicable Bayesian information criterion’ (**WBIC**), proposed by Watanabe (2013), the cross-validation information criterion (**CVIC**) , proposed by Gelfand et al. (1992), and the **marginal likelihood** (also called ’model evidence’). For the ’marginal likelihood’ paradigm, we compare two numerical methods: Chib’s method (**Chib**), proposed by Chib and Jeliazkov (2001), and thermodynamic integration (**TI**), proposed by Friel and Pettitt (2008). For the latter, we have further assessed the numerical stabilization of the numerical integration proposed by Friel et al. (2013) in a simulation study, which can be found in Appendix 3.

### Posterior probabilities of interactions

For the improved variant of the CheMA model (iCheMA) we follow Oates et al. (2014) and perform ’model averaging’ to compute the marginal posterior probabilities of all regulator-regulatee interactions (i.e. the ’edges’ in the interaction graph). The marginal posterior probability for species *u* being a regulator of *i* is given by:

25 $$\begin{aligned} p(u \rightarrow i|D) \; = \; \frac{\sum _{\pi _i^{\diamond }\in \varPi ^{(u\rightarrow i)}} p(D|\pi _i^{\diamond })p(\pi _i^{\diamond })}{\sum _{\pi _i^{\diamond }\in \varPi } p(D|\pi _i^{\diamond })p(\pi _i^{\diamond })} \end{aligned}$$

where $\varPi$ is the set of all possible regulator sets $\pi _i$ of species *i*, and $\varPi ^{(u\rightarrow i)}$ is the set of all regulator sets $\pi _i$ of *i* that contain the regulator *u*. For simplicity, we chose a uniform prior for $\pi _i$ subject to a maximum cardinality of 3 for the set of regulators (’parents’) of a node.

### Network inference scoring scheme

For the CheMA model (Oates et al. (2014)) and the novel model variant (iCheMA) the marginal interaction posterior probabilities in Eq. (25) can be used to rank the network interactions in descending order. If the true regulatory network is known, this ranking defines the receiver operating characteristic (ROC) curve (Hanley and McNeil 1982), where for all possible threshold values, the sensitivity (or recall) is plotted against the complementary specificity. By numerical integration we then obtain the area under the curve (AUROC) as a global measure of network reconstruction accuracy, where larger values indicate a better performance, starting from AUROC = 0.5 to indicate random expectation, to AUROC = 1 for perfect network reconstruction. A second well established measure that is closely related to the AUROC score is the area under the precision recall curve (AUPREC), which is the area enclosed by the curve defined by the precision plotted against the recall (Davis and Goadrich 2006). AUPREC scores have the advantage over AUROC scores that the influence of large quantities in false positives can be identified better through the precision. These two scores (AUROC and AUPREC) are widely applied in the systems biology community to score the global network reconstructions accuracy (Marbach et al. 2012).

### Causal sufficiency

In Appendix 1 we discuss causal sufficiency and how its violation affects the inference of regulatory network structures.

**Fig. 4:**
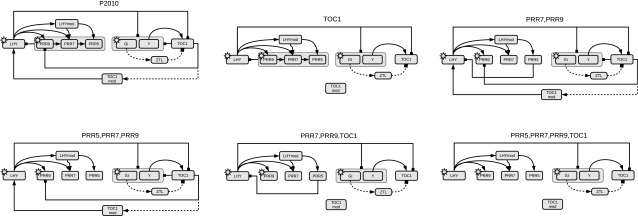
Hypothetical gene regulatory network of the circadian clock in *A. thaliana*, based on Pokhilko et al. (2010). The network displayed in the top left panel is the P2010 network proposed by Pokhilko et al. (2010). The other networks are pruned versions of that network, corresponding to certain protein knock-downs, as noted in each network title. These networks are used for the realistic data simulations, described in Sect. 5.2. *Grey boxes* group sets of regulators or regulated components. *Arrows* symbolize activations and *bars* inhibitions. *Solid lines* show protein–gene interactions; *dashed lines* show protein interactions; and the regulatory influence of light is symbolized by a *sun symbol*. Figure reproduced from Aderhold et al. (2014)

## Evaluation

### ANOVA

Like other network reconstruction models, CheMA and the novel iCheMA model yield a ranking of the regulatory interactions. Hence, if the true interaction network is known, AUROC and AUPREC scores can be computed, as explained in Sects. 3.3 and 3.4. For our performance evaluation on realistic network data, described in Sect. 5.2, we were running simulations for different settings, e.g. related to different regulatory network structures, shown in Fig. 4, and different inference methods. In order to distinguish the relevant effects from the confounding factors, we adopted the DELVE evaluation procedure for comparative assessment of classification and regression methods in Machine Learning (Rasmussen 1996; Rasmussen et al. 1996). That is, we set up a multi-way analysis of variance (ANOVA) scheme to disentangle the factors of interest. For instance, if there are three main effects (*A*, *B*, and *C*), and $y_{ijkl}$ is the *l*-th AUROC (or AUPREC) score obtained for the constellation $A=i$, $B=j$ and $C=k$, then we set up a 3-way ANOVA scheme:

26 $$\begin{aligned} y_{ijkl} = A_i + B_j + C_k + \varepsilon _{ijkl} \end{aligned}$$

where $\varepsilon _{ijkl}{\sim }N(0,\sigma ^2)$ is zero-mean white additive Gaussian noise. We then computed ANOVA confidence intervals, e.g. for the effects of $A=1$, $A=2,\ldots$ on the AUROC scores (i.e. confidence intervals for the parameters $A_1, A_2,\ldots$); see, e.g., Brandt (1999) for details.

Thereby the main effects of the ANOVA models varied in dependence on the addressed research question. Throughout Sect. 6 we use the following five main effect symbols:

- ${\mathbf{N_n}}$ ($n=1,\ldots ,6$) is the effect of the **Network** structure, see Fig. 4 for the six structures,
- ${\mathbf{K_k}}$ ($k=1,\ldots 4$) is the effect of the GP **Kernel**, used for the computation of the analytical gradient (’RBF’, ’PER’, ’MAT32’, or ’MAT52’),
- ${\mathbf{G_g}}$ ($g=1,2$) is the effect of the **Gradient** type, i.e. numerical (’difference quotient’) *versus* analytical (’GP interpolation’),
- ${\mathbf{M_m}}$ ($m=1,\ldots ,12$) is the effect of the inference **Method**, i.e. the iCheMA model and eleven competing methods, listed in Table 5,
- and ${\mathbf{P_p}}$ ($p=1,2$) is the effect of the **Prior** on ${\mathbf{V}}_i$ (i.e. the g-prior from Eq. (11) *versus* the ridge regression prior from Eq. (12).

In Appendix 2, we test the statistical assumptions of the ANOVA scheme in Eq. (26), and we include additional results related to the improvement of GP regression over numerical differentiation, and the influence of the network topology.

### Simulation details

In our study we have included the four IC: **DIC**, **WAIC**, **CVIC**, and **WBIC**, and we have employed four different numerical methods to approximate the marginal likelihood: Chib’s original method (Chib and Jeliazkov 2001) (**Chib naive**), a stabilized version of Chib’s method (**Chib**), proposed here, thermodynamic integration with the trapezoid rule (**TI**), see Eq. (38), and thermodynamic integration with the numerical correction (**TI-STAB**), see Eq. (41).

The computation of the model selection scores (DIC, WAIC, CVIC, WBIC and the MLL with both Chib’s method and TI) requires MCMC simulations; pseudo code can be obtained from Table 1. We monitored the convergence of the MCMC chains with standard convergence diagnostics based on potential scale reduction factors (Gelman and Rubin 1992). The application of Chib’s method is based on the selection of a particular ’pivot’ parameter vector $\tilde{\varvec{\theta }}$, as described under Eq. (34). Initially, we chose $\tilde{\varvec{\theta }}$ to be the MAP (maximum a posteriori) estimator from the entire MCMC simulation. This was found to lead to numerical instabilities, though, as seen from the right panel of Fig. 8. We found a way to numerically stabilize Chib’s method, which we discuss in Appendix 1. We also found that the transition from **TI** to **TI-STAB** proposed by Friel et al. (2013) can be counter-productive, as seen from Table 4. We have investigated this unexpected effect in more detail in a simulation study in Appendix 3. We also found that the improved variant of CheMA (iCheMA) substantially reduces the computational costs of the MCMC-based inference, as shown in Table 2 and discussed in more detail in Appendix 1.

## Data

### Synthetic data

We generate $T=240$ data points $x_s(t_1),\ldots ,x_s(t_T)$ for $n=4$ species ($s=1,\ldots ,4$) from iid standard Gaussian distributions. Subsequently, to obtain non-negative concentrations, the observations of each individual species are shifted such that the lowest value is equal to 0, before we follow Oates et al. (2014) and re-scale the observations of each species to mean 1. With $x_1$ taking the role of the degradation process and $x_2$ being an activating regulator ($I_{2,y}=1$) of a target species, whose gradient *y*(.) we here assume to be directly observable, we generate target observations $y(t_j)$ with Eq. (6). We then have for $j=1,\ldots ,T$:

27 $$\begin{aligned} y(t_j) \; =\; -v_{0,y} x_1(t_j) + v_{2,y} \frac{x_2(t_j)}{x_2(t_j) + k_{2,y}} + \epsilon _{t_j} \end{aligned}$$

where ${\mathbf{V}}_y=(v_{0,y},v_{2,y})^\mathsf{T}$ is the vector of maximum reaction rate parameters, ${\mathbf{K}}_y=(k_{2,y})$ contains the Michaelis–Menten parameter(s), and $\epsilon _{t_j} \sim \mathscr {N}(0,\sigma ^2)$ is additive iid Gaussian noise ($j=1,\ldots ,T$). We keep the Michaelis–Menten parameter fixed at $k_{2,y}=1$, while we vary the rates $v_{0,y}$ and $v_{2,y}$, as indicated in Table 3. Our goal is to infer the set of regulators $\varvec{\pi }\!_{y}$ of *y* out of all subsets of $\left\{ x_2,x_3,x_4 \right\}$, where $\varvec{\pi }\!_{y}=\left\{ x_2 \right\}$ is the true regulator set. The effect of the degradation, taken into account by $x_1$, is included in all 8 models.

**Table 3:** Parameter settings for the synthetic data of Sect. 5.1. The parameters $v_{0,y}$ and $v_{2,y}$ are the maximum reaction rates

Parameter	**1**	**2**	**3**	**4**	**5**	**6**	**7**	**8**	**9**
$v_{0,y}$	1	0.5	1.5	2	0.2	2	3	0.2	0.1
$v_{2,y}$	1	1	1	1	1	0.2	0.1	2	2

**Table 4:** Score differences for iCheMA, applied with thermodynamic integration: TI *versus* TI-STAB

Spread-factor (*sf*)	$(\delta ^2=sf, \nu =0.5)$		$(\delta ^2=sf, \nu =sf)$	
	TI	TI-STAB	TI	TI-STAB
0.01	39	33	166	156
0.1	38	33	76	68
1	27	23	27	22
10	8	5	8	6
100	4	5	4	4
10000	5	119	5	119
1e+08	52	3.1e+10	52	1.7e+08
1e+16	52	9.8e+24	53	2.7e+24
1e+20	52	3.2e+32	51	3.3e+34

The table shows the MLL differences between a true and an over-complex model for the synthetic data from Sect. 5.1 for different spread factors *sf*. TI from Eq. (38) and the stabilized variant TI-STAB from Eq. (41) were applied using $K=10$ discretization points and the power $m=8$ in Eq. (42). The setting of the hyperparameters $\delta ^2$ in Eq. (12) and the prior variance $\nu$ in Eq. (10) is shown in the first row of the table. The diffuseness of the corresponding prior distribution(s) increases with the spread factor (*sf*). The score differences for TI-STAB sharply increase for $sf > 100$

### Realistic data

For an objective model evaluation, we use the benchmark data from Aderhold et al. (2014), which contain simulated gene expression and protein concentration time series for ten genes in the circadian clock of *A. thaliana*. The time series correspond to measurements in 2-h intervals over 24 h, and are repeated 11 times, corresponding to different experimental conditions. We use time series generated from six variants of the circadian gene regulatory network in *A. thaliana*, shown in Fig. 4; these variants correspond to different protein, i.e. transcription factor, knock-downs. The molecular interactions in these graphs were modelled as individual discrete events with a Markov jump process, using the mathematical formulation from Guerriero et al. (2012) and practically simulated with Biopepa (Ciocchetta and Hillston 2009), based on the Gillespie algorithm (Gillespie 1977). For large volumes of cells, the concentration time series converge to the solutions of ODEs of the form in Eq. (5). However, for smaller volumes, time series simulated with Markov jump processes contain stochastic fluctuations that mimic the mismatch between the ODE model and genuine molecular processes, and the volume size was chosen as described in Guerriero et al. (2012) so as to match the fluctuations observed in real quantitative reverse transcription polymerase chain reaction (qRT-PCR) profiles. For the network reconstruction task, we only kept the gene expression time series and discarded the protein concentrations; this emulates the common problem of systematically missing values for certain types of molecular species (in our case: protein concentrations).

### Real data

We have applied the iCheMa model to gene expression time series obtained with real-time polymerase chain reaction experiments to predict the circadian regulatory network in *Arabidopsis thaliana*. All details and the results can be found in Appendix 4.

**Table 5:** State-of-the-art network reconstruction methods

Abbreviation	Full name
HBR	Hierarchical Bayesian regression
Lasso	Sparse regression with L$_1$ penalty
ElasticNet	Sparse regression with L$_1$
	and L$_2$ penalty
Tesla	Sparse regression with time-varying
	change-points
GGM	Graphical Gaussian models
SBR	Sparse Bayesian regression
BSA	Bayesian spline autoregression
SSM	State-space models
GP	Gaussian processes
MBN	Mixture Bayesian networks
BGe	Gaussian Bayesian networks

The table shows a list of the methods that were included in the comparative evaluation study with the realistic network data from Sect. 5.2. A detailed description of these methods can be found in Aderhold et al. (2014), and references therein

## Results

This section discusses the effect of gradient approximation (Sect. 6.1), the influence of the prior (Sect. 6.2), the accuracy of model selection (Sect. 6.3), the relative performance compared to the current state of the art (Sect. 6.4), and the problem of model mismatch (Sect. 6.5).

### Evaluating the effect of the gradient computation

**Fig. 5:**
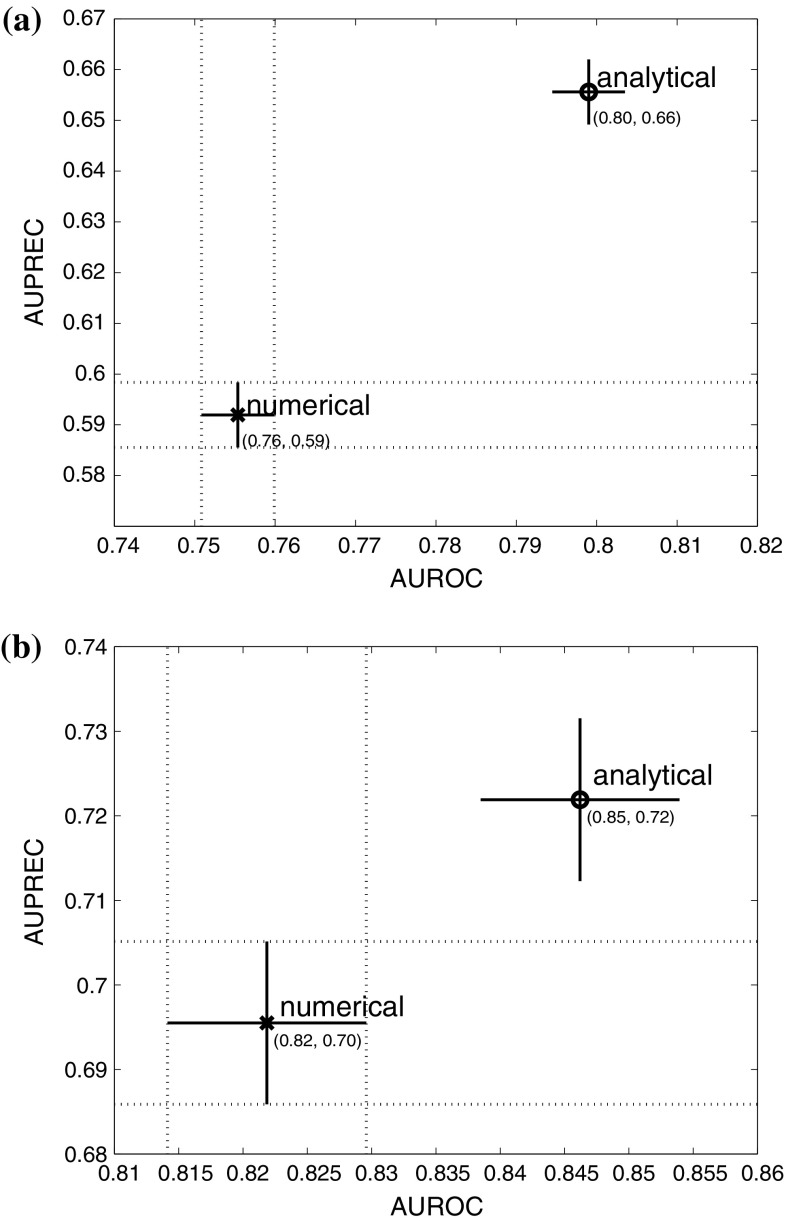
Effect of the gradient type: numerical *versus* analytical. For the realistic network data from Sect. 5.2 we compared the network reconstruction accuracy of a numerical and an analytical gradient. The numerical gradient was computed with the difference quotient, as proposed in Oates et al. (2014), and a time difference of $\delta _t = 2$ h. The analytical gradient, proposed here, was derived from the derivative of a Gaussian process (GP) using the radial basis function kernel with optimized parameters, as implemented in the Matlab library gpstuff. Both panels show the mean AUROC and AUPREC scores with confidence intervals for the effect of the gradient type derived from ANOVA models. In **a** all methods listed in Table 5 and iCheMA were included so that the ANOVA model has 3 effects: **gradient type** $G_g$, network structure $N_n$, and method $M_m$: $y_{gnml} = G_g + N_n + M_m + \varepsilon _{gnml}$, see Sect. 4.1 for details. **b** shows the confidence intervals for iCheMA only, i.e. for an ANOVA model with $M_m$ being removed

To illustrate the difference in the accuracy of network inference between a numerically calculated gradient using the difference quotient defined in Eq. (3) and an analytical gradient using a GP, we applied both gradient types to the realistic data of Sect. 5.2 and evaluated the performance of the methods listed in Table 5 together with iCheMA. The difference quotient was calculated with a time difference of $\delta _t = 2$ h^7^, and the analytical gradient was calculated with a GP using a RBF kernel as described in Sect. 2.2. The results of a preliminary study, in which we investigated the effect of the GP kernel on the network reconstruction accuracy, can be found in Appendix 2. We have recorded the AUROC and AUPREC scores for all the different conditions mentioned in Sect. 5.2, and summarize the outcome with an ANOVA analysis that treats the different conditions and methods as distinct effects. Fig. 5a shows that the results for the data with the analytical rate estimation significantly improves the performance over the numerical difference quotient while taking all methods of Table 5 and iCheMA into account. The same trend can be observed in Fig. 5b, which only considers the results for iCheMA. Hence, we conclude that network reconstruction accuracy significantly improves when an analytically derived gradient using a GP is used instead of the numerical difference quotient used in Oates et al. (2014). This can be observed for both a broad variety of different network reconstruction methods as well as specifically for the iCheMA method.

### Evaluating the influence of the parameter prior

**Fig. 6:**
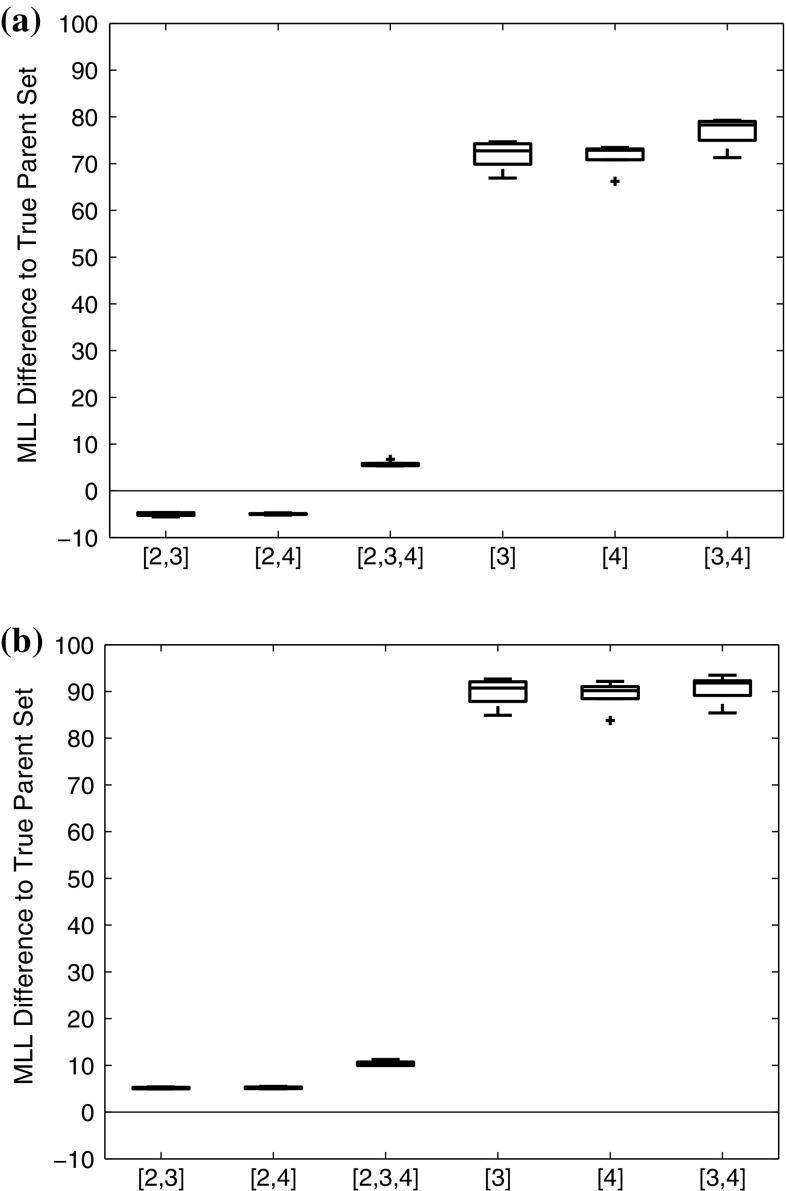
Comparison of CheMA and iCheMA: effect of the prior. The plots show the differences in the log marginal likelihoods (MLL) between the true regulator–regulatee model and six alternative wrong models, obtained with CheMA/iCheMA for the data from Sect. 5.1. There are three potential regulators $\{x_2,x_3,x_4\}$ in the system, with $\varvec{\pi }\!_{y}=\{x_2\}$ (’[2]’) being the true regulator of the response *y*. The configurations on the *horizontal axis* define alternative regulator configurations. The log score differences have been averaged across the nine parameter configurations, shown in Table 3, and four noise settings ($\sigma ^2= 0.05,0.1,0.2,0.4$). The box plots show the distributions of the average log score differences for 10 independent data instantiations. Positive values indicate that the true model was identified correctly; for negative differences, the wrong model had a higher score and would thus be erroneously selected. The results for the g-prior (CheMA) from Eq. (11) are shown in (**a**); the results for the ridge regression prior (iCheMA) from Eq. (12) are shown in (**b**)

**Fig. 7:**
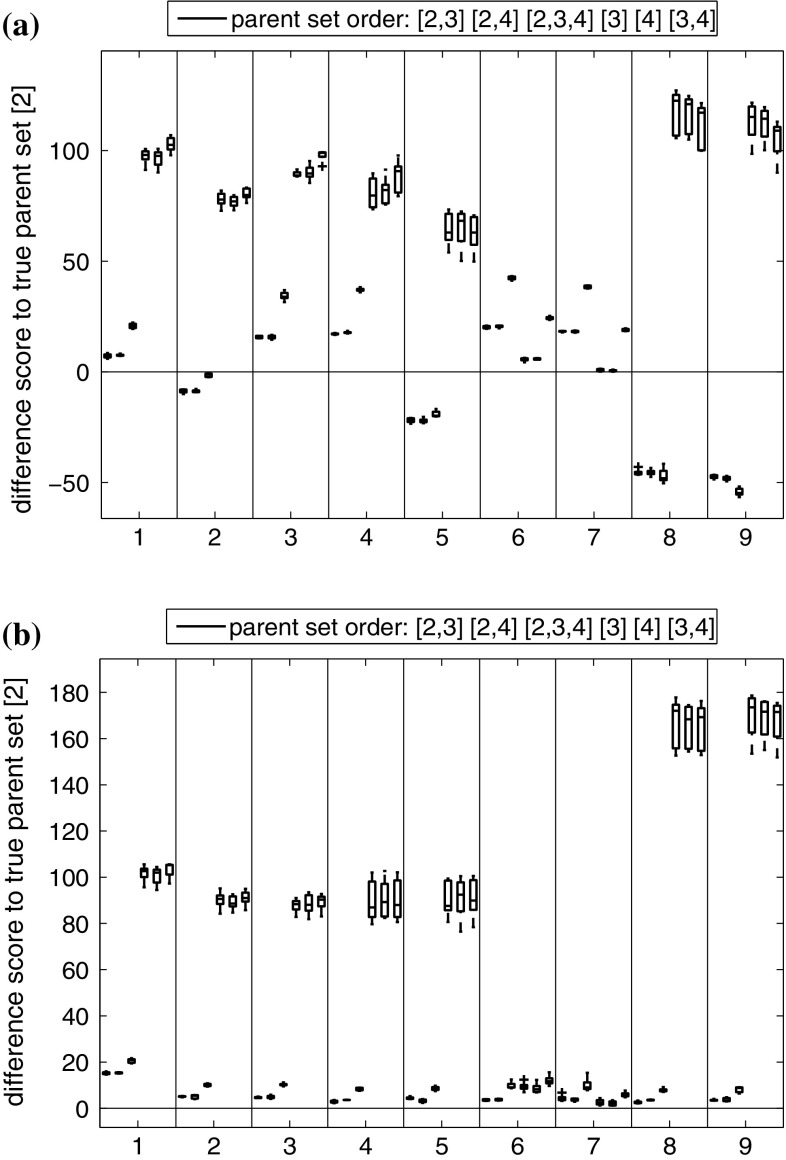
Detailed Chib log marginal likelihood (MLL) scores for the original CheMA method with g-Prior (**a**) and a modified method with a ridge prior (**b**). Detailed plots from which the average score distributions in Fig. 6 are derived. Each of the nine slots along the *horizontal axis* corresponds to one parameter configuration of $(v_{0,y},k_{2,y},v_{2,y})$ as displayed in Table 3 and used for the synthetic data in Sect. 5.1. The slots contain the Chib MLL difference scores between a wrong parent configurations (the parent configurations are aligned from left to right in each slot according to the order in the legend) and the true parent set ([2]). Positive values indicate that the true parent configuration receives a higher score

To evaluate the influence of the parameter prior on model selection, we computed the MLL for the g-prior in Eq. (11) and the ridge regression prior in Eq. (12) for the synthetic data from Sect. 5.1. For each of the 9 parameter settings, shown in Table 3, and 4 different noise variances $\sigma ^2$ we generated 10 independent data instantiations from Eq. (27). We then applied the stabilized Chib method (**Chib**) for approximating the MLLs for all possible regulator–regulatee configurations, and computed the logarithmic Bayes factors (i.e. the differences of the MLLs) between the true and each wrong model. Fig. 6 gives the average logarithmic Bayes factors (averaged across the $9\times 4=36$ parameter settings) for 10 independent replications. In Fig. 6 the distributions of the MLL score differences are represented with boxplots, where positive values indicate that the true structure is correctly selected, whereas negative values indicate that the wrong alternative structure is erroneously selected. It can be seen that regulator sets that do not contain the true regulator (corresponding to the three rightmost box plots) are clearly rejected. However, for the over-complex alternative models (three leftmost box plots), which contain spurious regulators, the MLL score difference obtained with the g-prior fails to consistently favour the true model. Two out of three differences are negative in Fig. 6a, indicating that an over-complex model is preferred to the true one. For the proposed ridge regression prior, on the other hand, the MLL score difference does succeed in consistently favouring the true structure, as displayed in Fig. 6b. We had a closer look at the results for the individual parameter sets, shown in Fig. 7. This figure reveals that the g-prior performed well for some parameter settings, but not for others. In particular, we found that the g-prior systematically fails when ’$v_{0,y}<1\le v_{2,y}$’ (see Table 3). In Appendix 1 we provide a theoretical explanation for this trend.

### Model selection

**Fig. 8:**
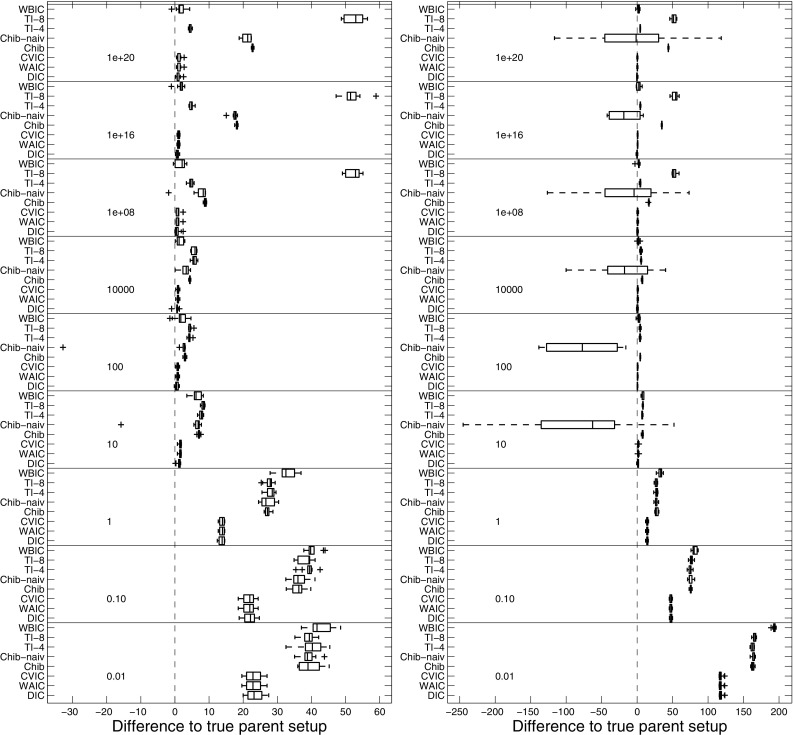
Score difference between the true and an over complex regulator-regulatee configuration for iCheMA, applied with different model selection schemes. Each box plot includes the average results from 10 independent data instantiations for the model of Sect. 5.1. The over-complex configuration includes a spurious regulator. Positive values indicate that the true model is favoured over the over-complex model. **DIC**, **WAIC**, **CVIC**, and **WBIC** are the IC described in Appendix 1; the methods for calculating the MLL are: a naive implementation of Chib (**Chib-naiv**), a stabilized version of Chib’s method (**Chib**), and thermodynamic integration in two variants (**TI-4** and **TI-8**). Both TI-variants were applied with $K=10$ discretization points and differ w.r.t. the power $m\in \{4,8\}$ in Eq. (42). The panels demarcated by horizontal lines correspond to different prior distributions of the interaction parameters, characterized by the spread factor *sf*, ranging from 0.01 to 1e+20. *Left panel* The prior variance of the Michaelis–Menten parameters in Eq. (10) was kept fixed at $\nu =0.5$. The spread hyperparameter $\delta ^2$ for the prior of the reaction rate parameters in Eq. (12) was varied, i.e. $\delta ^2=sf$. *Right panel* Both $\delta ^2$ and $\nu$ take the value *sf*, i.e. $\delta ^2=\nu =sf$. A higher resolution for the IC (plotted on a different scale) is available from Fig. 9

We used the synthetic data from Sect. 5.1 to cross-compare the performance of the model selection schemes; for an overview see Appendix 1. The MLL based selection procedures score the individual models with respect to the differences in the MLL (or log Bayes factors). We compare these results with the score differences of the various IC. For inference we used the novel iCheMA model, and we varied the hyperparameters of the prior distributions so as to obtain increasingly diffuse prior distributions. In a first scenario we set the hyperparameter $\delta ^2$ in Eq. (12) to a value *sf*, which we refer to as ’spread-factor’, and we kept $\nu =0.5$ in Eq. (10) fixed. In the second scenario we set both hyperparameters $\delta ^2$ and $\nu$ to the spread factor *sf*. Figure 8 shows boxplots of the log score differences between the true model and an over-complex alternative model (with one redundant regulator-variable) for both scenarios and increasing spread factors (*sf*). Again positive differences indicate that the true structure is correctly selected, whereas negative differences indicate that the alternative over-complex structure is erroneously selected. The overall trend revealed in Fig. 8 is that the log score difference decreases with the spread-factor (i.e. with the diffuseness of the prior) for most of the model selection criteria.

**Fig. 9:**
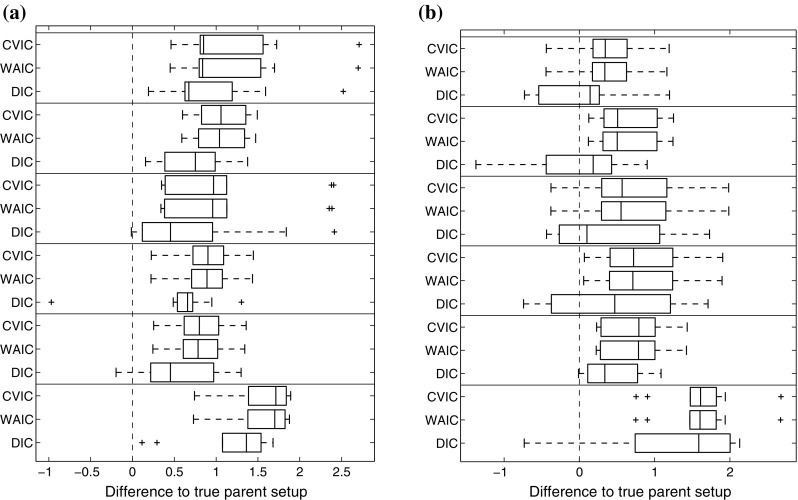
Score differences for iCheMA, for different information criteria. This figure replicates the results from Fig. 8 for the information criteria DIC, WAIC and CVIC, on a different scale for improved resolution, for spread factors (*sf*) ranging from 10 to $1e+20$. **a** is extracted from the left panel of Fig. 8, and **b** is extract from right panel of Fig. 8. Negative score differences indicate that the over-complex model is favoured over the true one

The parameter priors in Eqs. (10) and (12) are Gaussians centred on $\mu =1$, with different variances. For low spread-factors *sf* (i.e. for low prior variances), both groups of criteria (IC and MLL) clearly favour the true model, since the prior ’pulls’ the spurious interaction parameter from its true value of zero towards a wrong value of $\mu =1$. As the prior becomes more diffuse, the score differences become less pronounced, but still select the true model up to spread factors of about $sf\approx 100$. As the prior becomes more diffuse, with the spread factor exceeding $sf> 100$, the IC occasionally fail to select the correct model. A more detailed representation focusing on the IC and larger spread factors is given in Fig. 9. It is seen that among the IC it is mainly DIC that repeatedly fails to select the true model (the central inter-quartile range of the score difference distribution, between the first and third quartile, includes negative values), whereas for the other information criteria the selection of the wrong model is relatively unlikely (the central inter-quartile range does not include negative values). Two of the four MLL methods, namely **TI-8** and **Chib**, start to increasingly favour the true model as the spread factor further increases beyond $sf> 1000$. This is a consequence of Lindley’s paradox, whereby MLL increasingly penalizes the over-complex model for increasingly vague priors. **TI-4**, in principle, shows a very similar trend but the score difference is lower than for **TI-8**, indicating that the choice of the discretization points (i.e. the applied temperature ladder) implied by the power $m\in \{4,8\}$ in Eq. (42) can critically affect the result.

Among the MLL methods, the naive application of Chib’s method, **Chib naive**, as proposed in Chib and Jeliazkov (2001), shows a completely different pattern and systematically fails to select the correct model for large spread factors. A theoretical explanation for this instability is provided in Appendix 1. We achieve a stabilization of Chib’s method, referred to as **Chib**, by selecting the pivot parameter set $\tilde{\varvec{\theta }}$ with the highest posterior probability within the set of actually sampled parameters (excluding the parameter states from the burn-in phase). We refer to Appendix 1 for details.

The left panel of Fig. 8 shows that the different ways of computing the MLL give very similar results up to a prior spread factor of about 1e+08. For spread factors exceeding this value, the results differ. The MLL computed with Chib’s method (**Chib**) monotonically increases, as expected from Lindley’s paradox. The MLL computed with **TI** is obtained without numerical stabilization and reaches a plateau, with different values obtained for different trapezium sum discretization schemes (determined by *m* in Eq. (42)). This is a numerical discretization error that results from the form of the integrand in Eq. (37), which has most of its area concentrated on values near $\tau =0$. We tried to stabilize TI with the corrected trapezium rule, replacing Eq. (38) by Eq. (41). Interestingly, this transition from **TI** to **TI-STAB** turned out to be occasionally counter-productive, as shown in Table 4. We have investigated the effect of the numerical “stabilization” more thoroughly in a simulation study based on a Bayesian linear regression model. We found that TI-STAB can fail for small numbers of discretization points and diffuse priors. The study and its results can be found in Appendix 3. In our subsequent simulations we use the numerically stabilized variant of Chib’s method (**Chib**), which has a 10-fold lower numerical complexity compared to **TI** (because we used 10 different temperatures $\tau$ for TI).

**Fig. 10:**
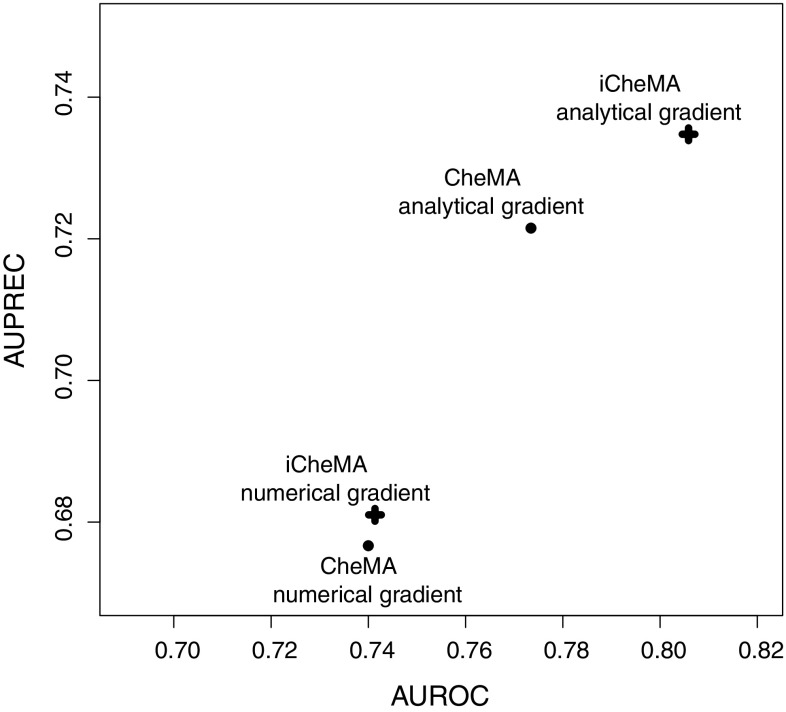
Network reconstruction for the wildtype network. The scatter plot shows the network reconstruction accuracy for the CheMA model and its new variant iCheMA, using realistic data (see Sect. 5.2) generated from the wildtype network in Fig. 4. Both methods, CheMA and iCheMA, were applied with both a numerical and an analytical gradient

### Comparison with state-of-the-art network reconstruction methods

**Fig. 11:**
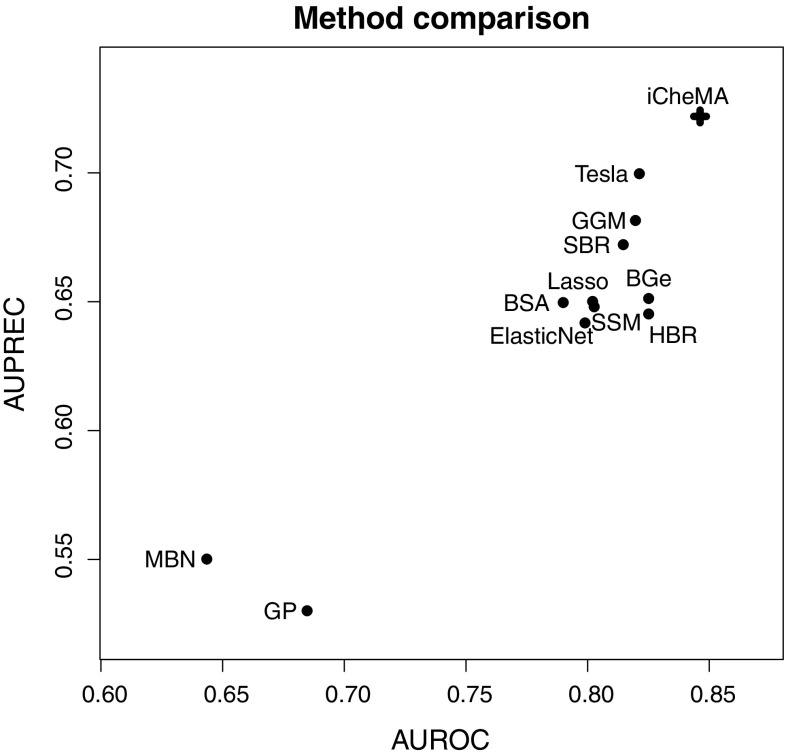
Comparison between the iCheMA model and state-of-the-art network reconstruction methods. The scatter plot shows the network reconstruction accuracy for the novel iCheMA model and the eleven alternative methods from Table 5, using the realistic network data from Sect. 5.2, generated from the six network structures of Fig. 4. All network reconstruction methods used the analytical gradient, obtained from GP regression with an RBF kernel, and the displayed AUROC and AUPREC values are the effects of the method, derived from an ANOVA with 2 effects: **method** $M_m$ and network structure $N_n$: $y_{mnl} = M_m + N_n + \varepsilon _{mnl}$; see Sect. 4.1 for details

We have compared the prediction accuracy of the proposed new novel variant (iCheMA) of the semi-mechanistic CheMA model of Oates et al. (2014) with the original CheMA model and 11 state-of-the-art machine learning methods, assessed in Aderhold et al. (2014). These methods are listed in Table 5 and were applied as described in Aderhold et al. (2014). The network reconstruction accuracy performance was tested on the realistic gene expression profiles from Sect. 5.2, based on the six network structures from Fig. 4. The results in terms of AUROC and AUPREC scores are shown in Fig. 11 and demonstrate that the iCheMA model outperforms all alternative methods. The CheMA model of Oates et al. is not included in this comparison. Due to the substantially higher computational costs, the simulations for all six networks in Fig. 4 would require several weeks of computing time on a medium-size cluster, as indicated by Table 2. In order to keep the computational complexity manageable, we compared CheMA and iCheMA in a separate study, using only data from the wildtype network, proposed by Pokhilko et al. (2010) and shown in the top left panel of Fig. 4. Note that for all methods included in Fig.11, the gradient for the response $\frac{d x_i(t)}{dt}$ of Eq. (6) is derived from an analytical solution of the derivative using a GP with RBF kernel, as described in Sect. 2.2. The original definition of CheMA, on the other hand, uses a numerical gradient estimation. To separate out the effects of gradient estimation and the other differences between the methods, we applied both CheMA and iCheMA with both gradients: the numerical and the analytical gradient. The results are shown in Fig. 10. The two findings of this study are: iCheMA consistently outperforms CheMA, and the analytical gradient leads to a significant improvement in the prediction accuracy over the numerical gradient.

**Fig. 12:**
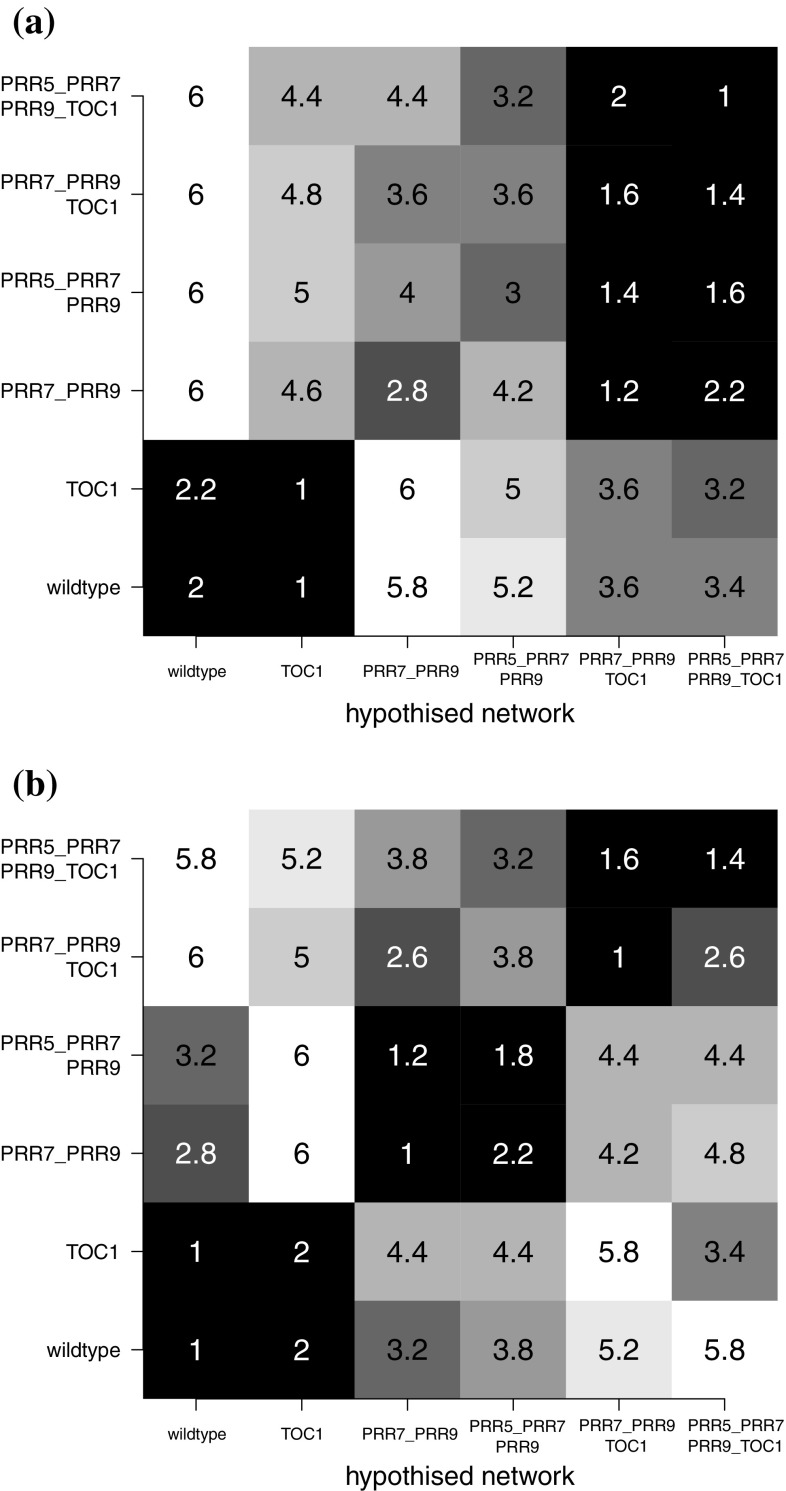
Network structure selection with iCheMA. The figure assesses the network structure identification with iCheMA based on the Biopepa data described in Sect. 5.2. **a** Heatmaps showing the average ranks (averaged over 5 independent data instantiations) of six candidate networks (shown in Fig. 4) based on MLL (computed with Chib’s method). The rows show the true network from which the data were generated. The columns show the candidate networks used for inference. A rank of 1 (black) in the diagonal indicates that the true network is consistently selected. The mathematical model is based on Eq. (6), i.e. Michaelis–Menten kinetics with interactions between regulators modelled as additive effects. **b**: Like **a**, but with multiplicative terms added to the interaction model to allow for protein complex formation

### Model selection for network identification

As a final test, we evaluated the accuracy of model selection for network identification, using the data from Sect. 5.2. These data contain gene expression time series from the six gene regulatory networks of Fig. 4, which contain one wildtype and five mutant networks from protein knock-down experiments. We computed the MLL (with Chib’s method) for each of the candidate networks, for each data set in turn. The evaluation was repeated over five independent data instantiations. For each data set, all models were ranked based on the MLL, and the ranks were averaged over all data sets. This leads to the six-by-six confusion matrix of Fig. 12a, where the rows represent the networks used for data generation, and the columns represent the candidate networks ranked with the MLL. Note that we have emulated the mismatch between data generation and inference characteristic for real applications. For data generation, molecular interactions corresponding to the edges in the network were modelled with complex Markov jump processes, as described in Sect. 5.2, with the intention to mimic real biological processes. For inference and model selection, interactions were modelled with Michaelis–Menten kinetics, corresponding to Eq. (6), and the interactions between different regulators were modelled additively, by adding the Michaelis–Menten terms—this reduction in complexity is required for general computational tractability and scalability. The results are shown in Fig. 12a. The diagonal elements of the matrix show the average ranks for the correct network. Two of the six network structures are consistently correctly identified (average rank 1), but for the other four structures, the average ranks vary between 1.6 and 3. This failure to consistently identify the true network tallies with the fact that the AUROC scores in Fig. 11 are significantly below 1.0. The explanation is that the iCheMA model is currently restricted to additive interactions, as shown in Eq. (6). The data generation process, summarized in Sect. 5.2 and available in complete mathematical description from Guerriero et al. (2012), contains molecular processes related to complex formation (e.g. protein heterodimerization). Complex formation involving two transcription factors *a* and *b* acting on target gene *i* is mathematically described by a product of Michaelis–Menten terms of the form

$$\begin{aligned}&\frac{v_{a,i}\big [I_{a,i} x_a(t^{\star })+(1-I_{a,i}) k_{a,i}\big ]}{x_a(t^{\star })+k_{a,i}} \\&\quad \times \frac{v_{b,i} \big [I_{b,i} x_{b}(t^{\star })+(1-I_{b,i}) k_{b,i}\big ]}{x_{b}(t^{\star })+k_{b,i}} \end{aligned}$$

**Table 6:** Rank difference for Chib’s MLL and different information criteria

	Additive terms	Explicit product terms
	rank diff. (se)	rank diff. (se)
Chib’s MLL	5.4 (0.35)	2.2 (0.18)
DIC	7.0 (0.26)	6.2 (0.32)
WAIC	6.8 (0.37)	1.4 (0.11)
CVIC	6.8 (0.35)	1.6 (0.10)
WBIC	7.8 (0.51)	3.0 (0.13)

The analysis was carried out on the Biopepa data from Sect. 5.2. The numbers show the difference between the actual rank of the true network (a value between 1 and 6) and the optimal rank (1). They are obtained from heatmaps like Fig. 12a, b by deducting 1 from the diagonal elements and adding them together. The resulting values can vary from 0 (perfect match in all networks) to 25 (for each network a model with the most unlikely parent configuration is selected). Lower rank differences indicate a closer match to the true networks. The numbers outside the brackets show the average over all five data instantiations. The values inside the brackets show the standard error (se)

where the symbols have the same meaning as in Eq. (6); see Pokhilko et al. (2010) for explicit mathematical expressions.

We included prior knowledge about molecular complex formation and expanded the iCheMA model accordingly to include the corresponding product of Michaelis–Menten terms in Eq. (6). We then computed the MLL as before and repeated the analysis. The results are shown in Fig. 12b and demonstrate that, by making the model more faithful to the data-generating process, model selection has substantially improved: the average ranks of the true network (shown in the diagonal elements of the matrix) are never worse than a value of 2 (out of 6), and reach the optimal value of 1 in 50 % of the cases. The corresponding results for the various IC are displayed in Table 6. When restricting the model to additive terms (greater mismatch between data and model), MLL outperforms the IC, as presumably expected. Interestingly, when reducing the mismatch between data and model by including the product terms, two IC, WAIC and CVIC, are competitive with MLL and perform even slightly better. DIC is substantially outperformed by MLL and the competitive information criteria WAIC and CVIC; these findings are consistent with the earlier results from Sect. 6.3. The performance of WBIC lies between WAIC /CVIC and DIC.

## Discussion

Automatic inference of regulatory structures in our study is based on a bi-partition of the variables into putative regulators (transcription factor proteins) and regulatees (mRNAs) and a physical model of the regulation processes based on Michaelis–Menten kinetics. This effectively conditions the inference on assumed prior knowledge and is, as such, contingent on the accuracy of these assumptions. In our study we have allowed for a mismatch between the assumed prior knowledge and the ground truth. First, the assumed model is deterministic, defined in terms of ordinary differential equations, while the data-generation mechanism is stochastic (simulated with a Markov jump process). Second, the interaction model is additive, while the data-generation mechanism includes multiplicative terms. Third, we have allowed for the possibility of missing data (missing protein concentrations). Our results show that due to this mismatch, the true causal system cannot be learned (see e.g. Fig. 11, which shows AUROC and AUPREC scores clearly below 1). However, our work suggests that causal inference based on a simplified physical model achieves significantly better results than inference based on an empirical model. (See Fig. 11. Note that only iCheMA is based on a physical model; all the other methods use machine learning methods based on empirical modelling). Our study also quantifies how the performance improves as the physical model is made more realistic; see Fig. 12.

Semi-mechanistic modelling is a topical research area, as evidenced by the recent publication by Babtie et al. (2014). Our article complements this work by addressing a different research question. The objective of Babtie et al. is to investigate how uncertainty about the model structure (i.e. the interaction network defined by the ODEs) impacts on parameter uncertainty, and how parameter confidence or credible intervals are systematically underestimated when not allowing for model uncertainty. Our article addresses questions that have not been investigated by Babtie et al. : how accurate is the network reconstruction or ODE model selection, which factors determine it, and to what extent? Our work has been motivated by Oates et al. (2014), and we have shown that the authors’ seminal work, which won the best paper award at ECCB 2014, can be further improved with two methodological modifications: a different gradient computation, based on GP regression, and a different parameter prior, replacing the g-prior used by Oates et al. by the ridge regression prior more commonly used in machine learning. These two priors have e.g. been discussed in Chapter 3 of Marin and Robert (2007), but without any conclusions about their relative merits. Our study provides empirical evidence for the superiority of the ridge regression prior (Fig. 6) in the context of semi-mechanistic models, and a theoretical explanation for the reason behind it (Sect. 6.2). Table 2 shows that the new iCheMA variant reduces the computational costs drastically; a theoretical explanation for the reduction is provided in Appendix 1.

Our work has led to deeper insight into the strengths and shortcomings of different scoring schemes and numerical procedures. We have investigated the effectiveness of DIC as a method of semi-mechanistic model ranking. DIC is routinely used for model selection in Winbugs (Lunn et al. 2012), and the paper in which it was introduced (Spiegelhalter et al. 2002) has got over 5000 citations at the time of the submission of the present article. However, our findings that in the context of network learning DIC often prefers a model with additional spurious complexity over the true model (Fig. 9) questions its viability as a selection tool for semi-mechanistic models.

We have further compared different methods for computing the MLL. We have shown that Chib’s method (Chib and Jeliazkov 2001) can lead to numerical instabilities. These instabilities have also been reported by Lunn et al. (2012) and are presumably the reason why Chib’s method is *not* available in Winbugs. We have identified the cause of the numerical instability (see Appendix 1), and propose a modified implementation that substantially improves the robustness and practical viability of the method. This modification appears to be even preferable to thermodynamic integration, which at higher computational complexity shows noticeable variation with the discretization of the integral in Eq. (37) and the number of ‘temperatures’. It has been suggested (Friel et al. 2013) that the accuracy of thermodynamic integration can be improved by including second-order terms in the trapezium sum—see Eq. (41)—but the findings of our study are that this correction is no panacea for a general improvement in numerical accuracy, and that there are scenarios where the second-order correction can be counter-productive. It has come to our attention that a more recent method for improving thermodynamic integration has been proposed by Oates et al. (2016). Including this method in our benchmark study would be an interesting project for future research.

Due to the high computational complexity and potential instability of the MLL computation, several articles in the recent computational statistics literature have investigated faster approximate but numerically more stable alternatives. In our work, we have included WAIC, CVIC and WBIC as alternatives to MLL and evaluated their potential for model selection in two benchmark studies. It turns out that these more recent IC significantly outperform DIC (Figs. 8 and 9), and that WAIC and CVIC are compatible in performance with model selection based on the MLL (Table 6). It is advisable that several independent studies for different systems be carried out by independent researchers in the near future, but our study points to the possibility that statistical model selection in complex systems may be feasible at a comparable degree of accuracy but with substantially lower computational costs than with MLL.

## Figures and Tables

**Fig. 13:**
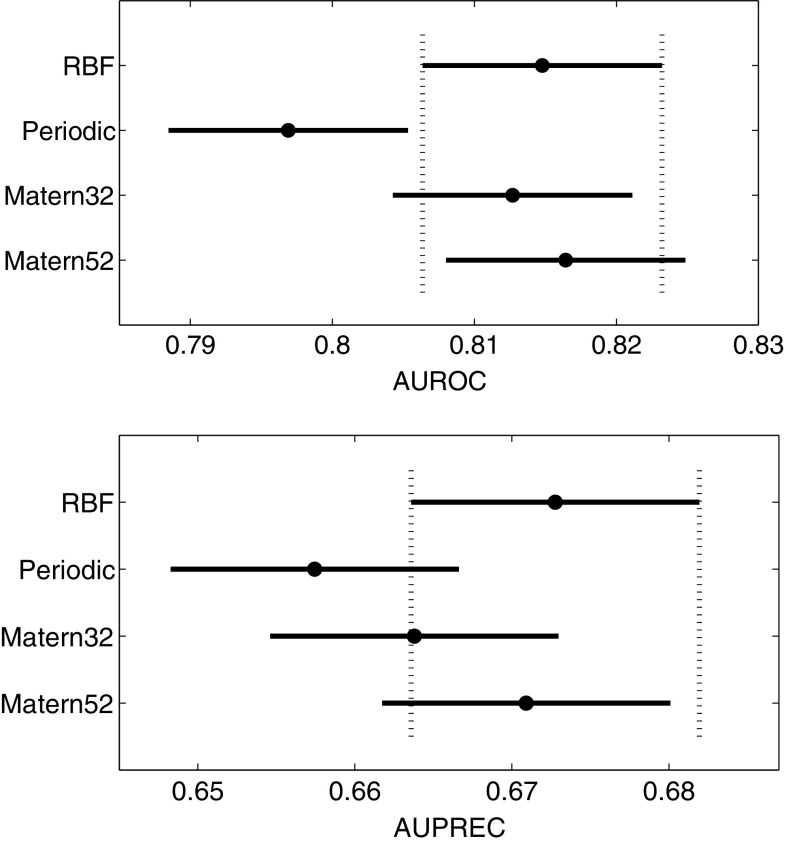
Difference in network reconstruction accuracy for several Gaussian process (GP) kernels. The plots show the effect of four different gradients on the network reconstruction accuracy for the realistic data, described in Sect. 5.2. The gradients were computed with four different GP kernels from their analytic expression of the first derivative. For computational reasons only the Lasso method was used to infer the networks. The underlying 2-way ANOVA model for the AUROC and AUPREC scores includes the GP **kernel** $K_k$ and the network structure $N_n$: $y_{knl} = K_k + N_n + \varepsilon _{knl}$, see Sect. 4.1 for details. Overlaps of the confidence intervals indicate that there is no significant difference in the achieved network reconstruction accuracy

**Fig. 14:**
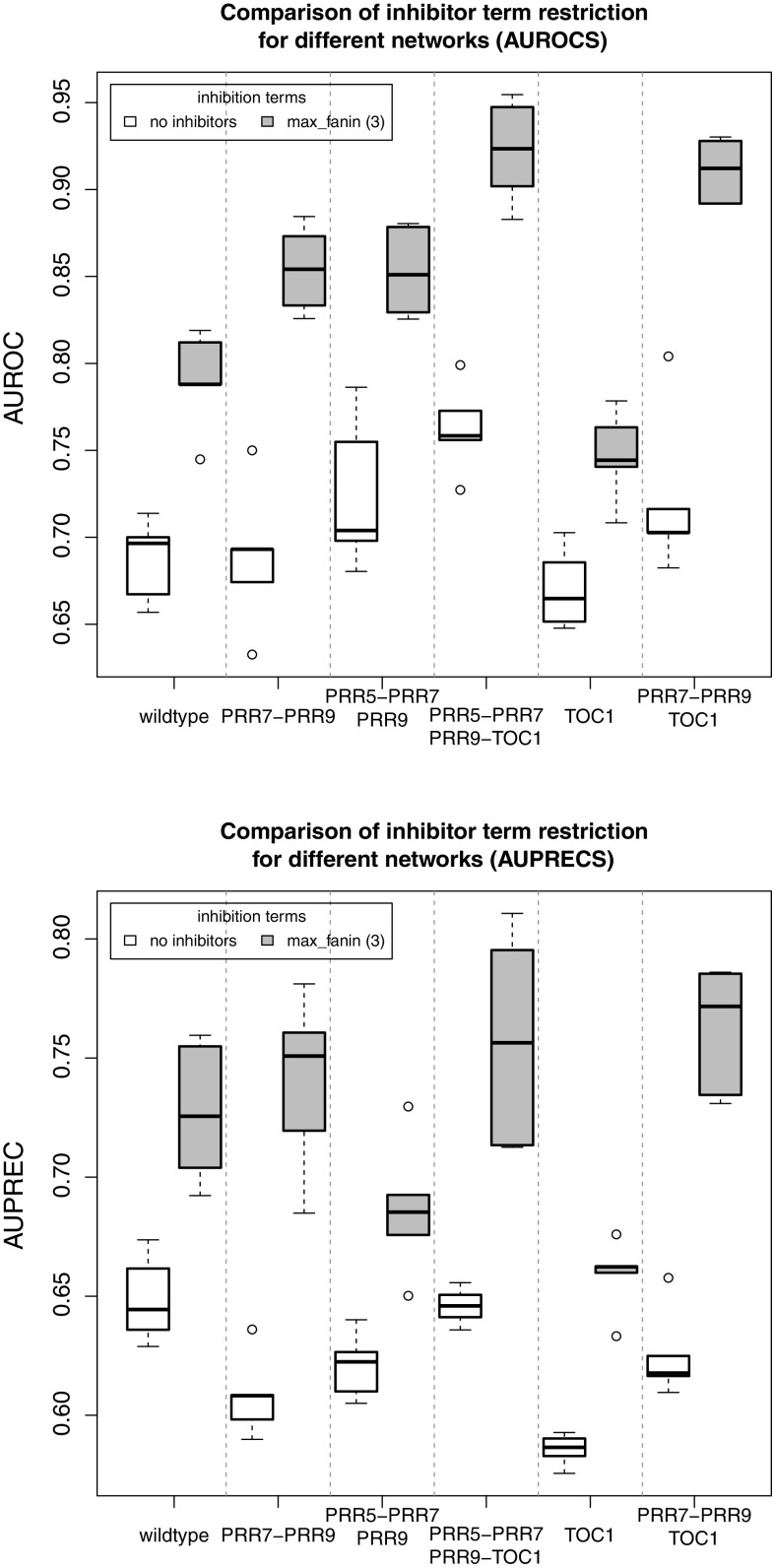
Network reconstruction accuracy. Network reconstruction accuracy in terms of AUROC (*top panel*) and AUPREC (*bottom panel*) scores for all six network structures included in our study. The figure compares the method proposed in our manuscript, *grey shading*, with a method without inhibitors (i.e. in which a fan-in restriction of 0 is imposed on the inhibitors), *white boxes*. Note that the latter method corresponds to the CheMA method as applied in Oates et al. (2014)

**Fig. 15:**
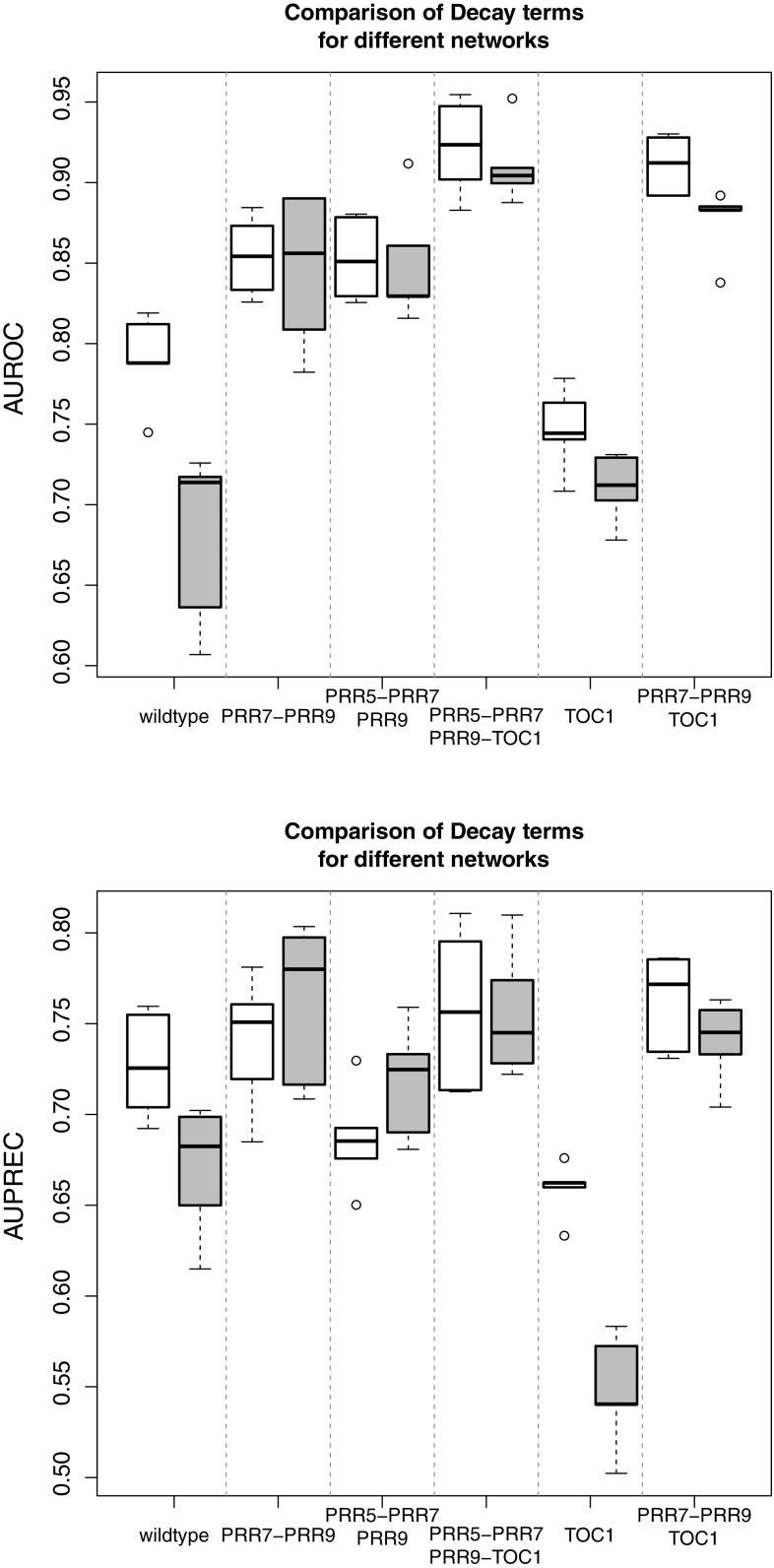
Evaluation of the influence of the mRNA decay term. The figure shows a comparison between the linear decay term of Eq. (50), applied in our study, white boxes, and the Michaelis–Menten decay term of Eq. (49), used in Oates et al. (2014), *grey boxes*. *Top panel* AUROC scores. *Bottom panel* AUPREC scores. The results for the six different network structures employed in our study are demarcated by vertical *dotted lines*

**Fig. 16:**
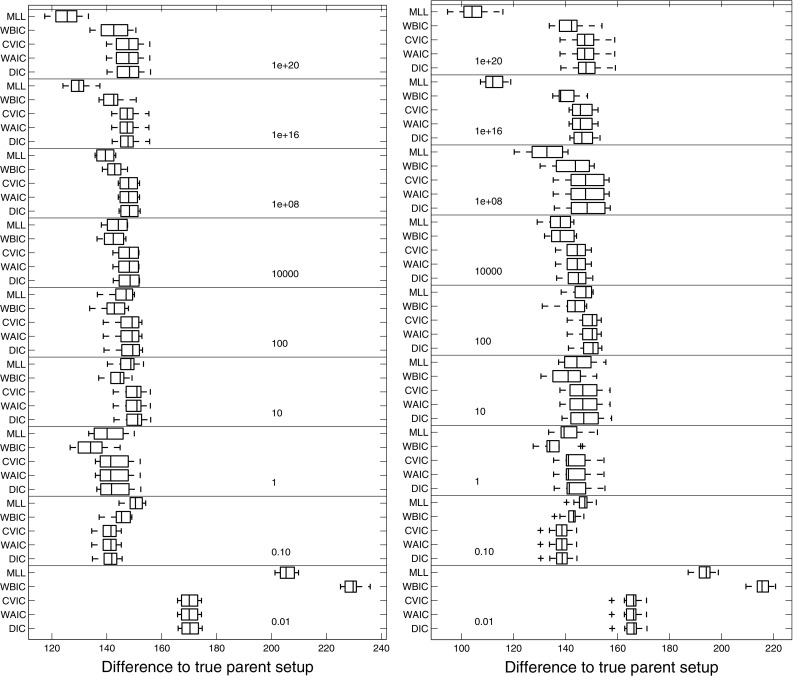
Score difference between the true model and an under-complex alternative model for four IC and MLL. For the synthetic data from Sect. 5.1 we compared the true model with an under-complex alternative model. As a statistical model we used the novel iCheMA model, and the MLL approach was implemented with the stabilized version of Chib’s method (**Chib**). The *panels* demarcated by the *horizontal lines* correspond to different spread factors $sf$ of the prior. For the difference between the *left* and the *right panel*, see the caption of Fig. 8

**Fig. 17:**
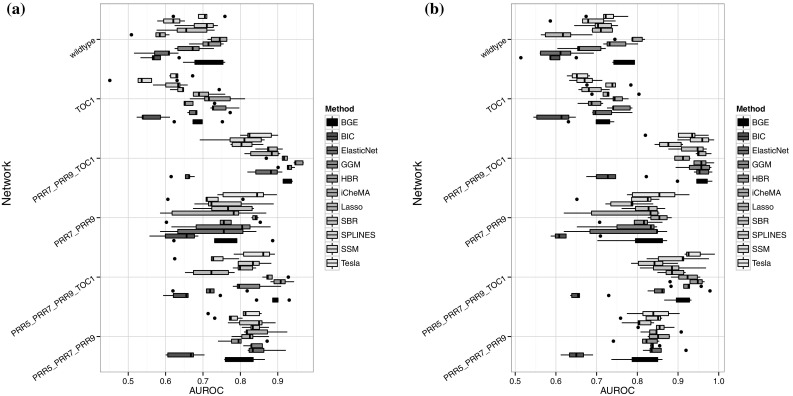
Detailed boxplots for different networks, methods and gradients. The boxplots depict the AUROC scores for various state-of-the-art network reconstruction methods (see Table 5, main paper) applied to the realistic data described in Sect. 5.2 (main paper) for two gradients; AUPREC scores are not shown. Each box contains the scores from five independent data instantiations. The boxplots illustrate the complexity of our results and motivate an evaluation scheme using the ANOVA model. **a** Difference Quotient Gradient. **b** Gaussian Process Gradient

**Fig. 18:**
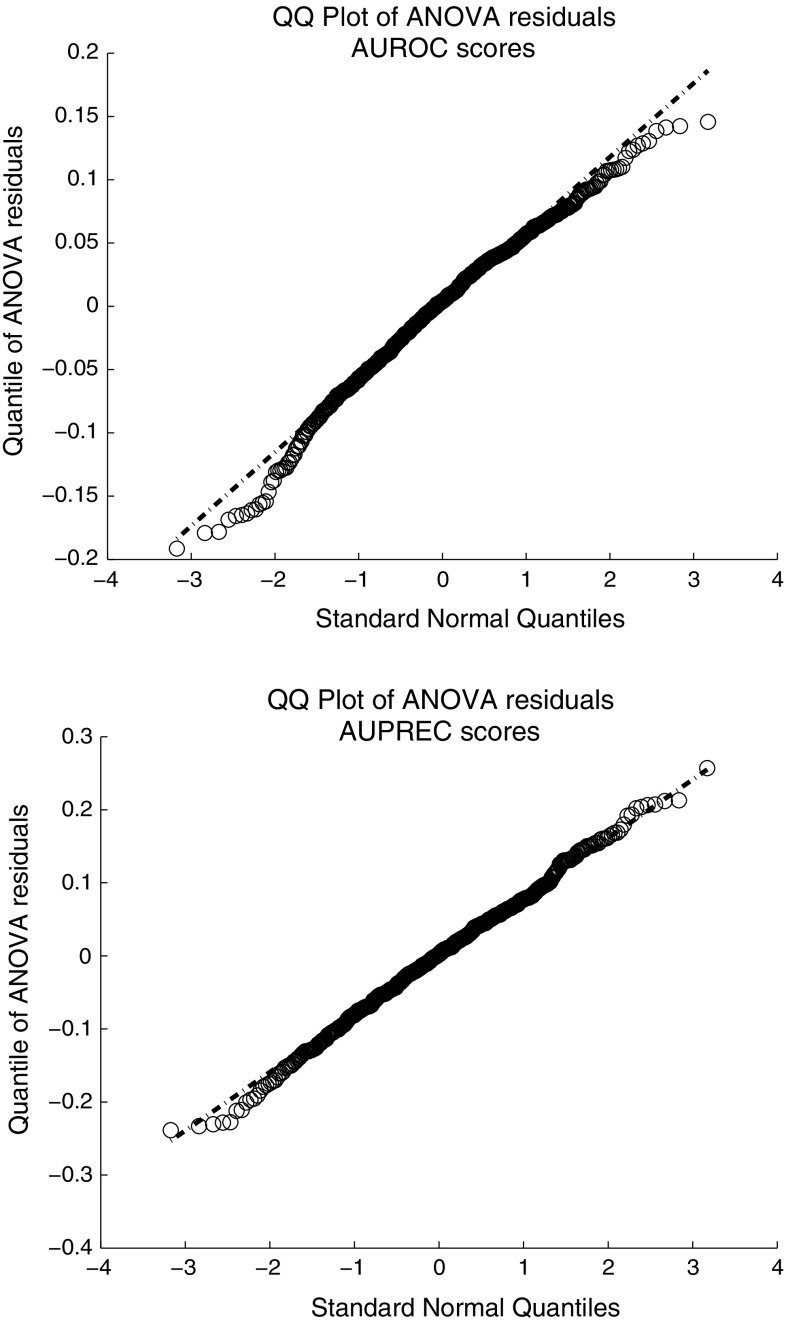
Residual diagnostic for the ANOVA model. The figure shows the Quantile–Quantile (QQ) plot of the residuals for the ANOVA model in Eq. (51) given AUROC and AUPREC scores. The actual quantiles (*vertical axis*) are plotted against the quantiles of the Gaussian distribution (*horizontal axis*). The linear relation indicates good agreement with the Gaussian distribution; the deviations for very low and high values in the left plot point to slightly longer tails of the residuals

**Fig. 19:**
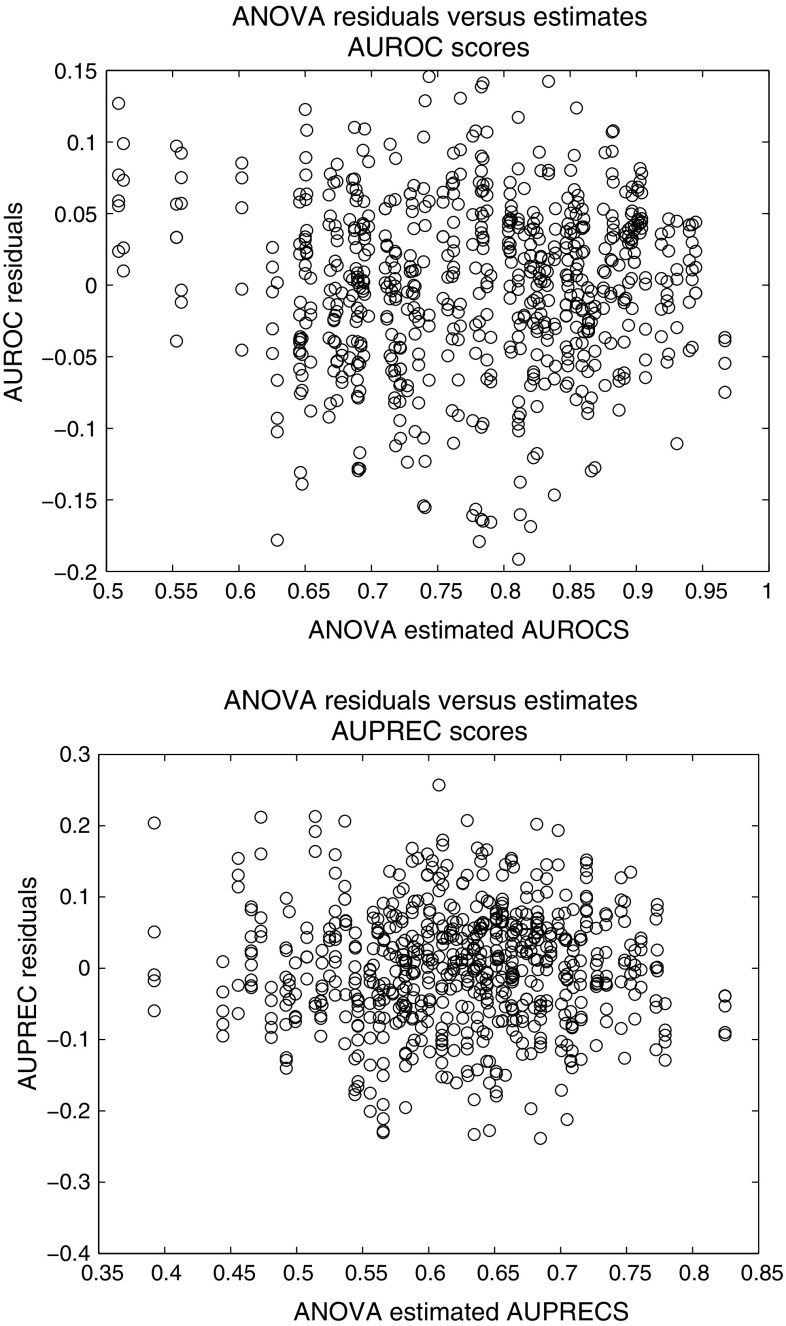
Residual scatter plot for the ANOVA model. Both panels show scatter plots of the residuals (*vertical axis*) against the AUROC values (*left plot*) and AUPREC values (*right plot*) fitted with the ANOVA model of Eq. (51) (*horizontal axis*)

**Fig. 20:**
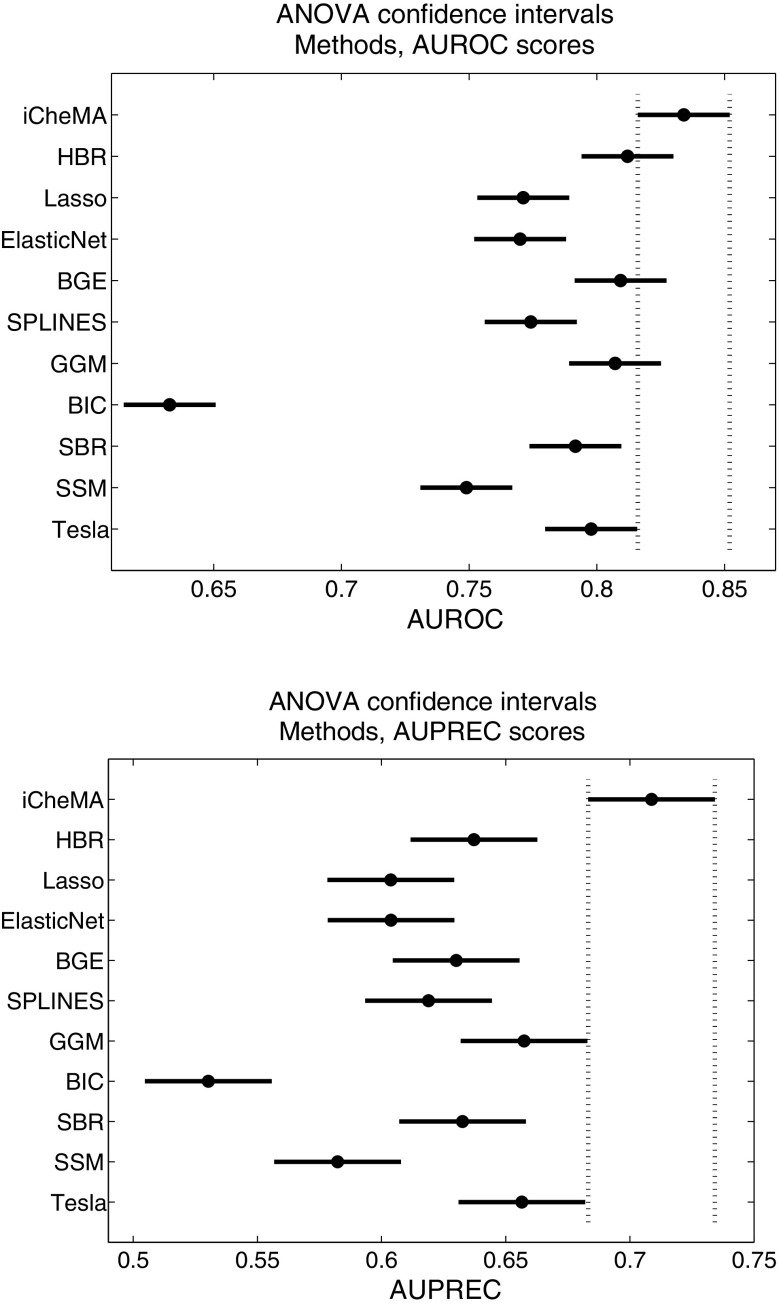
Confidence intervals for the effect of the network reconstruction method $M_m$, given the ANOVA model from Eq. (51)

**Fig. 21:**
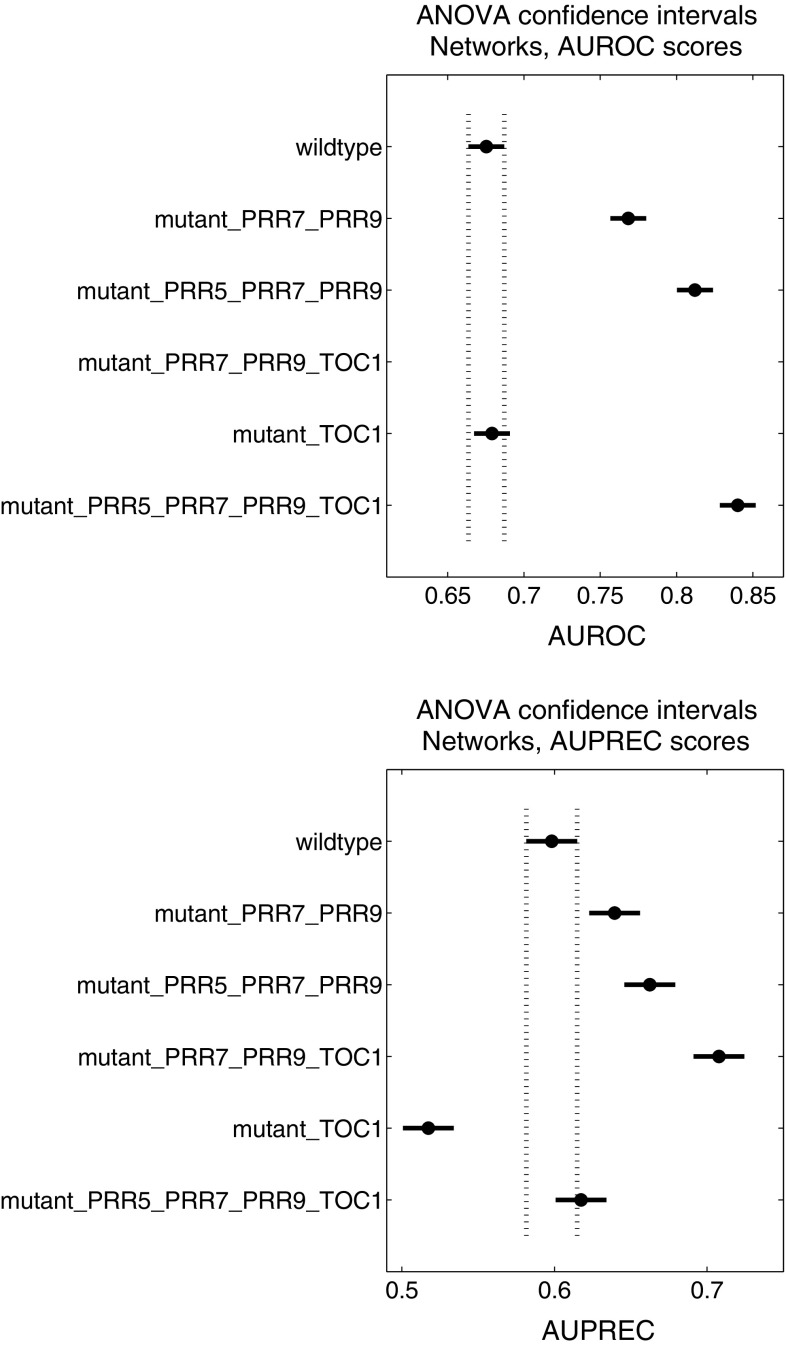
Confidence intervals for the effect of the network structure $N_n$, given the ANOVA model from Eq. (51)

**Fig. 22:**
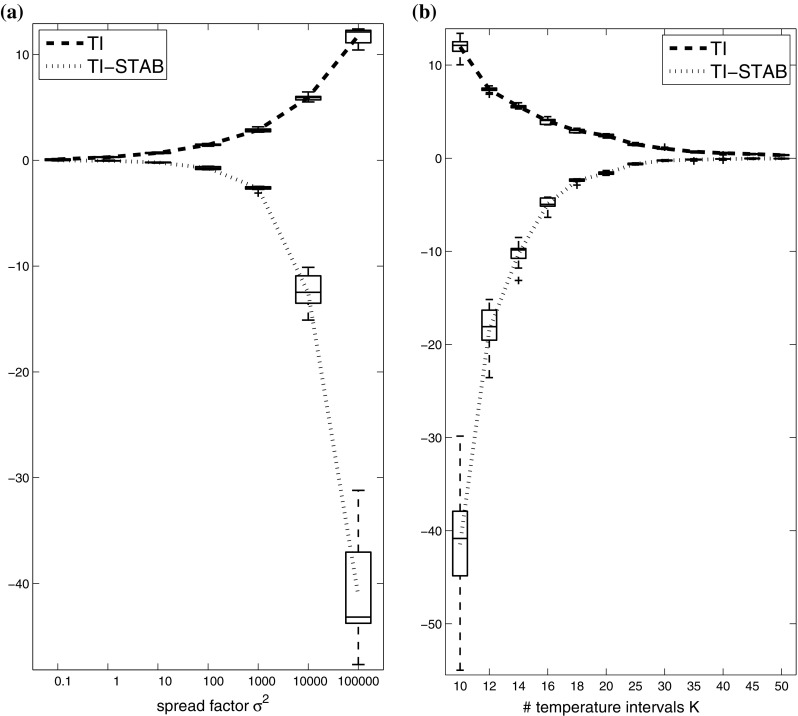
Deviation between the true and the approximated log Bayes factor differences for linear regression. The boxplots show distributions of the *log Bayes factor deviation scores*, defined in Eq. (60), for 10 linear regression data sets. *Dashed line*: **TI**. *Dotted line*: **TI-STAB**. Positive values indicate under- and negative values over-estimation of the true log Bayes factor differences. Panel **a** shows the deviations for $K=10$ and varying spread factors $\delta ^2$ in Eq. (52); Panel **b** shows the deviations for $\delta ^2=10^5$ in dependence on the number of discretization points *K* in Eq. (42)

**Fig. 23:**
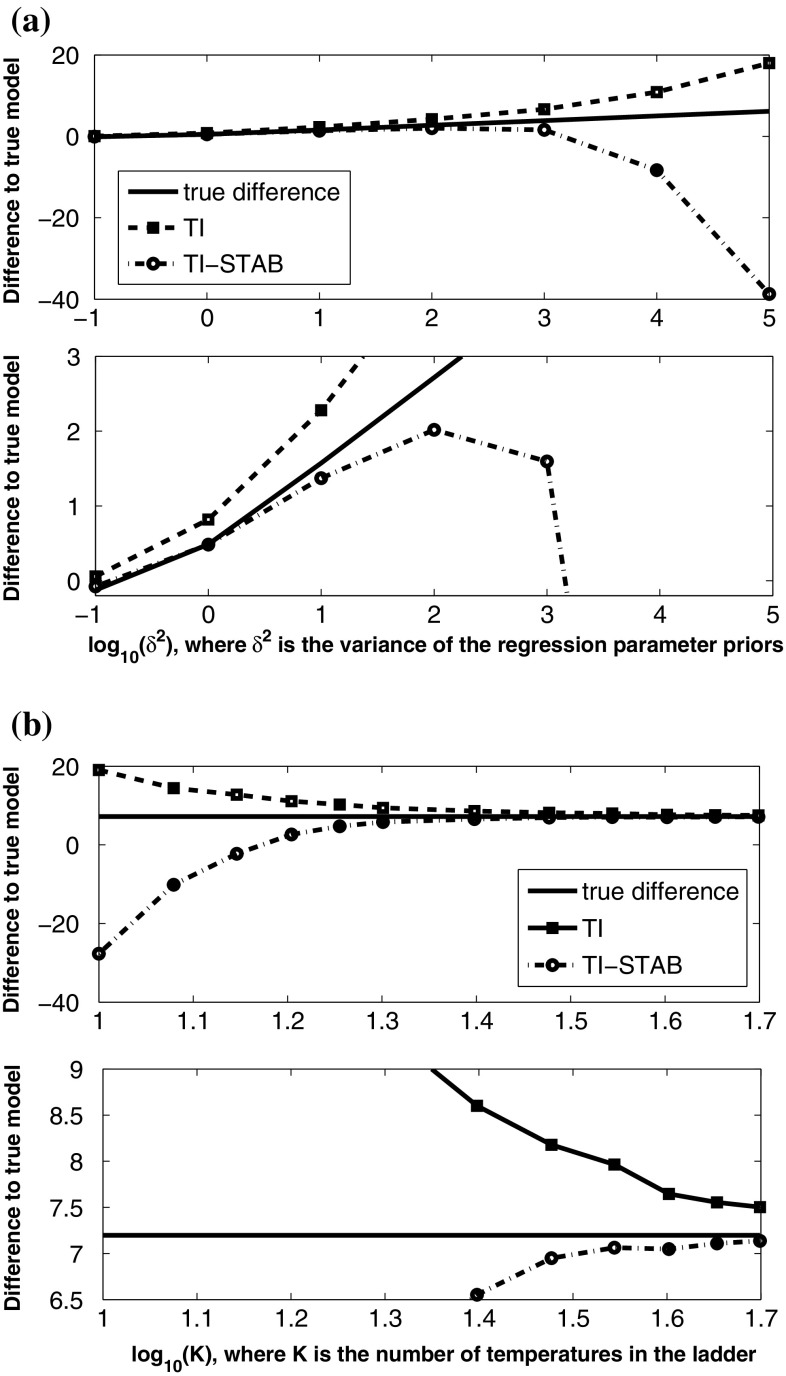
Log Bayes factor differences obtained with TI and TI-STAB for one representative linear regression data set. The plot shows the true and the approximated log Bayes factor differences for one representative Bayesian regression data set. **a** TI and TI-STAB were applied with $K=10$ discretization points and the hyperparameter $\delta ^2$ in Eq. (52) was varied. **b** $\delta ^2=10^5$ was kept fixed and the number of discretization points *K* in Eq. (42) was varied. For clarity, both *panels* show the same plot twice, using different scales on the *vertical axis*

**Fig. 24:**
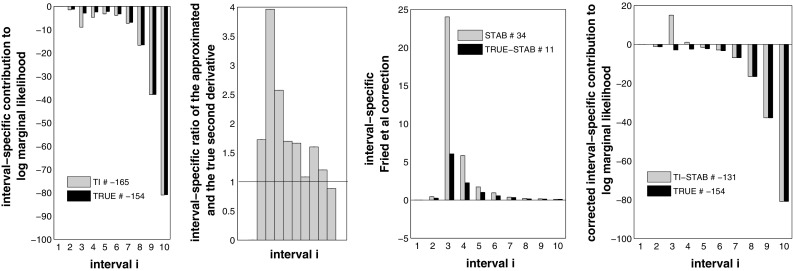
Diagnostic plots for the thermodynamic integration approaches: TI and TI-STAB. We have analysed the performance of **TI** and **TI-STAB** using one representative linear regression data set. The temperatures $\tau _0,\ldots ,\tau _K$ define intervals $[\tau _{i-1},\tau _{i}]$, and the index *i* refers to the *i*th interval ($i=1,\ldots ,K$). The integral in (37) can be approximated either by Eq. (38) (**TI**) or by Eq. (41) (**TI-STAB**). Consequently, for each interval *i*, the sub-integral from $\tau _{i-1}$ to $\tau _{i}$ is approximated by either one summand of Eq. (37) (**TI**), or by one summand of Eq. (38) (**TI-STAB**). The left panel compares the true values of the sub-integrals (i.e. the true interval-specific contributions to the MLL) with the values approximated by **TI** via the trapezium sum, i.e. the *K* summands from Eq. (38). The centre left panel shows, for each interval $[\tau _{i-1},\tau _{i}]$, the ratio of the left-hand and the right-hand side of Eq. (40), i.e. the ratio of the approximated and the true second derivative; see Appendix 1 for more details. In the *centre right panel* the *black bars* show the required interval-specific corrections, i.e. the mismatch between **TI** and **TRUE** (observed in the *left panel*), and the *grey bars* represent the correction terms that result from the application of **TI-STAB**. The *right panel* shows the interval-specific contributions of **TI-STAB**. The *black bars* represent the true values of the sub-integrals, while the *grey bars* refer to **TI-STAB**. The sums of the **TI** contributions (*grey bars* in the *left panel*) and the correction terms (*grey bars* in the centre *right panel*) give the final value of the thermodynamic integral obtained with **TI-STAB**

**Fig. 25:**
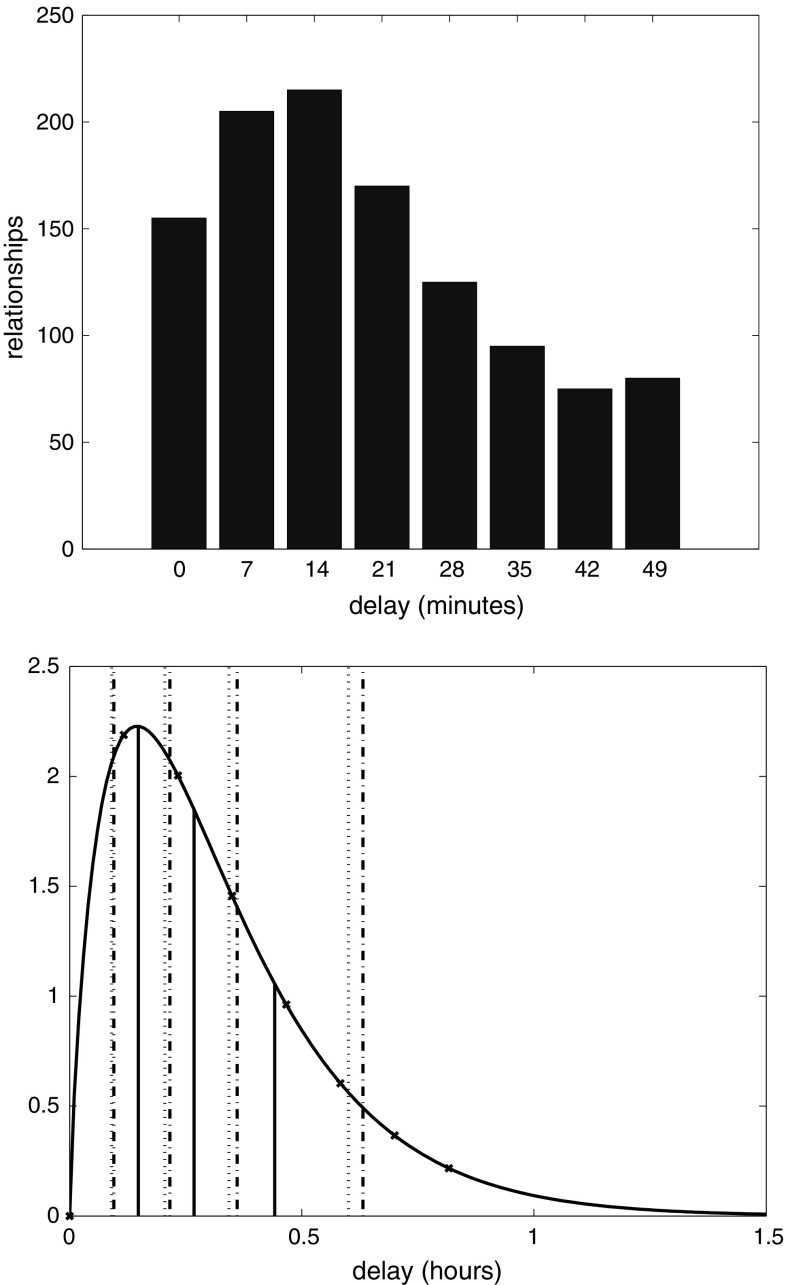
Discrete gamma distribution for time-delays. The bar plot shows the prior distribution of regulatory time-delays extracted from Ota et al. (2003). The plot in the bottom is the corresponding gamma density, divided in $k=4$ categories with equal probability mass. The dotted lines are the medians in each category, corresponding to Eq. (68), and the dash-dotted lines are the rescaled medians, according to Eq. (69); they represent the support points of the discrete gamma distribution, following the procedure of Yang (1994)

**Fig. 26:**
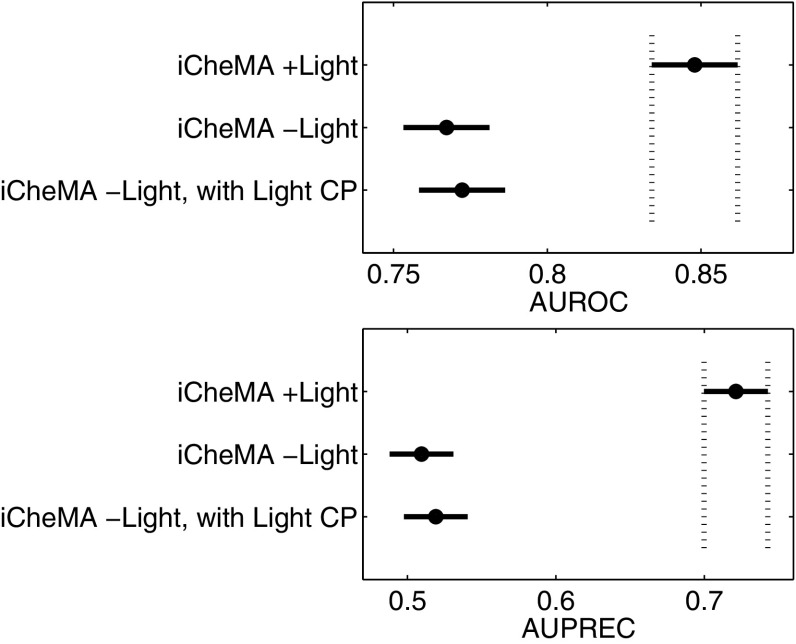
Sensitivity to Light*P for the realistic data from Biopepa. The *top row* in each boxplot displays the AUROC and AUPREC scores for iCheMA, applied to the realistic data from Sect. 5.2 that include the hypothetical light signal ‘Light*P’ as covariable. The *middle rows * correspond to a lack of this covariable. The *bottom rows* also lack ‘Light*P’, but as an alternative, simulate the light influence with a change-point process that segments the data into a light and a dark phase. For each parent configuration, the MLL scores are computed for both phases separately and then added to give the final MLL score

**Fig. 27:**
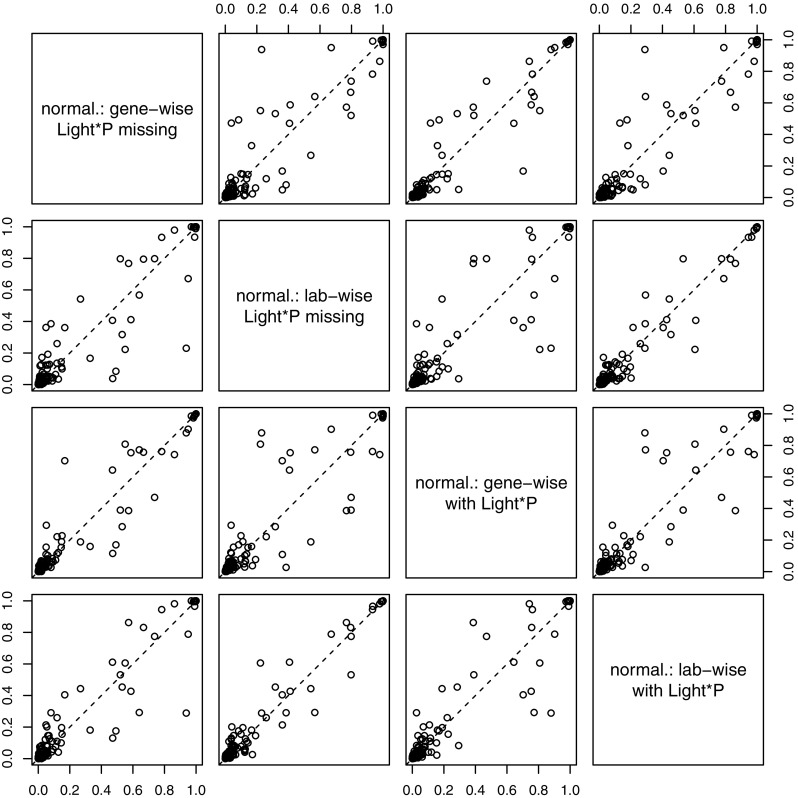
Influence of data normalization and an artificial light signal on real data. The scatter plots illustrate the changes of the marginal edge posterior probabilities inferred with iCheMA under different conditions of the DIURNAL MocklerLab data. Z-score normalization was carried out for each gene over all measurements including measurement from different labs (‘gene-wise’), and for each gene and each set of experiments that originate from the same experimental lab (‘lab-wise’). The covariable ‘Light*P’ resembles a binary light indicator multiplied by a hypothetical light sensing protein ‘P’. It is either present in the set of potential regulators (‘with Light*P’) or excluded (‘Light*P missing’)

**Fig. 28:**
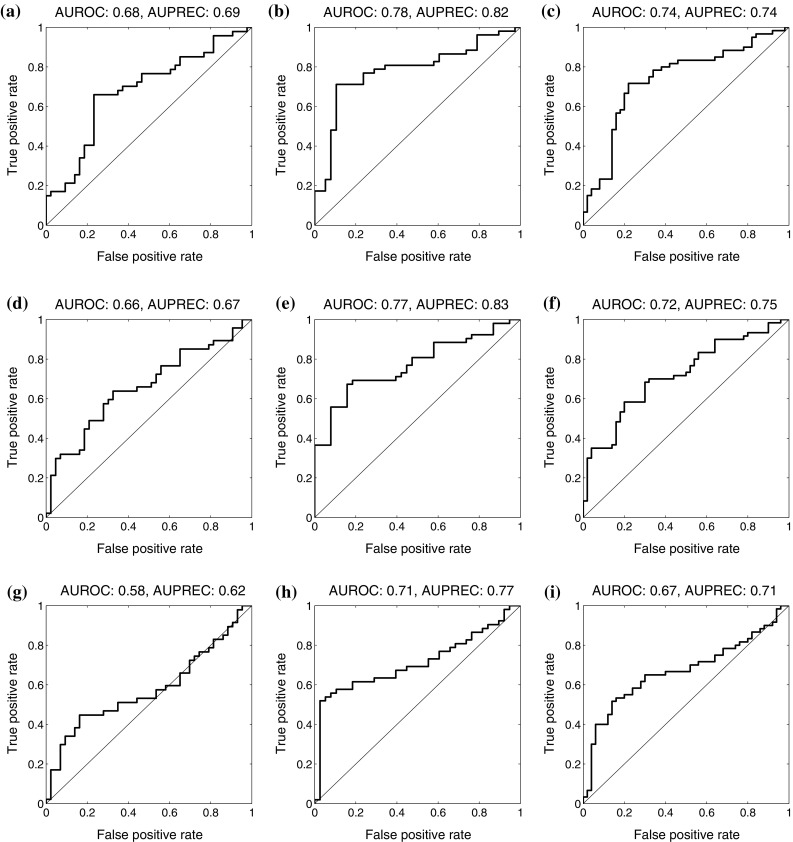
ROC curves comparing iCheMA with the F2014 (b, e, h) and P2013 (a, d, g) networks for the gene expression data from *A. thaliana*. **a**–**c** show ROC curves based on iCheMA with a gradient derived from a periodic (PER) kernel and allowing for transcriptional time delays. **d**–**f** show ROC curves with the same setup but without transcriptional time delay (td = 0 h), and **g**–**i** show ROC curves based on a gradient derived from a RBF kernel and without transcriptional time delay. The numbers at the top of each *panel* show the areas under the ROC curves (AUROC) and precision-recall curves (AUPREC). **c**, **f**, and **i** display the comparison with the F2014 network and the additional RVE8 gene included, which is not present in the P2013 network

**Fig. 29:**
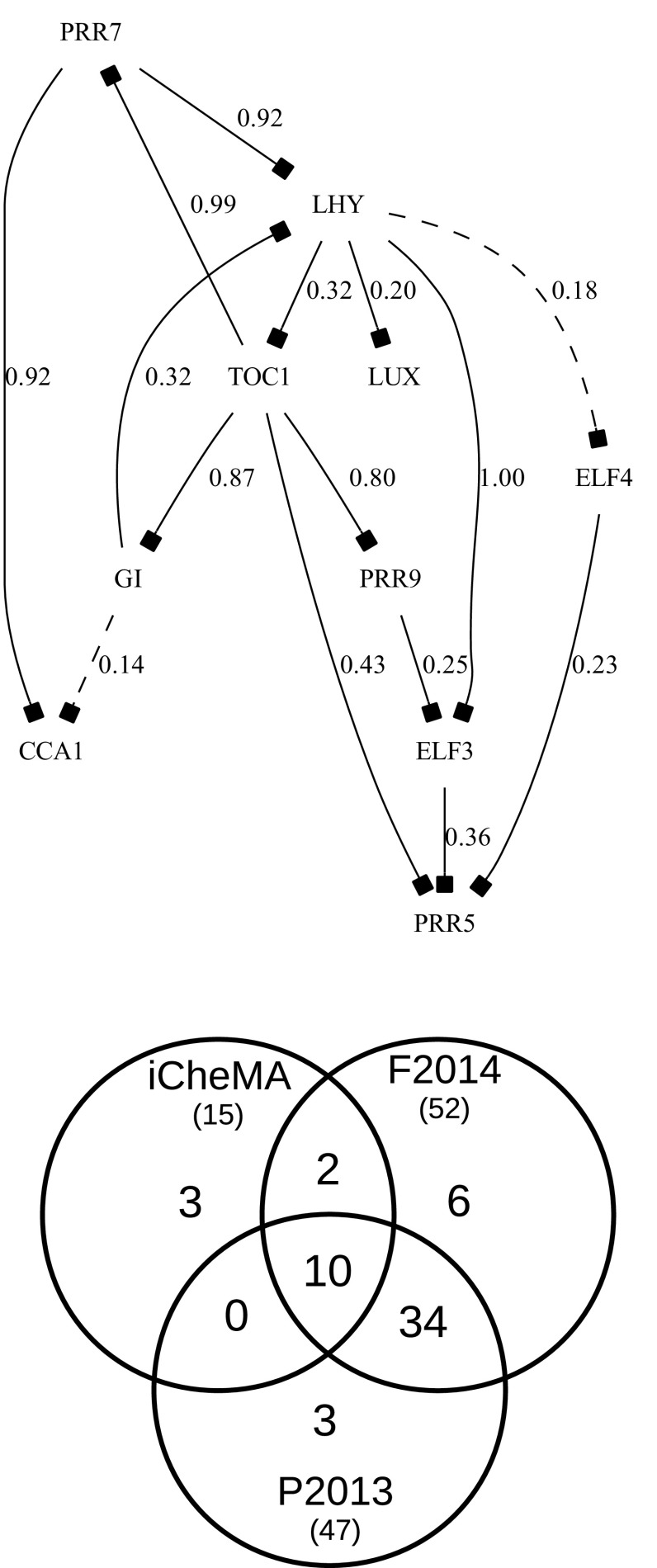
Predicted clock gene interactions for *A. thaliana*. The *top panel* shows the clock gene network in *A. thaliana*, as predicted with iCheMA from the gene expression data described in Sect. 5.3. The numbers on the edges denote predicted marginal posterior probabilities as defined in Sect. 3.3. Following Grzegorczyk et al. (2015), the edge inclusion threshold was set to 0.11. The gradients were derived from a GP with periodic kernel and time-delayed effects were considered by marginalizing over four different time-delays. We only considered repressive interaction types, symbolized by a *small box* at the end of a directed edge, as this is the most likely scenario for gene interactions inside the clock. The *lower panel* displays a Venn diagram with the number of matching interactions among the predicted network, the P2013 (Pokhilko et al. 2013), and F2014 network (Fogelmark and Troein 2014). The numbers in brackets correspond to the total amount of edges in each network

## Appendices

### Appendix 1: Methodology

#### Model selection

The ultimate objective of inference is model selection, i.e. to infer the *n* regulator sets $\pi _i$ ($i=1,\ldots ,n$) of the interaction processes described by Eq. (6). Thereby each regulator set $\pi _i$ also includes a binary variable that indicates, for each regulator, the type of regulation: activation versus inhibition. For notational clarity, let $\varvec{\theta }_i=({\mathbf{V }}_i,{\mathbf{K}}_i,\sigma _{i}^{2},\delta _{i}^{2})$ denote the model-specific parameters, and recall that ${\mathbf{y}}_i=(y_i(t_1),\ldots ,y_i(t_T))^{\top }$ denotes the observed (or approximated) gradients for species $x_i$.

DIC is an improvement on the classical information criteria AIC and BIC and is defined as

28 $$\begin{aligned}&\mathrm {DIC}(\pi _i) \!=\! 2 \log p({\mathbf{y}}_i|\overline{\varvec{\theta }_i},\pi _i) \nonumber \\&\quad -\,4 \int \log p({\mathbf{y}}_i|\varvec{\theta }_i,\pi _i) p(\varvec{\theta }_i|\pi _i,{\mathbf{y}}_i) d\varvec{\theta }_i \end{aligned}$$

where $\overline{\varvec{\theta }_i} = \int \varvec{\theta }_i p(\varvec{\theta }_i|\pi _i,{\mathbf{y}}_i) d\varvec{\theta }_i$ is the posterior mean of the parameters. In practice, the integrals are approximated by sums over parameters approximately sampled from the posterior distribution $p(\varvec{\theta }_i|\pi _i,{\mathbf{y}}_i)$ with MCMC. The marginal likelihood is defined as

29 $$\begin{aligned} p({\mathbf{y}}_i|\pi _i) \; = \; \int p({\mathbf{y}}_i|\varvec{\theta }_i,\pi _i) p(\varvec{\theta }_i|\pi _i) d\varvec{\theta }_i \end{aligned}$$

The essential difference between Eqs. (28) and (29) is that DIC is defined as an expectation with respect to the posterior distribution, $p(\varvec{\theta }_i|\pi _i,{\mathbf{y}}_i)$, whereas the marginal likelihood is defined as an expectation with respect to the prior distribution, $p(\varvec{\theta }_i|\pi _i)$. This has two consequences. First, the marginal likelihood is affected by the choice of prior, and in particular is known to increasingly penalize a more complex model as the prior $p(\varvec{\theta }_i|\pi _i)$ becomes more diffuse (Lindley’s paradox, see Lindley 1957). DIC, on the other hand, is unaffected by diffuse priors: the more diffuse a prior, the less of an influence it has on the posterior. The second consequence is that computing the log marginal likelihood (MLL) is more onerous, as we will discuss below.

DIC is routinely used for model selection in Winbugs (Lunn et al. 2012), and the paper in which it was introduced (Spiegelhalter et al. 2002) has got over 5000 citations at the time of the submission of the present article. However, a limitation of DIC is that it can only be applied to non-singular models. A statistical model is said to be non-singular if the map between parameters and probability distributions is one-to-one and if its Fisher information matrix is positive definite. This excludes several classes of important models, like mixture models. In singular statistical models, the maximum likelihood estimator does not satisfy asymptotic normality, and DIC loses its asymptotic justification. To intuitively see from where the difficulty arises, consider a likelihood with two equivalent modes, resulting e.g. from different label choices of a mixture model. The posterior expectation $\overline{\varvec{\theta }_i}$ will then *not* be representative of the data, as it will lie in a region of low likelihood between the two modes.

A generalization of DIC that can be applied to singular models is the WAIC proposed in Watanabe (2010). Recall that the data vector ${\mathbf{y}}_i$ contains *T* individual observations $\{y_i(t_1),\ldots ,y_i(t_T)\}$. Then WAIC is defined as

$$\begin{aligned}&\mathrm {WAIC} (\pi _i) = \\&\quad -2 \sum _{j=1}^T \bigg ( \log \big [ \mathbb {E}\{p( y_i(t_j)|\varvec{\theta }_i)\} \big ] - \mathbb {V}\big \{ \log [P(y_i(t_j)|\varvec{\theta }_i)] \big \} \bigg ) \end{aligned}$$

where $\mathbb {E}(.)$ and $\mathbb {V}(.)$ stand for the posterior mean and variance, i.e. the mean and variance with respect to the posterior distribution $p(\varvec{\theta }_i|{\mathbf{y}}_i,\pi _i)$. The essential difference between DIC and WAIC is that for the latter, the posterior expectation and variance are defined in data space, $y_i$, and not in parameter space, $\varvec{\theta }_i$. This leads to greater numerical stability and is the reason why WAIC is not restricted to non-singular models.

Both DIC and WAIC are approximations to the Bayesian leave-one-out cross-validation (BLOOCV) estimate:

30 $$\begin{aligned} \mathrm {BLOOCV}(\pi _i)= & {} \sum _{j=1}^T \log p\left( y_i(t_j)|{\mathbf{y}}_i^{[t_j]}\right) \nonumber \\= & {} \sum _{j=1}^T \log \int p(y_i(t_j)|\varvec{\theta }_i) p\left( \varvec{\theta }_i|{\mathbf{y}}_i^{[t_j]}\right) d\varvec{\theta }_i \nonumber \\ \end{aligned}$$

where ${\mathbf{y}}_i^{[t_j]} = \{y_i(t_1),\ldots ,y_i(t_{j-1}),y_i(t_{j+1}),\ldots ,y_i(t_T)\}$ is the leave-one-out data with sample $y_i(t_j)$ removed. In fact, WAIC can be shown to be asymptotically equivalent to BLOOCV (Watanabe 2010), and the relation between DIC and BLOOCV is discussed in Gelman et al. (2014b). In practice, the integral on the right-hand side of Eq. (30) is replaced by a sum over a sample from an MCMC simulation:

31 $$\begin{aligned} \mathrm {BLOOCV}(\pi _i) \; = \; \sum _{j=1}^T \log \left( \frac{1}{K}\sum _{k=1}^K p(y_i(t_j)|\varvec{\theta }_{i,j,k}) \right) \end{aligned}$$

where $\{\varvec{\theta }_{i,j,k}\}_{k=1,\ldots ,K}$ is a sample from $p(\varvec{\theta }_i|{\mathbf{y}}_i^{[t_j]})$. The practical difficulty is the high computational cost, as a consequence of having to rerun the MCMC simulations *T* times, on *T* separate data sets ${\mathbf{y}}_i^{[t_1]},\ldots ,{\mathbf{y}}_i^{[t_T]}$. An approximation to BLOOCV that only requires a single MCMC run (on ${\mathbf{y}}_i$) was proposed in Gelfand et al. (1992)

32 $$\begin{aligned} \tilde{p}(y_i(t_j)|{\mathbf{y}}_i^{[t_j]})= & {} \frac{1}{\mathbb {E}_{\mathrm {post}}[1/p(y_i(t_j)|\varvec{\theta }_i)]} \nonumber \\= & {} \frac{1}{\int [1/p(y_i(t_j)|\varvec{\theta }_i)]p(\varvec{\theta }_i|{\mathbf{y}}_i)d\varvec{\theta }_i} \end{aligned}$$

which in practice is computed by

33 $$\begin{aligned} \tilde{p}\left( y_i(t_j)|{\mathbf{y}}_i^{[t_j]}\right) \; = \; \frac{R}{\sum _{i=1}^R[1/p(y_1(t_j)|\varvec{\theta }_{i,r})]} \end{aligned}$$

where $\{\varvec{\theta }_{i,r}\}_{r=1,\ldots ,R}$ is an MCMC sample from $p(\varvec{\theta }_i|{\mathbf{y}}_i)$. From these distributions in Eq. (33) one obtains the so-called CVIC as follows:

$$\begin{aligned} \mathrm {CVIC}(\pi _i) \; = \; -2 \sum _{j=1}^T \log \tilde{p}\left( y_i(t_j)|{\mathbf{y}}_i^{[t_j]}\right) \end{aligned}$$

For computing the MLL, it is known that approximating the integral in Eq. (29) with Monte Carlo based on a direct sample from the prior $p(\varvec{\theta }_i|\pi _i)$ shows, in general, extremely poor convergence. In the present article, we compare two improved and established methods: Chib’s method and thermodynamic integration. Chib’s method is based on

34 $$\begin{aligned} p({\mathbf{y}}_i|\pi _i) \; = \; \frac{p({\mathbf{y}}_i|\tilde{\varvec{\theta }}_i,\pi _i)p(\tilde{\varvec{\theta }}_i|\pi _i)}{p(\tilde{\varvec{\theta }}_i|\pi _i,{\mathbf{y}}_i)} \end{aligned}$$

where the posterior near some selected ‘pivot’ parameters $\tilde{\varvec{\theta }}_i$, $p(\tilde{\varvec{\theta }}_i|\pi _i,{\mathbf{y}}_i)$, is approximated with MCMC; see Chib and Jeliazkov (2001) for details. TI is based on the power posteriors

35 $$\begin{aligned} p(\varvec{\theta }_i|G,{\mathbf{y}}_i,\tau ) \; = \; \frac{p({\mathbf{y}}_i|\varvec{\theta }_i,\pi _i)^{\tau }p(\varvec{\theta }_i|\pi _i)}{Z({\mathbf{y}}_i,\pi _i,\tau )} \end{aligned}$$

with

36 $$\begin{aligned} Z({\mathbf{y}}_i,\pi _i,\tau ) \; = \; \int p({\mathbf{y}}_i|\varvec{\theta }_i',\pi _i)^{\tau }p(\varvec{\theta }_i'|\pi _i) d\varvec{\theta }_i' \end{aligned}$$

from which the MLL is computed via

37 $$\begin{aligned} \log (p({\mathbf{y}}_i|\pi _i)) \; = \; \int _{0}^{1} \mathbb {E}_{\varvec{\theta }_i,\tau } \big [ \log p({\mathbf{y}}_i|\varvec{\theta }_i,\pi _i) \big ] d \tau \end{aligned}$$

Here $\mathbb {E}_{\varvec{\theta }_i,\tau }$ is an expectation with respect to the power posterior in Eq. (35). In practice, these expectations are computed for various ’temperatures’ $\tau$ simultaneously with population MCMC, as described in Friel and Pettitt (2008). The integral in Eq. (37) is one-dimensional and can be numerically approximated e.g. with a trapezium sum:

38 $$\begin{aligned}&\log p({\mathbf{y}}_i|\pi _i)\nonumber \\&\quad \approx \sum _{k=1}^K \frac{\tau _k-\tau _{k-1}}{2} \left\{ \mathbb {E}_{\varvec{\theta }_i,\tau _k}\big [\log p({\mathbf{y}}_i|\varvec{\theta }_i,\pi _i)\big ] \right. \nonumber \\&\quad \left. +\, \mathbb {E}_{\varvec{\theta }_i,\tau _{k-1}}\big [\log p({\mathbf{y}}_i|\varvec{\theta }_i,\pi _i) \big ] \right\} \end{aligned}$$

A potentially numerically more stable alternative, which we refer to as TI-STAB, was proposed in Friel et al. (2013). Friel et al. show that:

39 $$\begin{aligned} \frac{d}{dt} \left\{ \mathbb {E}_{\varvec{\theta }_i,t}[\log (p({\mathbf{y}}_i|\varvec{\theta }_i,\pi _i))] \right\} _{t=\tau } = \mathbb {V}_{\varvec{\theta }_i,\tau }(\log (p({\mathbf{y}}_i|\varvec{\theta }_i,\pi _i))\nonumber \\ \end{aligned}$$

where $\mathbb {V}_{\varvec{\theta }_i,\tau _k}$ is the variance w.r.t. the power posterior in Eq. (35). The second derivative of $\mathbb {E}_{\varvec{\theta }_i,t}[\log (p({\mathbf{y}}_i|\varvec{\theta }_i,\pi _i))]$ at a point $\tau \in [\tau _{k-1},\tau _{k}]$ can then be approximated by the differential quotient of $\mathbb {E}_{\varvec{\theta }_i,t}[\log (p({\mathbf{y}}_i|\varvec{\theta }_i,\pi _i))]$’s first derivative from Eq. (39):

40 $$\begin{aligned}&\frac{d^2}{dt^2} \left\{ \mathbb {E}_{\varvec{\theta }_i,t}[\log (p({\mathbf{y}}_i|\varvec{\theta }_i,\pi _i))] \right\} _{t=\tau } \nonumber \\&\quad \approx \frac{\mathbb {V}_{\varvec{\theta }_i,\tau _{k}}(\log (p({\mathbf{y}}_i|\varvec{\theta }_i,\pi _i))-\mathbb {V}_{\varvec{\theta }_i,\tau _{k-1}}(\log (p({\mathbf{y}}_i|\varvec{\theta }_i,\pi _i))}{\tau _{k}-\tau _{k-1}}\nonumber \\ \end{aligned}$$

Friel et al. (2013) then employ the corrected trapezoidal rule^8^ to compute each sub-integral $\int _{\tau _{k-1}}^{\tau _{k}}\mathbb {E}_{\varvec{\theta }_i,\tau }[\log (p({\mathbf{y}}_i|\varvec{\theta }_i,\pi _i))] d\tau$. This yields:

41 $$\begin{aligned}&\log (p({\mathbf{y}}_i|\pi _i)) = \int _{0}^{1} \mathbb {E}_{\varvec{\theta }_i,\tau } \big [ \log p({\mathbf{y}}_i|\varvec{\theta }_i,\pi _i) \big ] d \tau \nonumber \\&\quad = \sum _{k=1}^K \int _{\tau _{k-1}}^{\tau _k} \mathbb {E}_{\varvec{\theta }_i,\tau } \big [ \log p({\mathbf{y}}_i|\varvec{\theta }_i,\pi _i) \big ] d \tau \nonumber \\&\quad \approx \sum _{k=1}^K \frac{\tau _k-\tau _{k-1}}{2} \left\{ \mathbb {E}_{\varvec{\theta }_i,\tau _k}\big [\log p({\mathbf{y}}_i|\varvec{\theta }_i,\pi _i)\big ] \right. \nonumber \\&\qquad \left. +\, \mathbb {E}_{\varvec{\theta }_i,\tau _{k-1}}\big [\log p({\mathbf{y}}_i|\varvec{\theta }_i,\pi _i) \big ] \right\} \nonumber \\&\qquad -\,\sum _{k=1}^K \frac{(\tau _k-\tau _{k-1})^2}{12} \left\{ \mathbb {V}_{\varvec{\theta }_i,\tau _k}\big [\log p({\mathbf{y}}_i|\varvec{\theta }_i,\pi _i)\big ] \right. \nonumber \\&\left. \qquad -\,\mathbb {V}_{\varvec{\theta }_i,\tau _{k-1}}\big [\log p({\mathbf{y}}_i|\varvec{\theta }_i,\pi _i) \big ] \right\} \end{aligned}$$

Since the contributions to the integral come essentially from the ’temperature’ range $\tau \rightarrow 0$, the choice of discretization points is typically chosen of the form:

42 $$\begin{aligned} \tau _k = \left( \frac{k}{K-1}\right) ^m \end{aligned}$$

for $\quad 0\le k \le K-1$, where *K* is the number of discretization points. For our study, we selected $K=10$ and $m \in \{4,8\}$.

Thermodynamic integration is more expensive than the other methods reviewed in this section, due to the need to run MCMC simulations for a range of smoothing levels $\tau _k$. In principle a simplification could be achieved based on the mean value theorem of differential calculus

43 $$\begin{aligned} log(p({\mathbf{y}}_i|\pi _i))= & {} \frac{\log Z({\mathbf{y}}_i,\pi _i,1)-\log Z({\mathbf{y}}_i,\pi _i,0)}{1-0} \nonumber \\= & {} \left[ \frac{d}{d\tau }\log Z({\mathbf{y}}_i,\pi _i,\tau ) \right] _{\tau = \tau ^*} \nonumber \\= & {} \mathbb {E}_{\varvec{\theta }_i,\tau ^*} \big \{\log p({\mathbf{y}}_i|\pi _i,\varvec{\theta }_i)\big \} \end{aligned}$$

where $\tau ^* \in [0,1]$, and $Z({\mathbf{y}}_i,\pi _i,\tau )$ was defined in Eq. (36). The value $\tau ^*$ is unknown. An asymptotically optimal approximation, derived in Watanabe (2013), is

44 $$\begin{aligned} \tau ^* \; = \; \frac{1}{\log (T)} \end{aligned}$$

The corresponding score is known as the WBIC:

45 $$\begin{aligned} {\textit{WBIC}}(\pi _i) \;= \; \mathbb {E}_{\varvec{\theta }_i,\tau ^*} \big \{\log p({\mathbf{y}}_i|\pi _i,\varvec{\theta }_i)\big \} \end{aligned}$$

In summary, we compare five model selection methods: **DIC**, **WAIC**, **CVIC**, **WBIC**, and the MLL. For MLL, we compare four alternative numerical methods: Chib’s method as proposed in Chib and Jeliazkov (2001) (**Chib naive**), a stabilized version of Chib’s method (**Chib**), proposed here, thermodynamic integration with the trapezoid rule (**TI**), see Eq. (38), and thermodynamic integration with a numerical correction (**TI-STAB**), see Eq. (41).

#### Causal sufficiency

Causal inference is a fundamental research topic, which is beyond the scope of the present study. What we pursue is model selection: given a set of candidate mathematical models, find the one that is most consistent with the data. This concerns the particular challenge of selecting explanatory variables within the proposed mathematical modelling context, which in terms of transcriptional regulation are the putative transcriptional regulators.

Ultimately, statistical inference faces two separate model selection problems. The first one is: given a mathematical description of the regulatory processes, find the network structure that is most consistent with the data. This problem is well-defined and the focus of our investigations. However, there is a second model selection problem underneath: what is the correct mathematical description of the regulatory processes? This problem is open-ended, in that there is an infinite number of regulatory processes that one could potentially consider. What we have focussed on is transcriptional regulation. It could be argued that this view is limited, and that additional processes related to micro-RNA regulation and unobserved species should ideally be taken into consideration. Besides the higher degree of computational complexity, beyond what is practically feasible with our computational resources, the set of interaction processes will potentially always have to be further extended, as our current knowledge of molecular biology is limited, and new scientific insights gained in the future, related to new molecular processes hitherto unknown, will have to be accommodated.

We have studied and quantified the consequences of model misspecification in two ways. First, we have compared our additive model of transcriptional regulation with models that are biologically less realistic. This is discussed in Sect. 6.4. Secondly, we have evaluated the effect of a misspecification related to ignoring protein interactions. This influence is quantified in Sect. 6.5. The consequences of ignoring processes related e.g. to micro-RNA regulation could in principle also be tested in a simulation study, but that is beyond the scope of the present article paper.

In more general terms, the question is how much we can trust the model selected from a model family that is incomplete and, most likely, oversimplified. But that is a fundamental problem affecting science in general. Our view of the world is never complete, and we need to base model selection on what is known or computationally feasible at the time the work is carried out. It is worth to recall George Box famous citation in this context, that all models are wrong, but some are useful. The considerable agreement of the network structures inferred from the real gene expression data with those independently published in the mathematical biology literature, as discussed in Appendix 4, confirms the usefulness of the presented inference framework despite its inevitable model simplifications.

#### The instability of Chib’s method

In Sect. 6.3 we have seen that Chib’s method, naively applied as proposed in Chib and Jeliazkov (2001), can yield unstable results under certain circumstances. In our study we considered diffuse prior distributions, which can yield diffuse posterior distributions with undesired attractor states in the configuration space of the parameters. To see that, assume that in an MCMC chain, the parameters of the CheMA (or iCheMA) model have reached, by chance, a very large Michaelis–Menten parameter $k_{u,i}^{(r)}$ for a redundant species *u*, symbolically $k_{u,i}^{(r)}\rightarrow \infty$. We then have:

46 $$\begin{aligned} v_{u,i}^{(r)}\cdot x_u(t^{\star })\left( x_u(t^{\star })+k_{u,i}^{(r)}\right) ^{-1} \approx v_{u,i}^{(r)}\cdot 0 \approx 0 \end{aligned}$$

for all time points $t^{\star }$. That is, the design matrix column, corresponding to the redundant species *u*, becomes effectively a zero column having only a minor effect on the gradients of the response species *i*. The corresponding reaction rate parameter $v_{u,i}^{(r)}$ can then also take very large values without any significant negative effect on the model likelihood in Eq. (29). This, in turn, can yield an undesired positive feedback mechanism between $v_{u,i}^{(r)}\rightarrow \infty$ and $k_{u,i}^{(r)}\rightarrow \infty$. Consequently, a naive choice of the pivot parameter vector $\tilde{\varvec{\theta }}$ in Eq. (34) of Chib’s method, where the selected pivot parameters $\tilde{v}_{u,i}$ and $\tilde{k}_{u,i}$ for $v_{u,i}$ and $k_{u,i}$, respectively, substantially deviate from the MCMC sampled values with $v_{u,i}^{(r)}\rightarrow \infty , k_{u,i}^{(r)}\rightarrow \infty$, yields a significant underestimation of the denominator in Eq. (34), and thus a significant overestimation of the MLL for models with redundant species. The reason for the underestimation of $P(\tilde{\varvec{\theta }}|G,D)$ is that the selected pivot parameter vector $\tilde{\varvec{\theta }}$ ($\tilde{v}_{u,i}\in \tilde{\varvec{\theta }}$ and $\tilde{k}_{u,i}\in \tilde{\varvec{\theta }}$) is substantially different from the MCMC sampled parameters, where $v_{u,i}^{(r)}\rightarrow \infty$, and $k_{u,i}^{(r)}\rightarrow \infty$. Consequently, Chib’s method, as proposed in Chib and Jeliazkov (2001), significantly underestimates the posterior probability of the pivot parameters $\tilde{\varvec{\theta }}$.^9^ Attractor states indicative of diffuse posterior distributions cannot be avoided when diffuse prior distributions are used. However, a stabilization of Chib’s method can be achieved by selecting a pivot parameter vector $\tilde{\varvec{\theta }}$ that is representative for the sampled parameter values. In our simulation studies we never observed unstable results when selecting the pivot parameters $\tilde{\varvec{\theta }}$ with the highest posterior probability out of the set of actually sampled parameters (excluding the parameter states from the burn-in phase).

#### The inappropriateness of the g-prior

The inappropriateness of the g-prior, as demonstrated in Sect. 6.2, stems from the fact that the design matrices ${\mathbf{D}}_i$, defined in Sect. 2.3, have a very characteristic structure. The rows of the matrix ${\mathbf{D}}_i$ are given in Eq. (8) and it can be seen that the first column of ${\mathbf{D}}_i$, which represents the degradation, contains exclusively negative values, $(-x_i(t_1),\ldots ,-x_i(t_T))^{\top }$, while the subsequent columns contain only positive elements, $\frac{I_{u,i} x_u(t^{\star })+(1-I_{u,i})k_{u,i}}{x_u(t^{\star })+k_{u,i}}$ ($u\in \pi _i$ and $t^{\star }\in \{t_1,\ldots ,t_T\}$). Hence, the covariance structure $({\mathbf{D}}_i^{\top }{\mathbf{D}}_i)^{-1}$ of the g-prior imposes strong positive correlations between the degradation parameter $v_{0,i}$ and the other reaction rate parameters $v_{u,i}$ ($u\in \pi _i$) in the original CheMA model. Parameter constellations, such as $v_{0,i}<1<v_{u,i}$, where $v_{u,i}$ and $v_{0,i}$ are negatively correlated w.r.t. the prior in Eq. (11), are a priori penalized.^10^ The strength of those correlations diminishes with the number of regulators in $\pi _i$ (i.e. the number of covariates in ${\mathbf{D}}_i$), which is the reason why the g-prior systematically favours over-fitting models. Replacing the truncated g-prior from Eq. (11) by the ridge regression prior in Eq. (12), as proposed here, avoids a priori imposed correlations among the parameters altogether. This was found to significantly improve the model selection performance, as demonstrated empirically in Fig. 6.

#### Computational costs of CheMA and iCheMA

In addition to the better network reconstruction accuracy, e.g. demonstrated in Figs. 10 and 11, the improved variant of CheMA (iCheMA) also substantially reduces the computational costs of the MCMC-based inference, as shown in Table 2. The computational bottleneck of the original CheMA model of Oates et al. (2014) stems from the particular form of the prior distribution on the unknown parameters. The (truncated) multivariate Gaussian g-prior on the maximum reaction rate parameters $P_{\left\{ {\mathbf{V}}_i\ge 0\right\} }({\mathbf{V}}_i|\sigma _i^2,{\mathbf{D}}_i)$ in Eq. (11) depends on the values of the Michaelis–Menten parameters ${\mathbf{K}}_i$ via the design matrix ${\mathbf{D}}_i$=${\mathbf{D}}_i({\mathbf{K}}_i)$. As ${\mathbf{K}}_i$ can only be sampled with MH MCMC steps, see Eqs. (22, 23), each proposed move from ${\mathbf{K}}_{i}$ to ${\mathbf{K}}_{i}^{\star }$ also changes the prior distribution on ${\mathbf{V}}_i$. Hence, the MH acceptance probabilities in Eq. (23) includes the ratio:

47 $$\begin{aligned} \frac{P_{\left\{ {\mathbf{V}}_i\ge 0\right\} }({\mathbf{V}}_i|\sigma _i^2,{\mathbf{K}}_{i}^{\star })}{P_{\left\{ {\mathbf{V}}_i\ge 0\right\} }({\mathbf{V}}_i|\sigma _i^2,{\mathbf{K}}_{i})} = \frac{P({\mathbf{V}}_i|\sigma _i^2,{\mathbf{K}}_{i}^{\star })}{P({\mathbf{V}}_i|\sigma _i^2,{\mathbf{K}}_{i})} \cdot \frac{1-F({\mathbf{0}}|\sigma _i^2,{\mathbf{K}}_{i}) }{1-F({\mathbf{0}}|\sigma _i^2,{\mathbf{K}}_{i}^{\star })}\nonumber \\ \end{aligned}$$

where $P(.|\sigma _i^2,{\mathbf{D}}_i({\mathbf{K}}_{i}))$ and $F(.|\sigma _i^2,{\mathbf{D}}_i({\mathbf{K}}_{i}))$ are the probability density function and the cumulative distribution function (CDF) of the *untruncated* $\mathscr {N}( {\mathbf{1}}, n \sigma _i^2 ({\mathbf{D}}_i' {\mathbf{D}}_i)^{-1} )$ Gaussian distribution, and ${\mathbf{D}}_i={\mathbf{D}}_i({\mathbf{K}}_i)$. Hence, each proposal move on the Michaelis–Menten parameters ${\mathbf{K}}_i$ requires two CDFs $F({\mathbf{0}}|.)$ of multivariate Gaussian distributions to be computed. It is well-known that the computation of a Gaussian CDF with more than 3 dimensions (and an arbitrary covariance structure) is computationally challenging, and has for example to be done by randomized quasi Monte Carlo integration methods; see e.g. the algorithms proposed in Genz and Bretz (1999, 2002).^11^ The computational bottleneck of the inference algorithm is caused by the fact that this Monte Carlo approximation is required in each step of the MCMC simulation.

In the new variant (iCheMA) the truncated g-prior is replaced by a truncated ridge regression prior, see Eq. (12), so that the prior distribution of ${\mathbf{V}}_i$ becomes independent of ${\mathbf{K}}_i$ (see the graphical models in Fig. 3). The prior PR in Eq. (23) then reduces to Eq. (24). Hence, PR does not depend on multivariate Gaussian integrals (CDFs), and the computational bottleneck is avoided. See Table 2 for a comparison of the average run-times. The replacement of the g-prior by the ridge regression prior also ensures that the prior probabilities of ${\mathbf{V}}_i$ can be computed by factorization. The mean vector, $\varvec{\mu }={\mathbf{1}}$, and the covariance matrix, $\varvec{\varSigma }=\delta ^2_i \sigma _i^2{\mathbf{I}}$, of the truncated Gaussian prior in Eq. (12) imply:

48 $$\begin{aligned} F({\mathbf{0}}|{\mathbf{1}},\delta _i^2\sigma _i^2{\mathbf{I}}) = \left( F(0|1,\delta _i^2\sigma _i^2) \right) ^{|{\mathbf{V}}_i|} \end{aligned}$$

where $|{\mathbf{V}}_i|$ is the dimension of ${\mathbf{V}}_i$, and $F(.|\varvec{\mu },\varvec{\varSigma })$ denotes the CDF of a multivariate (or univariate) Gaussian distribution with mean $\varvec{\mu }$ and variance $\varvec{\varSigma }$.

### Appendix 2: Extended results

#### Gradient calculation with the Gaussian process

The Matlab package gpstuff (Vanhatalo et al. 2013) provides a convenient implementation of GP regression including several kernel functions and a procedure to optimize the kernel parameters with scaled conjugate gradient optimization of the MLL (fminscg). Prior to applying the GP we partitioned the data of the full mRNA time series of each species into smaller fragments that correspond to individual experiments. This is necessary in order to avoid artefacts that can arise from transitions between unrelated experiments. The fragments contain 13 time points over 24 h for the realistic data set (Sect. 5.2) and 12 data points over 48 h for the real data set (Sect. 5.3). For all kernels, except the periodic kernel (PER), we set the initial length scale parameter to the time interval between two data points, which resulted in 4 h for the realistic data, and 8 h for the real data set. The initial periodic length for the PER kernel was consistently set to a 12 h period length for both data sets. The Matérn class kernel was used in two variants defined by setting the hyperparameter to $\nu =3/2$ (MAT32), and $\nu =5/2$ (MAT52). The initial signal variance $\sigma ^2$ was set to 1 and the noise variance to $\sigma ^2_n = 0.1$ for all kernels. Note that the above values serve as an input to the optimization function gp_optim, which uses the optimization procedure fminscg as the default. Finally, we took the first derivative of each of the implemented kernel functions, replaced the original covariance matrix with the one derived from the derivative functions, and then applied Eq. (1) in Holsclaw et al. (2013) to obtain the expectation of the derivative for each time point.

In a preliminary study, we investigated the effect of the GP kernel on the network reconstruction accuracy. We used the realistic data of Sect. 5.2, obtained from the six network structures shown in Fig. 4, and compared four widely applied standard kernels: the radial basis function (or RBF) kernel, the periodic kernel, and two Matérn class kernels with different hyperparameters: $\nu =3/2$ (MAT32) and $\nu =5/2$ (MAT52). For mathematical details of these kernels, see Chapter 4 in Rasmussen and Williams (2006). To keep the computational costs of this pre-study low, we used the Lasso method (see Table 5) for network reconstruction, as described in Aderhold et al. (2014). The results are shown in Fig. 13. The performance of the periodic kernel is clearly the worst. The differences in the results for the other kernels are not significant. However, the RBF kernel shows, overall, the best performance, and was therefore used in all studies.

#### Inhibitor kinetics and decay term in CheMA

*Inhibitor kinetics.* The inhibitor kinetics defined in Eq. (5) of Oates et al. (2014) are slightly different from Eq. (6) in our paper in that the inhibitory effects enter the Michaelis–Menten term via a sum in the denominator. However, this equation is only a theoretical construct that was *not* actually used in the authors’ work. In Sect. 3.1 of their paper the authors write that they found inference for inhibitor sets to be extremely challenging, and that they therefore did not include any inhibitory regulation in the reaction graph. This implies that the sum in the denominator of Eq. (5) of their paper is zero. Consequently, the model defined in Eq. (6) of our paper is a generalization of CheMA, which subsumes CheMA in the limiting case of no inhibition. We repeated the simulations reported in our paper with a fan-in restriction of zero imposed on the inhibitor set, i.e. with all inhibitors removed, like in CheMA. The results are shown in Fig. 14 and clearly demonstrate that this leads to a significant drop in performance.

*Decay terms.* The CheMA model includes a Michaelis–Menten decay term for dephosphorylation:

49 $$\begin{aligned} \frac{dx_i(t)}{dt}|_{t=t^{\star }} \; = \; - \frac{\nu _{0,i} x_i(t^{\star })}{x_i(t^{\star }) + K_{0,i}} + \ldots \end{aligned}$$

Dephosphorylation is an active form of regulation mediated by phosphatases, hence saturation processes have to be taken into consideration. The corresponding effect in transcriptional regulation is mRNA degradation. As opposed to dephosphorylation, this is a passive decay that is commonly modelled with a linear decay term (Barenco et al. (2006)). In order to adapt the original CheMA model to transcriptional regulation, we have therefore replaced the Michaelis–Menten decay term by a linear decay term:

50 $$\begin{aligned} \frac{dx_i(t)}{dt}|_{t=t^{\star }} \; = \; -\nu _{0,i} x_i(t^{\star }) + \ldots \end{aligned}$$

see Eq. (5). This makes the model consistent with the biological literature; see e.g. Barenco et al. (2006) and Lawrence et al. (2010). To quantify the effect of this adaptation we have rerun the simulations reported in our manuscript with the linear decay term replaced by the Michaelis–Menten term used in CheMA. The results are shown in Fig. 15 and demonstrate that, in the context of transcriptional regulation, the linear term of Eq. (50) achieves overall better results than the Michaelis–Menten term of Eq. (49), with equal performance for four and a significant improvement for two of the six network structures included in the comparative analysis.

The upshot of these two studies is that if we apply CheMA exactly as reported in Oates et al. (2014), i.e. with Michaelis–Menten decay and no inhibition, the performance improvement achieved with the proposed new iCheMA model over CheMA would even be stronger than suggested by the results presented in our manuscript. However, to make the comparative analysis biologically realistic in the context of transcriptional regulation, it makes better sense to apply CheMA with the same modifications as for iCheMA, i.e. as reported in the manuscript.

#### Comparison with under-complex models

The results of a complementary study, where we compare the score differences (MLL versus IC) between the true and an under-complex model, are shown in Fig. 16. Unlike the previous study (Fig. 8), all methods succeed in identifying the true structure, indicating that the identification of an under-complex structure is substantially easier than the identification of an over-complex structure. While the IC score differences remain unaffected by the spread factor of the prior distribution ($sf$), the MLL score differences decrease with increasing $sf$, as a consequence of Lindley’s paradox. However, for the two scenarios considered here, even for spread factors as large as $sf=10^{20}$, MLL still allows the identification of the true structure, which suggests that the final outcome of model selection with MLL is not affected by Lindley’s paradox.

#### ANOVA

In Sects. 6.1, 6.2 and 6.5 of the main paper we evaluated the proposed improved CheMa model with respect to different effects, including the gradient response (Sect. 6.1), the choice of the prior distribution for the model parameters (Sect. 6.2), and the comparison of iCheMA to several state-of-the-art methods given realistic data sets (Sect. 6.5). These experiments involved a vast number of simulations and different setups. An example of one setup is shown in Fig. 17 for the method comparison of Sect. 6.5. This figure illustrates the complexity of the results, which makes it difficult to identify clear patterns and trends. In order to separate the effects of interest from the various confounding factors we adopted the DELVE evaluation scheme (Rasmussen (1996); Rasmussen et al. (1996)) and implemented a multi-way analysis (ANOVA, e.g. Brandt (1999)) as defined in Sect. 4.1.

To assess network reconstruction accuracy under different experimental conditions we first calculated AUROC and AUPREC scores that were used separately as response *y* in our ANOVA model:

51 $$\begin{aligned} y_{gnml} \; = \; G_g + N_n + M_m + \varepsilon _{gnml} \end{aligned}$$

The scores $y_{gnml}$ were collected for all factors included in our simulation studies: For the method comparison study (Sect. 6.5) this involved the type of gradient calculation *g*, the network topology *n*, the network reconstruction method *m*, and the data instantiation *l*. The index range for these parameters are $g \in \{0,1\}$, where $g=0$ denotes a gradient obtained from the difference quotient, and $g=1$ a gradient from a GP smoothing operation; $m \in \{0,1,2,3,4,5\}$, where $m=0$ represents the published network ‘wildtype’ and the five remaining indices represent modified topologies that simulate interventions of the wildtype network shown in Fig. 4; $n \in \{0,1,2,\ldots ,11\}$, for the 11 network reconstruction methods shown in Table 5 and Fig. 20, and five different data instantiations $l \in \{0,1,2,3,4\}$. The main effects were defined with discrete values denoted as $G_g$ for the different gradients, $N_n$ for the networks, and $M_m$ for the various reconstruction methods. The additive error was defined as zero-mean Gaussian noise with $\varepsilon _{ognmk} \sim N(0,\sigma ^2)$. Note that we modified Eq. (51) for the study in Sect. 6.2, where we considered the parameter prior ($P_p$) as main effect.

To confirm the validity of the ANOVA model, we tested the distribution of the residuals in terms of independence and identicality (i.i.d.). We carried out a standard residual analysis to assess whether the i.i.d. assumption was valid and the choice of this ANOVA model appropriate. A violation would indicate that the model in Eq. (51) was inadequate to fully capture the structure in the data, and that we would have to extend the model with, e.g. with higher-order terms.

To test the assumption of a Normal distribution, we created a QQ plot with the quantiles of the residuals of the AUROC and AUPREC scores plotted against the quantiles of a Normal distribution as shown in Fig. 18. Both plots show a good match along the dashed diagonal reference line with only minor deviations at the tails for the AUROC residuals (Fig. 18a). This suggests that the AUROC residuals have slightly heavier tails, although, an overall good agreement between the residual and Normal distributions can be observed.

The scatter plots in Fig. 19 show the residuals plotted against the fitted values from Eq. (51) for AUROC and AUPREC scores. The plots show overall even distributions that lack any discernible pattern or trend. This is consistent with the ANOVA model assumption that the distribution of the residuals does not depend on the main effects. Figs. 18 and 19 thus indicate that the ANOVA model of Eq. (51) is not in violation with the ANOVA model assumptions.

The plots in Fig. 20 and 21 show the results of the ANOVA approach for different effects in terms of the mean fitted AUROC and AUPREC values and corresponding confidence intervals. Fig. 20 displays the different methods $M_m$ as the main effect of the model with iCheMA outperforming all other methods. A comparison of reconstruction accuracy with respect to the different network structures $N_n$ in Fig. 21 shows that the accuracy increases with less complex interaction patterns. The effect of the gradient $G_g$ is displayed in Fig. 5a. It can be seen that the GP gradient dominates over the difference quotient based gradient.

### Appendix 3: Numerical stabilization

In Table 4 we have observed unexpected sharp increases of the MLL score difference obtained with the stabilized TI (TI-STAB) approach from Eq. (41). To explain the numerical instability of TI-STAB for large spread factors we consider a standard Bayesian linear regression model with a Gaussian distributed response variable *y*.

$$\begin{aligned} y \sim \mathscr {N}( \varvec{\pi }^{\top }\varvec{\beta }, \sigma ^2) \end{aligned}$$

where $\varvec{\pi }$ is a vector of $m+1$ covariates (including a 1 for the intercept), $\sigma ^2$ is the noise variance, and $\varvec{\beta }=(\beta _0,\ldots ,\beta _m)^{\top }$ is the $(m+1)$-dimensional vector of regression coefficients on which we impose a Gaussian prior:

52 $$\begin{aligned} p(\varvec{\beta }|\delta ^2,M) = (2\pi \delta ^2)^{-\frac{m+1}{2}} \cdot e^{- \frac{ \varvec{\beta }^{\top } \varvec{\beta }}{2\delta ^2} } \end{aligned}$$

where $\delta ^2$ is a hyperparameter. The likelihood for *T* data points $(y_1,{\mathbf{x}}_1^{\top }),\ldots ,(y_T,{\mathbf{x}}_T^{\top })$ is:

53 $$\begin{aligned} p({\mathbf{y}}|\varvec{\beta },M) = (2\pi \sigma ^2)^{-\frac{T}{2}} \cdot e^{-\frac{({\mathbf{y}} - {\mathbf{X}}\varvec{\beta })^{\top }({\mathbf{y}} - {\mathbf{X}}\varvec{\beta })}{2\sigma ^2}} \end{aligned}$$

where ${\mathbf{y}}=(y_1,\ldots ,y_T)^{\top }$ and ${\mathbf{X}}= ({\mathbf{x}}_1,\ldots ,{\mathbf{x}}_T)^{\top }$ is the design matrix. The marginal likelihood $p({\mathbf{y}}|\delta ^2,M)$ and the power posteriors $p(\varvec{\beta }|\delta ^2,{\mathbf{y}},\tau ,M)$ ($\tau \in [0,1]$) can then be computed in closed form:

54 $$\begin{aligned}&p({\mathbf{y}}|\delta ^2,M) = \int p({\mathbf{y}}|\varvec{\beta },M) p(\varvec{\beta }|\delta ^2,M) d\varvec{\beta }\nonumber \\&\quad = (2\pi )^{-\frac{T}{2}} \det \big (\sigma ^2 {\mathbf{I}} + \delta ^2 {\mathbf{X}} {\mathbf{X}}^{\top }\big )^{-\frac{1}{2}} \cdot e^{-\frac{1}{2} {\mathbf{y}}^{\top } \big (\sigma ^2 {\mathbf{I}} + \delta ^2 {\mathbf{X}} {\mathbf{X}}^{\top }\big )^{-1} {\mathbf{y}}} \nonumber \\\end{aligned}$$

55 $$\begin{aligned}&p\big (\varvec{\beta }|\delta ^2,{\mathbf{y}},\tau ,M\big ) = \frac{p({\mathbf{y}}|\varvec{\beta },M)^{\tau } p(\varvec{\beta }|\delta ^2,M)}{\int p({\mathbf{y}}|\varvec{\beta }',M)^{\tau } p(\varvec{\beta }'|\delta ^2,M) d\varvec{\beta }'}\nonumber \\&\quad = (2\pi )^{-\frac{m+1}{2}} \det (\varvec{\varSigma }_{\tau })^{-\frac{1}{2}} \cdot e^{-\frac{1}{2}(\varvec{\beta }-\mu _{\tau } )^{\top } \varvec{\varSigma }_{\tau }^{-1} (\varvec{\beta }-\mu _{\tau })} \end{aligned}$$

where $$\mu _{\tau } = \frac{\tau }{\sigma ^2} \varvec{\varSigma }_{\tau } {\mathbf{X}}^{\top } {\mathbf{y}}$$, and $$\varvec{\varSigma }_{\tau } = \sigma ^2 (\tau {\mathbf{X}}^{\top }{\mathbf{X}} + \frac{\sigma ^2}{\delta ^2} {\mathbf{I}})^{-1}$$.

For each $\tau \in \{\tau _1,\ldots ,\tau _K\}$ let $\varvec{\beta }_{\tau }^{(1)},\ldots ,\varvec{\beta }_{\tau }^{(J)}$ be a sample from Eq. (55). The logarithm of the marginal likelihood in Eq. (54) can then be approximated by TI and TI-STAB via Eqs. (37–41), using:

56 $$\begin{aligned}&\mathbb {E}_{\varvec{\beta },\tau _k}[\log (p({\mathbf{y}}|\delta ^2,M))] \approx \frac{1}{J} \sum _{j=1}^{J} \log (p({\mathbf{y}}|\delta ^2,\varvec{\beta }_{\tau _k}^{(j)},M)) \nonumber \\ \end{aligned}$$

57 $$\begin{aligned}&\mathbb {V}_{\varvec{\beta },\tau _k}(\log (p({\mathbf{y}}|\delta ^2,M))) \nonumber \\&\quad \approx \frac{1}{J} \sum _{j=1}^{J} (\log (p({\mathbf{y}}|\delta ^2,\varvec{\beta }_{\tau _k}^{(j)},M)) - \mathbb {E}_{\varvec{\beta },\tau _k}[\log (p({\mathbf{y}}|\delta ^2,M))] )^2\nonumber \\ \end{aligned}$$

For a comparison of the two thermodynamic integration approaches (TI and TI-STAB), see Eqs. (38–41), we compute Bayes factors $\mathscr {B}$ for a (true) linear model *M* with one covariate ($m=1$) against an over-complex model $\tilde{M}$ with two covariates (of which one is irrelevant). To this end, we we generate data from the true model. For $i=1,\ldots ,T$:

58 $$\begin{aligned} y_i = \beta _0 + \beta _1 x_{1,i} + \epsilon _i \end{aligned}$$

where $\epsilon _1,\ldots ,\epsilon _T$ are i.i.d. $N(0,\sigma ^2$)-distributed. We set $\beta _0=1$, $\beta _1=-1$, $\sigma ^2=1$, and $T=100$. We sample the values $x_{1,i},\ldots ,x_{1,T}$ of the covariate from standard Gaussian *N*(0, 1) distributions, and we build the true *n*-by-2 design matrix ${\mathbf{X}} = ({\mathbf{x}}_1,\ldots ,{\mathbf{x}}_T)^{\top }$, where ${\mathbf{x}}_i = (1,x_{1,i})^{\top }$. We compare the marginal likelihood $p({\mathbf{y}}|\delta ^2,M)$ of this true model with the marginal likelihood $p({\mathbf{y}}|\delta ^2,\tilde{M})$ of an over-complex model, for which we include a redundant covariable, having no effect on *y*. We sample its values $x_{2,1},\ldots ,x_{2,T}$ from independent *N*(0, 1) distributions. Using Eq. (54) we compute the exact (logarithmic) Bayes factors:

59 $$\begin{aligned} \mathscr {B}_{\delta ^2}(M,\tilde{M}) = \log \left( \frac{p({\mathbf{y}}|\delta ^2,M)}{p({\mathbf{y}}|\delta ^2,\tilde{M} )}\right) \end{aligned}$$

for increasing hyperparameters $\delta ^2=10^i$ $(i=-1,\ldots ,5$), and we compare them with the approximations $\mathscr {B}_{\delta ^2}(M,\tilde{M})$ obtained with TI and TI-STAB, see Eqs. (37–41), by plugging in the Monte Carlo estimates from Eqs. (56–57). As before we use the discrete temperatures $\tau _k=\left( \frac{k}{K}\right) ^m$ $(0\le k \le K)$ with $K=10$ and $m=8$, and for each temperature $\tau _k$ we take $J=10000$ samples from the power posteriors in Eq. (55) to compute Eqs. (56–57). This yields two estimates $\hat{\mathscr {B}_{\delta ^2}}(M,\tilde{M})_{TI}$ and $\hat{\mathscr {B}_{\delta ^2}}(M,\tilde{M})_{TI-STAB}$ for each $\delta ^2$.

In our study we compare the true log Bayes factors $\mathscr {B}_{\delta ^2}(M,\tilde{M})$, computed with Eq. (59), with estimates $\hat{\mathscr {B}_{\delta ^2}}(M,\tilde{M})$ approximated with TI and/or TI-STAB, respectively. Fig. 22a shows boxplots of the deviation scores:

60 $$\begin{aligned} \varDelta _{\mathscr {B}}(\delta ^2) = \mathscr {B}_{\delta ^2}(M,\tilde{M}) - \hat{\mathscr {B}_{\delta ^2}}(M,\tilde{M}) \end{aligned}$$

for both approaches, TI and TI-STAB, applied to 10 independent data instantiations.^12^ Fig. 23a shows the approximated Bayes factor $\hat{\mathscr {B}_{\delta ^2}}(M,\tilde{M})$ for one individual (representative) data example. Both figures reveal that TI systematically under-estimates the log Bayes factor, while TI-STAB over-estimates it. Fig. 23a shows that for small values of $\delta ^2$, TI-STAB gives indeed better predictions than its non-stabilized counterpart TI, as expected. However, for increasing values of $\delta ^2$, this trend is reverted, and the performance of TI-STAB drastically deteriorates as $log_{10}(\delta ^2)$ exceeds values of 3, rendering the numerical stabilization counter-productive. Since the systematic mismatches suggest that the approximation with the trapezium sum (without and with the correction term) with $K=10$ might be too rough, we fix $\delta ^2=10^5$ and increase the number of temperatures *K*. Fig. 22b shows boxplots of the $\varDelta _{\mathscr {B}}(\delta ^2)$ from Eq. (60) with $\delta ^2=10^5$ for the 10 data instantiations in dependence on *K*; again, the values $\varDelta _{\mathscr {B}}(\delta ^2)$ for one representative data example are shown in Fig. 23b. As expected, for both approaches the estimation accuracy increases with *K*, and for large *K* $(K\ge 16)$ TI-STAB becomes more accurate than TI (even for this most diffuse prior with $\delta ^2=10^5$). However, the overall conclusions is that contrary to the findings in Friel et al. (2013), TI-STAB does *not* offer a consistent improvement on TI, and we have identified scenarios where the use of the ’stabilization method’ is counter-productive.

To understand the reason for the failure of the stabilisation method, note the conceptual difference between the computation of the first and second derivative. The estimation of the first derivatives can be regarded as accurate, as long as the sample size for the approximation of the variance in Eq. (39) is sufficiently large. However, the second derivatives are approximated by a difference quotient; see Eq. (40). To reduce this error, increasing the sample size drawn from the power posterior is not enough; the number of discretization points needs also to be increased. If both the sample size and the number of discretization points are increased together, then the quadratic correction will indeed lead to higher accuracy. However, in many applications, the number of discretization points is fixed, e.g. when running a population MCMC simulation with a fixed number of processors/temperatures. Our study has revealed that for a fixed number of 10 discretization points (which is what we could afford in terms of computational costs), the discretization error inherent in the computation of the second derivatives can turn out to be counterproductive if the prior is vague.

To explore in more detail why TI-STAB can substantially fail for diffuse priors, we fix $K=10$ and $\delta ^2=10^5$, and analyse the approximation of the MLLs in more detail. The temperatures $\tau _0,\ldots ,\tau _{10}$ segment the interval [0, 1] into 10 disjunct sub-intervals, and we extract the interval-specific contributions to the MLL. Fig. 24 shows some diagnostics for the true model, applied to one typical data instantiation. The left panel compares the true interval-specific contributions and those approximated by TI. It can be seen that TI under-estimates the MLL by $-11$, where especially the contribution of the third interval, $[\tau _2,\tau _3]$, is under-estimated. The centre left panel of Fig. 24 shows the interval-specific ratios of the right- and the left-hand side of Eq. (40). It can be seen that the approximation is critically inaccurate for the third interval. The centre-right panel of Fig. 24 compares the interval-specific TI-STAB corrections and the correction terms that are required for compensation of the discretization error with TI. For the third interval the inaccurate approximation via Eq. (40) yields a correction term that is too large, i.e. a ’hypercompensation’ of the mismatch obtained with TI (see Fig. 24, left panel). Finally, the right panel of Fig. 24 shows the resulting interval-specific contributions of TI-STAB, where the inaccurate approximation via Eq. (40) has led to a ’hypercompensation’ of the moderate mismatch of TI (Fig. 24, left panel), i.e. to a more drastic over-estimation.

### Appendix 4: Real world application

#### Arabidopsis thaliana data

To predict a circadian regulatory network in *Arabidopsis thaliana* we use the DIURNAL data resource from the Mockler Lab (Mockler et al. 2007). The focus of the DIURNAL database are diurnal and circadian regulated genes that have been previously identified from microarray data using the Affymetrix ATH1 GeneChip platform (TAIR) in conjunction with the HAYSTACK tool (Mockler et al. 2007). Each selected gene in DIURNAL provides a compilation of up to 20 different time courses determined by different experimental conditions, which were produced by different laboratories. Each time series has gene expression samples from 12 consecutive measurements taken in 4 h intervals that cover 48 h. The experimental conditions include different settings such as 12 h light days, short days with 6 h of light, long days with 18 h of light, total darkness, constant light, different temperatures, and over-expressed genes. The background strain is predominantly Col-0^13^. We extracted the gene profiles for the following *A. thaliana* genes: *CCA1, LHY, PRR5, PRR7, PRR9, GI, TOC1, LUX, ELF3, ELF4, RVE8-1*, and *RVE8-2*, where the last two correspond to two different Affymetrix probes of *RVE8*. Although the gene *RVE8* is not an established component of the core clock, we are interested in the prediction of interactions with clock genes to compare our findings with a recent study by Fogelmark and Troein (2014). Since all 20 conditions were available for these genes, we were able to collect measurements for $T=240$ timepoints for each gene. We normalized the data for each laboratory separately, since some of the time courses were found to be on a different scale. Furthermore we added an artificial light variable to mimic a light spike at the beginning of the day.

#### Modelling transcriptional delays

Transcriptional regulation can be subject to time delays. When a transcription factor binds to the promoter of a gene to initiate its transcription, the transcribed mRNA is not available immediately, but only after a certain transition time required to assemble and release it. Mathematically, this can be modelled by modifying Eq. (5) as follows:

61 $$\begin{aligned} \frac{d x_i(t)}{dt}|_{t=t^{\star }}= c_i - v_{0,i} x_i(t^{\star }) + f_i(\pi _i(t^{\star }-\varDelta ),\varvec{\theta }) \end{aligned}$$

where $\varDelta$ is an additional parameter to allow for the fact that the effect of the transcriptional regulators on the regulatee is subject to the time delay $\varDelta$. The ensuing mathematical modifications are straightforward; for instance, Eq. (6) becomes

62 $$\begin{aligned} \frac{d x_i(t)}{dt}|_{t=t^{\star }}= & {} -v_{0,i} x_i(t^{\star }) \nonumber \\&+ \sum _{s\in \pi _i} v_{j,i} \frac{I_{j,i} x_s(t^{\star }-\varDelta )+(1-I_{j,i}) k_{j,i}}{x_s(t^{\star }-\varDelta )+k_{j,i}}\nonumber \\ \end{aligned}$$

and Eq. (8) generalizes to

63 $$\begin{aligned} {\mathbf{D}}_{i,t^{\star }}^{\top }= & {} \left( -x_i(t^{\star }),\frac{I_{1,i} x_1(t^{\star }-\varDelta )+(1-I_{1,i}) k_{1,i}}{x_1(t^{\star }-\varDelta )+k_{1,i}},\right. \nonumber \\&\left. \ldots ,\frac{I_{s,i} x_{s}(t^{\star }-\varDelta )+(1-I_{s,i}) k_{s,i}}{x_s(t^{\star }-\varDelta )+k_{s,i}}\right) \end{aligned}$$

In principle, we could now follow the same inference procedure as described before, with an augmented parameter vector in which $\varDelta$ has been included: ($\varvec{\theta }^\mathsf{T},\varDelta )^\mathsf{T}$. However, for the MLL we can use a ’trick’ from statistical phylogenetics to reduce the computational complexity. Denote by $p(D|G,\varDelta )$ the marginal likelihood for a fixed time delay $\varDelta$, obtained using the methods above with $\varDelta$, e.g. in Eqs. (62, 63), fixed. We need to integrate out $\varDelta$:

64 $$\begin{aligned} p(D|G) = \int p(D|G,\varDelta ) p(\varDelta ) d\varDelta \end{aligned}$$

Here, $p(\varDelta )$ is the prior distribution of the transcriptional delay $\varDelta$, whose support is the domain of the nonnegative real numbers, and for which the gamma distribution is an appropriate choice (see Fig. 25):

65 $$\begin{aligned} p(\varDelta )= & {} g(\varDelta |\alpha ,\beta ) \nonumber \\= & {} \frac{\beta ^{\alpha }}{\varGamma (\alpha )} \exp (-\beta \varDelta ) \varDelta ^{\alpha -1}; 0< \varDelta < \infty \end{aligned}$$

The integral in Eq. (64) is analytically intractable. Interestingly, such integrals routinely occur in phylogenetics, where the standard procedure is to approximate them by a discrete gamma distribution, as first proposed by Yang (1994). The idea is to introduce $k$ categories, each having approximately equal probability $1/k$, and approximate Eq. (64) by

66 $$\begin{aligned} p(D|G) \approx \frac{1}{k} \sum _{i=1}^{k} p(D|G,\varDelta _i) \end{aligned}$$

Yang (1994) suggested the following procedure for determining the $\varDelta _i$’s. Start with the percentage points corresponding to

67 $$\begin{aligned} (q_1,\ldots ,q_{k}) \; = \; \left( \frac{1}{2k}, \frac{3}{2k},\ldots ,\frac{2k-1}{2k}\right) \end{aligned}$$

and set

68 $$\begin{aligned} \tilde{\varDelta }_i \; = \; G^{-1}(q_i|\alpha ,\beta ) \end{aligned}$$

where $G^{-1}(.|\alpha ,\beta )$ is the inverse of the cumulative distribution function of the gamma density $g(.|\alpha ,\beta )$. Then, rescale all time delays, $\varDelta _i = \lambda \tilde{\varDelta }_i$, $\lambda > 0$, such that

69 $$\begin{aligned} \frac{1}{k}\sum _{i=1}^{k} \varDelta _i \; = \; \int _{0}^{\infty } \varDelta g(\varDelta |\alpha ,\beta ) d\varDelta \; = \; \frac{\alpha }{\beta } \end{aligned}$$

An illustration is provided in Fig. 25. Yang (1994) showed that in the context of phylogenetic inference, the discrete gamma method gives reliable results for the number of discrete categories as low as $k=3$, but the most common choice (which we use in our work) is $k=4$.

For the inclusion of time delays via Eq. (64) we need their prior distribution. We obtained a comprehensive list of transcriptional time delays from a literature study (Ota et al. 2003), displayed the distribution as a histogram, shown in Fig. 25, and fitted a gamma density to it; see Eq. (65). We then approximated the integral in Eq. (64) with the discrete gamma distribution method of Yang (1994), as discussed above. We chose $k=4$ discrete time delay categories, as recommended in Yang (1994); see Fig. 25.

#### Artificial light and data normalization

Light provides essential information that influences the regulatory system of the circadian clock. Proteins that are sensitive to light are known to convey this information to the clock. However, the real data described in Sect. 5.3 only provides “light versus darkness” information as binary descriptors and lacks quantities that more specifically reflect the action of light sensing components. The realistic Biopepa data simulations from Sect. 5.2 have shown that model prediction can be significantly improved by including an artificial light protein regulator called *Light*P*, as demonstrated in Fig. 26. *Light*P* resembles an artificial light spike in the beginning of the day and is composed of a binary light indicator multiplied by a hypothetical light sensing protein *P*; the latter was simulated with a Markov Jump Process following the procedure in Sect. 5.2. As an alternative to this approach we modelled the light influence as a change-point process that divides the data into a dark versus a light phase. Chib’s MLL is then calculated with iCheMA for each segment separately and summed up to the final MLL score. This model shows only minor and no significant improvement over the model that lacks any light information, as displayed in Fig. 26. Since the inclusion of *Light*P* has such a beneficial effect on model prediction, we decided to artificially generate this covariable with Biopepa^14^ for the real data. The change of interaction probabilities, when including *Light*P* into the real data, is illustrated in the scatter matrix of Fig. 27.

Another important factor is data normalization, which is commonly carried out with a Z-score transformation of the whole time series of a gene. However, the DIURNAL MocklerLab data in Sect. 5.3 include time series that stem from different labs and exhibit measurements on different scales. To avoid distortions due to extreme values we decided to apply separate Z-score transformations to those sets of time series that originate from the same publisher or lab (“lab-wise” normalization). Note that a more reliable approach would be to scale the gene measurements based on the expression level of one of the house keeping genes before applying the Z-score transformation. However, we found that the different labs did not consistently use the same house keeping genes. The scatter matrix in Fig. 27 shows the difference of the marginal posterior interaction probabilities using “lab-wise” normalization compared to the standard approach that uses Z-scores over all time-series of a single gene (“gene-wise”). Although there are some changes in the medium probability range, there is little change at the very low and very high probability score spectrum.

#### Results

There is substantial interest in understanding the molecular mechanism of circadian regulation, i.e. an organism’s internal time keeping. For plants in particular circadian regulation is essential to align the plant’s metabolism to the diurnal rhythm of day and night. In our final study, we applied the improved CheMA method (iCheMA) to gene expression time series from the circadian clock genes in the model plant *A. thaliana*. Since this is a real data set, we allowed for transcriptional time delays, as discussed above. Based on the previous results, we selected the marginal likelihood as the most reliable model selection criterion overall, and we applied Chib’s method with our numerical stabilization.

Various hypotheses about the structure of the central circadian gene regulatory network in *A. thaliana* have been published in the biological literature. For the evaluation of our predictions, we used two recently published network structures, which we refer to as the P2013 (Pokhilko et al. 2013) and the F2014 network (Fogelmark and Troein 2014). Taking these structures as a gold standard, we can evaluate the network reconstruction accuracy with standard ROC curves. The results are shown in Fig. 28. The figure compares the performance of two different GP kernels: the RBF kernel and the periodic kernel (see Chapter 4 in Rasmussen and Williams (2006)). The figure also evaluates the effect of explicitly modelling time delays. A further subtlety is the fact that the F2014 network includes a gene that is not included in the P2013 network (*RVE8*). For that reason we have evaluated the reconstruction accuracy for the F2014 network twice: with *RVE8* included and excluded. Fig. 28 also shows the AUROC, as well as the AUPREC as an alternative figure of merit.

With AUROC values ranging between 0.58 and 0.78, the agreement with the literature is significantly better than random expectation. The particular patterns found are as follows. The agreement with the F2014 network is better than with the P2013 network. The inclusion of time delays achieves a small improvement in terms of AUROC score (increase by 0.02), but not in terms of AUPREC scores. Unlike the results for the synthetic study, summarized in Fig. 13, the periodic kernel outperforms the RBF kernel.

To arrive at a particular network structure prediction, we took the marginal posterior probabilities obtained with the periodic kernel and the inclusion of time delays, corresponding to the top row in Fig. 28, and extracted all gene interactions above a selection threshold of 0.11; this value was found in Grzegorczyk et al. (2015) to lead to approximately the same number of gene interactions as in the P2013 network. The resulting network is shown in Fig. 29. To formally compare this prediction with the two networks from the literature, we created a Venn diagram, also shown in Fig. 29, which displays the number of interactions in the various intersections, e.g. the number of interactions predicted with iCheMA and found in P2013, but not in F2014 etc. Of the 15 gene interactions predicted with iCheMA in the way described above, shown in Fig. 29, 10 can be found in both the P2013 and the F2014 network, 2 can be found in the F2014 network, and only 3 do not agree with any of the published networks. This results are significantly better than random expectation and suggests that iCheMA provides a useful tool for hypothesis generation in molecular systems biology.

## Footnotes

1 Within this paper we refer to the resulting gradients as the *numerical* (Oates et al. 2014) and the *analytical* gradient, proposed here.

2 Using the whole distribution, as on page 58 of Holsclaw et al. (2013), would give us additional indication of uncertainty akin to a distribution of measurement errors. Due to the increased computational costs (additional matrix operations) this has not been attempted, though.

3 In the original CheMA model, a different decay term is used (for protein dephosphorylation), and no inhibitory interactions are included for numerical reasons. The linear decay term in Eq. (6) is more appropriate for transcriptional regulation, and the inclusion of inhibitory interactions achieves better results (as shown in Appendix 2).

4 This issue applies to the CheMA model, as proposed by Oates et al. and to the new improved variant (iCheMA), proposed here. For both models we implement the exact uncollapsed rather than the approximate collapsed Gibbs sampling step for $\sigma _i^2$.

5 The truncation of ${\mathbf{V}}_i$ is then automatically properly taken into account, as it will only be conditioned on ${\mathbf{V}}_i$ that fulfil the required constraint.

6 The HR is equal to 1, as the proposal moves are symmetric. New candidates ${\mathbf{K}}_i^{\star }$ with negative elements are never accepted, as they have the prior probability zero, $P({\mathbf{K}}_i^{\star })=0$.

7 In the realistic data study, we assume gene measurements to take place in intervals of $\delta _t = 2$ h. This mimics typical rates for the qRT-PCR sampling experiments in Flis et al. (2015).
